# Australia’s prehistoric ‘swamp king’: revision of the Plio-Pleistocene crocodylian genus *Pallimnarchus* de Vis, 1886

**DOI:** 10.7717/peerj.10466

**Published:** 2020-12-21

**Authors:** Jorgo Ristevski, Adam M. Yates, Gilbert J. Price, Ralph E. Molnar, Vera Weisbecker, Steven W. Salisbury

**Affiliations:** 1 School of Biological Sciences, The Univeristy of Queensland, Brisbane, QLD, Australia; 2 Museum of Central Australia, Museum and Art Gallery of the Northern Territory, Alice Springs, NT, Australia; 3 School of Earth and Environmental Sciences, The University of Queensland, Brisbane, QLD, Australia; 4 University of California Museum of Paleontology, Berkeley, CA, USA; 5 College of Science and Engineering, Flinders University, Adelaide, SA, Australia

**Keywords:** Pallimnarchus, Paludirex, Mekosuchinae, Crocodylia, Pliocene, Pleistocene, Cenozoic, Queensland, Australia

## Abstract

The crocodylian fossil record from the Cenozoic of Australasia is notable for its rich taxonomic diversity, and is primarily represented by members of the clade Mekosuchinae. Reports of crocodylian fossils from Australia date back to the late nineteenth century. In 1886, Charles Walter de Vis proposed the name *Pallimnarchus pollens* for crocodylian fossils from southeast Queensland—the first binomen given to an extinct crocodylian taxon from Australia. *Pallimnarchus* has come to be regarded as a large, broad-snouted crocodylian from Australia’s Plio-Pleistocene, and numerous specimens, few of which are sufficiently complete, have been assigned to it by several authors throughout the twentieth century. In the late 1990s, the genus was expanded to include a second species, *Pallimnarchus gracilis*. Unfortunately, the original syntype series described as *Pallimnarchus pollens* is very fragmentary and derives from more than one taxon, while a large part of the subsequently selected lectotype specimen is missing. Because descriptions and illustrations of the complete lectotype do not reveal any autapomorphic features, we propose that *Pallimnarchus pollens* should be regarded as a *nomen dubium*. Following this decision, the fossil material previously referred to *Pallimnarchus* is of uncertain taxonomic placement. A partial skull, formerly assigned to *Pallimnarchus pollens* and known as ‘Geoff Vincent’s specimen’, possesses many features of diagnostic value and is therefore used as basis to erect a new genus and species*—Paludirex vincenti* gen. et sp. nov. A comprehensive description is given for the osteology of ‘Geoff Vincent’s specimen’ as well as aspects of its palaeoneurology, the latter being a first for an extinct Australian crocodyliform. The newly named genus is characterized by a unique combination of premaxillary features such as a distinctive arching of the anterior alveolar processes of the premaxillae, a peculiar arrangement of the first two premaxillary alveoli and a large size disparity between the 3rd and 4th premaxillary alveoli. These features presently allow formal recognition of two species within the genus, *Paludirex vincenti* and *Paludirex gracilis* comb. nov., with the former having comparatively more robust rostral proportions than the latter. The *Paludirex vincenti* holotype comes from the Pliocene Chinchilla Sand of the Darling Downs, south-eastern Queensland, whereas the material assigned to *Paludirex gracilis* is from the Pleistocene of Terrace Site Local Fauna, Riversleigh, northwest Queensland. Phylogenetic analyses recover *Paludirex vincenti* as a mekosuchine, although further cladistic assessments are needed to better understand the relationships within the clade.

## Introduction

During most of the Cenozoic Era, Australia was inhabited by a rich crocodylian fauna that has almost all its taxa assigned to the clade Mekosuchinae (Willis, Molnar & Scanlon, 1993; Salisbury & Willis, 1996; Brochu, 2003; Yates & Pledge, 2016). The only extant crocodylians on the continent—*Crocodylus johnstoni* Krefft, 1873 and *Crocodylus porosus* Schneider, 1801—are exempt from this classification. Crocodylians currently referred to the Mekosuchinae are known from fossils recovered at many localities across Australia, with their temporal ranges spanning from the Eocene (Willis, Molnar & Scanlon, 1993; Salisbury & Willis, 1996; Holt, Salisbury & Willis, 2005; Buchanan, 2009) to the Pleistocene (Molnar, 1981; Willis & Molnar, 1997a). Mekosuchines also lived on some South Pacific islands, where they went extinct in the late Quaternary (Balouet & Buffetaut, 1987; Mead et al., 2002; Molnar, Worthy & Willis, 2002). Extinct crocodylians from the Cenozoic of Australia exhibit substantial morphological disparities between them, ranging from small (less than two meters in total length) to large-bodied (at least four meters in total length) taxa, as well as disparate snout varieties which include brevirostrine, longirostrine, and even altirostral (sensu Salisbury, 1994; =oreinirostral sensu Busbey, 1995) morphotypes that indicate different feeding adaptations (Willis, 2006).

Crocodylian remains from Australia’s prehistoric past began to be noted in the nineteenth century (Clarke, 1869; Daintree, 1872; Lydekker, 1888), with the first proposed binomial name for an extinct Australian crocodylian being *Pallimnarchus pollens* De Vis, 1886. This binomen was introduced in what is the earliest detailed descriptive publication on fossil crocodylians from Australia, authored by Charles Walter de Vis (born Devis, also known by his pen-name “Thickthorn”; see Ingram, 1990). By working with the craniomandibular elements as well as some osteoderms, De Vis (1886) came to suspect that the material he described belonged to an unrecognized species. However, he further wrote that “his acquaintance with the literature of the tertiary (sic.) and post-tertiary crocodilidae (sic.), does not suffice to assure him that it can not enter into any known genus”; and so, de Vis proposed the cabinet name *Pallimnarchus pollens* as “merely one of convenience” (De Vis, 1886, p. 191). Regardless of the informal establishment and lack of a designated holotype, the name *Pallimnarchus pollens* was used in the subsequent scientific and popular literature alike (Jack & Etheridge, 1892; Chapman, 1914, 1934; Longman, 1924; Howchin, 1930; Riek, 1952; Laseron, 1954; Stirton, Tedford & Woodburne, 1968; Hill, Playford & Woods, 1970; Steel, 1973; Bartholomai & Woods, 1976; Gorter & Nicoll, 1978; Molnar, 1979, 1982a; Sill, 1968 erroneously called it *Crocodylus pallimnarchus*), and some authors proceeded to refer additional material to the species (Gregory, 1903; De Vis, 1907; Longman, 1925, 1928, 1929; Jones, 1927; Anderson, 1937; Hecht, 1975 (the teeth referred to *Pallimnarchus pollens* in this study would later be recognized to be from a ziphodont crocodylian; see Hecht & Archer, 1977; Molnar, 1981, 2004; Willis & Molnar, 1997b); Archer & Wade, 1976). Finally, the taxon was revised by Molnar (1982b), who formalized it by providing a diagnosis and nominating a lectotype specimen (although it bears notice that he tentatively accepted the generic validity of *Pallimnarchus* De Vis, 1886; see p. 657 of Molnar, 1982b). Since, little attention was given to *Pallimnarchus* until Willis & Molnar (1997a; based on the work by Willis, 1995) provided the most recent and detailed survey of the taxon. Willis & Molnar’s (1997a) study was based on the assessment of multiple specimens, which allowed them to refine the diagnoses and also name a second species of *Pallimnarchus*, *Pallimnarchus gracilis* Willis & Molnar, 1997a. Studies devoted exclusively to *Pallimnarchus* have been rare (De Vis, 1886; Longman, 1925, 1928; Molnar, 1982b; Willis & Molnar, 1997a), and only a couple of articles published in the last two decades described fossils that were referred only tentatively to this genus (Mackness & Sutton, 2000; Mackness et al., 2010).

The name *Pallimnarchus* has traditionally been used in referral to large Australian crocodylians from the Plio-Pleistocene, characterized by broad platyrostral snouts (sensu Busbey, 1995) and inferred to have been semi-aquatic ambush predators that inhabited inland waterways (Molnar, 1982a, 1982b, 1991, 2004; Flannery, 1990; Willis & Molnar, 1997a; Webb, 2009, 2010, 2013; Smith, 2019). Fossils attributed to it in previous publications derive from various localities in several Australian states, yet according to some authors (notably Willis & Molnar, 1997a) the majority have been recognized from Queensland. The locality of discovery for most of the type material discussed by De Vis (1886) is unknown, other than that it probably came from somewhere on the Darling Downs, a geographical region in south-eastern Queensland (Molnar, 1982b). One specimen (QMF1752, also known as the “Lansdowne Snout”) that was originally assigned to *Pallimnarchus pollens* (Longman, 1925) was for a time treated as a fossil specimen of *C. porosus* (Molnar, 1982b), which led Molnar (1982a) to consider a possible generic synonymy between *Pallimnarchus* and *Crocodylus* Laurenti, 1768. However, this was reconsidered (Molnar, 1991), and the “Lansdowne Snout” was most recently referred to *Pallimnarchus gracilis* by Willis & Molnar (1997a). Initially, De Vis (1886) tentatively suggested alligatorid affinities for the original material he designated as *Pallimnarchus pollens*, but later studies favored a crocodylid assignment (Willis, Murray & Megirian, 1990; Megirian, Murray & Willis, 1991). Very few phylogenetic analyses have incorporated *Pallimnarchus* as an operational taxonomic unit (hence forth abbreviated as OTU), but those that have, recovered it as a mekosuchine crocodyloid (Willis, 1993, 1995; Salisbury & Willis, 1996; Molnar, Worthy & Willis, 2002; Yates & Pledge, 2016; Lee & Yates, 2018).

The genus *Pallimnarchus*, although known to science for well over a century, is still enigmatic and has proven difficult to diagnose due to scant remains, with the diagnoses based on few rostro-mandibular and dental elements. Crocodylian fossils from Plio-Pleistocene localities are abundant in museum collections (such as the Queensland Museum), and many of them, although unpublished, have been labelled as *Pallimnarchus* (J. Ristevski and Steven W. Salisbury, 2018, personal observations). Many of these remains are fragmentary and some are also in poor preservational condition, which makes their assignment to *Pallimnarchus* dubious. This circumstance necessitates an updated exhaustive review and revision of the taxon as to provide a better understanding of its taxonomic status and osteology. Ideally, this would be accomplished by studying a complete skeleton in order to have a viable reference specimen that would assist in disentanglement of the accessioned material. To the best of our knowledge, such a specimen, be it a complete skeleton or even a complete skull, is yet to be discovered and accessioned in a public collection. Instead, we aim to further elucidate the cranial osteology of crocodylians formerly accepted as *Pallimnarchus* through a detailed anatomical description of a specimen that was previously briefly discussed by Willis & Molnar (1997a). However, before we venture with the morphological description, some crucial issues with the type specimen, and consequently taxonomic status, of *Pallimnarchus* must be addressed.

### The taxonomic status of *Pallimnarchus pollens*

**Figure 1:**
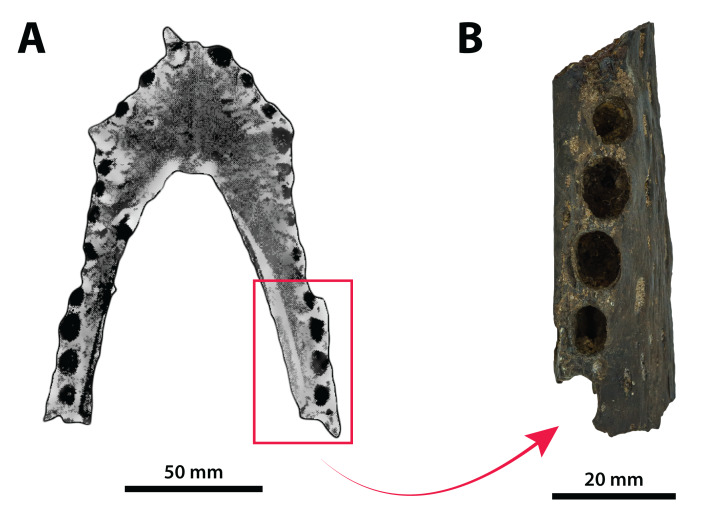
*Pallimnarchus pollens* De Vis, 1886, QMF1149, lectotype. (A) The complete lectotype specimen, with the portion of the recovered piece highlighted with the red rectangle, and (B) the only recovered piece of the lectotype. Figure in (A) modified from Plate 1, Figure B in Molnar (1982b).

The binomen *Pallimnarchus pollens* bears great historical significance as it is the first name offered for an extinct crocodylomorph from Australia. While this name was assigned informally by De Vis (1886), it has been widely used for over 130 years. And yet, the material referred to *Pallimnarchus* is confronted by several problems, with the first and perhaps biggest obstacle being the lack of a designated type specimen in the original publication by De Vis (1886). To complicate matters further, the original fossils described by De Vis (1886) do not belong to a single individual nor to a single species. These issues were recognized and addressed by Molnar (1982b), who proposed a lectotype—QMF1149, an anterior portion of a mandibular rostrum (Fig. 1A; see also Pl. 10, Fig. 1 in De Vis, 1886; and, Pl. 1, Figs. A and B in Molnar, 1982b). Molnar (1982b) selected QMF1149 to be the lectotype specimen not only because it was labelled as the type in the museum register, but also because of its comparatively more complete state relative to the other fragments discussed by De Vis (1886). However, most of QMF1149 has been missing since at least the late 1990s/early 2000s, and its current whereabouts are unknown. A thorough audit of the collection at the Queensland Museum undertaken in 2004 only managed to recover a small fragment from the right mandibular ramus of this specimen (J. Ristevski and K. Spring, 2019, personal communication). Unfortunately, the remaining fragment of QMF1149 (Fig. 1B) is non-diagnostic and of minimal comparative value. Until, and if, the rest of QMF1149 is found, the only source of information on it are the descriptions and photographs provided by De Vis (1886) and Molnar (1982b).

The now (mostly) missing *Pallimnarchus pollens* lectotype specimen (QMF1149) was an incomplete mandible belonging to a small crocodylian, interpreted by Molnar (1982b) to have been from an immature individual that was probably around three meters in total length (hence forth abbreviated as TL). When establishing his initial diagnosis for *Pallimnarchus pollens*, Molnar (1982b) relied on comparisons with only four crocodylians—*Crocodylus johnstoni*, *C. novaeguineae* (Schmidt, 1928), *C. porosus* and the then recently erected *Quinkana fortirostrum* Molnar, 1981. That was certainly a sound assessment for the time, as these five taxa were the only recognized crocodylians from Australasia’s Cenozoic (in addition to *Crocodylus nathani* Longman, 1924, which was declared a junior subjective synonym of *C. porosus* in the same article by Molnar, 1982b). Distinguishing QMF1149 from the other four crocodylians was simple, as its proportions were clearly different from those of the extant *Crocodylus* and it was also not referable to an altirostral taxon like *Quinkana* Molnar, 1981. Thus, *Pallimnarchus pollens* was acknowledged as a valid species. However, the past three decades have experienced a surge in discoveries of new crocodylian taxa not only from the Australian Cenozoic, but globally. Therefore, the mere proportional differences between QMF1149 and extant *Crocodylus* are insufficient, and unambiguous autapomorphic morphological features are required to properly characterize *Pallimnarchus pollens* based on QMF1149. Regrettably, we could not identify any autapomorphies in QMF1149 based on the descriptions and illustrations by De Vis (1886) and Molnar (1982b), or from first-hand examination of the remaining lectotype fragment. Additionally, the recent mentions of a new broad-snouted species of *Crocodylus* from Pliocene deposits in South Australia that shares similar proportions with QMF1149 (Yates & Pledge, 2016; Yates, 2019) would invalidate any diagnosis of *Pallimnarchus* that was based on rostral proportions alone. Because the lectotype is both lost and non-diagnostic, we consider *Pallimnarchus* to sit in taxonomic limbo. Due to these circumstances there is little choice but to relegate the name *Pallimnarchus pollens* to the status of *nomen dubium*.

## Materials and Methods

### Specimens

**Figure 2:**
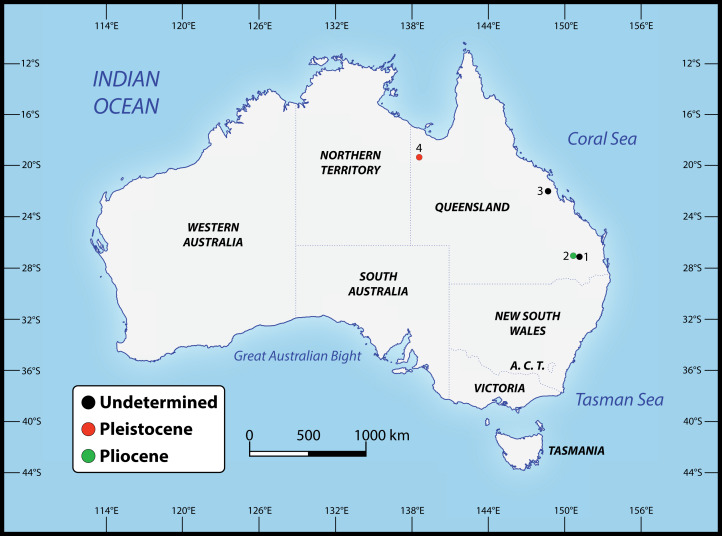
Localities across Queensland, Australia, that have yielded the specimens referred to *Paludirex* gen. nov. 1. Darling Downs region, near Warra (QMF11626); 2. Chinchilla (‘Geoff Vincent’s specimen’); 3. Nebo district, near Mackay (PVM 89-1072); 4. Terrace Site, Riversleigh (QMF17065 and QMF17066).

Numerous crocodylian specimens of both extant and extinct taxa were examined first-hand, not only for the purpose of the anatomical descriptions and comparisons presented herein, but also for scoring the morphological characters for the phylogenetic analyses. The studied specimens are housed in several institutions across Australia, for which we were kindly granted permissions to visit. A complete list of all specimens that were inspected can be found in the Supplemental Document S1 to this article (see the Data Availability section). Regarding the undertaken taxonomic revision, we identified five specimens that can be confidently assigned to the new genus (see Table 1). The specimens that we determined to pertain to the novel taxon derive from several localities in Queensland, Australia (Fig. 2). Our rationale for assigning them to the herein newly named genus is explained throughout the rest of the text.

**Table 1:** List of all specimens referred to *Paludirex* gen. nov. as recognized in this study.

Specimen	Description	Previous taxonomic referral	Current taxonomic referral
‘Geoff Vincent’s specimen’ (CMC2019-010 + QMF59017)	partial skull	*Pallimnarchus pollens* (Willis, 1995; Willis & Molnar, 1997a)	*Paludirex vincenti* (holotype)
PVM 89-1072	partial skull	*Pallimnarchus pollens* (Molnar, 1991) *Pallimnarchus gracilis* (Willis, 1995, 2006; Willis & Molnar, 1997a; Molnar, 2004)	*Paludirex* cf. *P. vincenti*
QMF11626	left premaxilla and partial maxilla	*Crocodylus porosus* (Molnar, 1982b; Lees, 1986) *Pallimnarchus pollens* (Willis & Molnar, 1997a) *Pallimnarchus* sp. (Chiotakis, 2018)	*Paludirex vincenti*
QMF17065	right premaxilla	*Pallimnarchus gracilis* (Willis, 1995, holotype; Willis & Molnar, 1997a, holotype)	*Paludirex gracilis* (holotype)
QMF17066	anterior fragment of a left dentary	*Pallimnarchus gracilis* (Willis, 1995; Willis & Molnar, 1997a)	*Paludirex gracilis*

### Computed tomographic scanning and 3D digital models

In order to enhance the morphological description, we relied on data obtained via computed tomography (CT) scans from the most complete and best-preserved sample of the new taxon—‘Geoff Vincent’s specimen’ (CMC2019-010 + QMF59017). Because the skull pieces of the ‘Geoff Vincent’s specimen’ are currently housed in two different museums (see “Specimen Background” subsection below), they were CT scanned separately and on two different occasions. Details on the CT scanning settings and parameters are given in the Supplemental Document S2 (Table S2.1) of this article (see the Data Availability section).

The images acquired from the CT scan are DICOM files that were imported into the specialized 3D image processing software Mimics 22.0 (Materialise NV, Belgium) at The University of Queensland. Digital models of the skull pieces were generated in Mimics 22.0, where the endocranial features were manually (and seldomly semi-automatically, by having the Auto interpolate option activated) segmented using several tools from the SEGMENT menu. Afterwards, the digital models of the skull pieces and segmented endocranial features were exported as STL (stereolithography) files. The STL files of the segmented endocranial structures were then imported into Materialise 3-matic 14.0 in order to create the interactive 3D PDF of the ‘Geoff Vincent’s specimen’ that is added as a supplement to this article.

### Mensuration

The external linear measurements on the studied specimens were taken directly using a hand-held 150 millimeter (mm) digital caliper. Where the dimensions exceeded 150 mm, a 5 meter (m) measuring tape was used instead. The digitally segmented endocranial structures were measured in Mimics 22.0 using the Distance tool from the MEASURE menu.

### Anatomical terminology, orientation and abbreviations

The anatomical terminology, abbreviations and descriptive approach used herein follow the example set by Kley et al. (2010). In their anatomical description on the skull and mandibles of the notosuchian crocodyliform *Simosuchus clarki* Buckley et al., 2000, Kley et al. (2010) devised a comprehensive descriptive layout that is applicable to osteological assessments of other crocodyliforms. Therefore, like in Kley et al. (2010), virtually all anatomical terms used here are in English instead of standard Latin. Certain anatomical structures have received more categorical descriptions in recent years, such as the crocodylomorph laterosphenoid in the publication by Holliday & Witmer (2009)—in that particular case, we follow the latter study instead of Kley et al. (2010). Traditional (or “Romerian”, after Romer, 1956) directional terms are used throughout, such as “anterior” and “posterior” vis-à-vis their veterinary correspondents “rostral” and “caudal”. Exempt from these directional phrases is the dental terminology, to which “mesial” and “distal” are applied instead of “anterior” and “posterior”, and “labial” and “lingual” as opposed to “lateral” and “medial”. Dental definitions are after Hendrickx, Mateus & Araújo (2015). Even though Hendrickx, Mateus & Araújo (2015) reviewed the dentition of non-avian theropod dinosaurs, their terminology is largely adaptable to crocodylomorphs as well. The terminology for the endocranial structures is mostly based on Witmer et al. (2008), and in the case of the pneumatic sinuses after Dufeau & Witmer (2015).

### Phylogenetic analyses

The maximum parsimony phylogenetic analyses were carried out in TNT v1.5 Willi Hennig Society Edition (Goloboff, Farris & Nixon, 2008; Goloboff & Catalano, 2016). The program was set to 900 Mb of RAM, with the maximum number of held trees being 99,999. The parameters applied in the analyses follow Young et al. (2016), which implement the new technology searches (sectorial search, ratchet, drift, and tree fusion) set to 1000 random addition sequences (RAS). For the sectorial search, the selection size above 75 used 1,000 drifting cycles, 1,000 starts below 75 and trees were fused 1,000 times. In addition, the consensus sectorial search (CSS) and exclusive sectorial search (XSS) were set to 1,000 rounds. For ratchet, the parameters were set to stop the perturbation phase when 1,000 substitutions were made, or 99% of the swapping was completed and a total of 1,000 iterations. For drift, the perturbation phase stopped when 1,000 substitutions were made, or 99% of the swapping was completed, and the number of cycles was set to 1,000. No alterations were made to the tree fusion settings which were left at the default three rounds.

Nodal support was assessed by conducting Bremer support and bootstrap analyses. The Bremer support was performed by running the script “BREMER.RUN”, which is provided with the TNT v1.5 download package, and used the default settings. The bootstrap analysis (Efron, 1979; Felsenstein, 1985) was set to 1,000 replicates, showing values of 50% and above.

Two homoplasy metrics, the consistency index (CI; Kluge & Farris, 1969) and retention index (RI; Farris, 1989), were calculated by running the script “STATS.RUN”, also provided in the TNT v1.5 download package.

### Nomenclatural acts

The electronic version of this article in Portable Document Format (PDF) will represent a published work according to the International Commission on Zoological Nomenclature (ICZN), and hence the new names contained in the electronic version are effectively published under that Code from the electronic edition alone. This published work and the nomenclatural acts it contains have been registered in ZooBank, the online registration system for the ICZN. The ZooBank LSIDs (Life Science Identifiers) can be resolved and the associated information viewed through any standard web browser by appending the LSID to the prefix http://zoobank.org/. The LSID for this publication is: (urn:lsid:zoobank.org:pub:402E681F-7C0A-4746-A80B-ADB4870789EC). The online version of this work is archived and available from the following digital repositories: PeerJ, PubMed Central and CLOCKSS.

## Systematic Paleontology

CROCODYLOMORPHA Hay, 1930 (sensu Nesbitt, 2011)

CROCODYLIFORMES Hay, 1930 (sensu Sereno et al., 2001)

MESOEUCROCODYLIA Whetstone & Whybrow, 1983 (sensu Sereno et al., 2001)

EUSUCHIA Huxley, 1875

CROCODYLIA Gmelin, 1789 (sensu Benton & Clark, 1988)

CROCODYLOIDEA Fitzinger, 1826 (sensu Brochu, 2003)

MEKOSUCHINAE (Balouet & Buffetaut, 1987)

*PALUDIREX* GEN. NOV. (Figs. 3–33)

**Figure 3:**
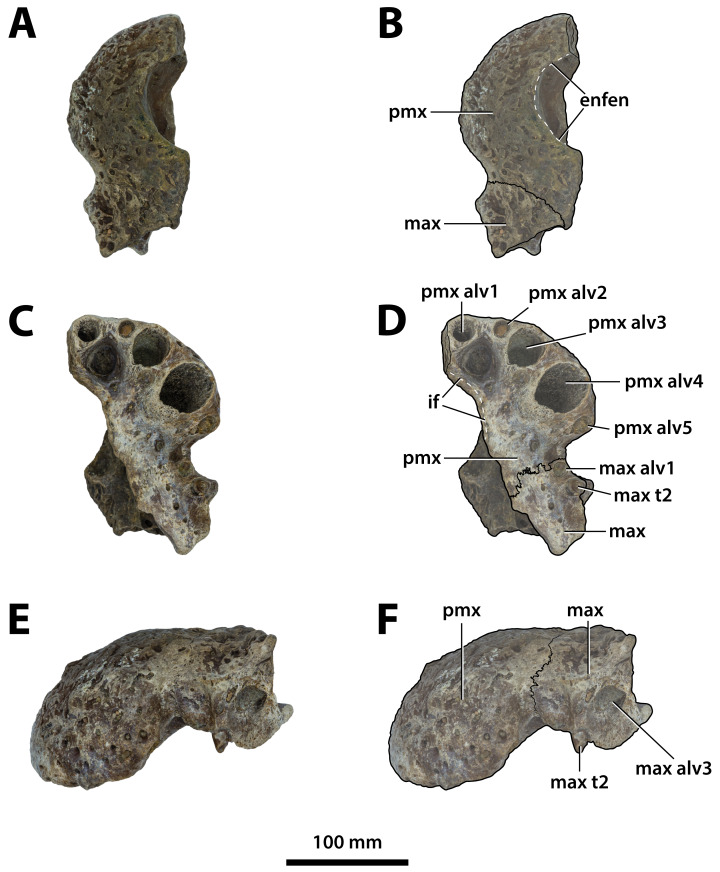
*Paludirex vincenti* gen. et sp. nov., QMF11626, referred specimen, left premaxilla and partial maxilla. Dorsal view of the specimen (A) non-annotated photograph, and (B) annotated photograph. Ventral view of the specimen (C) non-annotated photograph, and (D) annotated photograph. Left lateral view of the specimen (E) non-annotated photograph, and (F) annotated photograph. Abbreviations: enfen, external narial fenestra; if, incisive foramen; max, maxilla; max alv, maxillary alveolus; max t, maxillary tooth; pmx, premaxilla; pmx alv, premaxillary alveolus.

**Figure 4:**
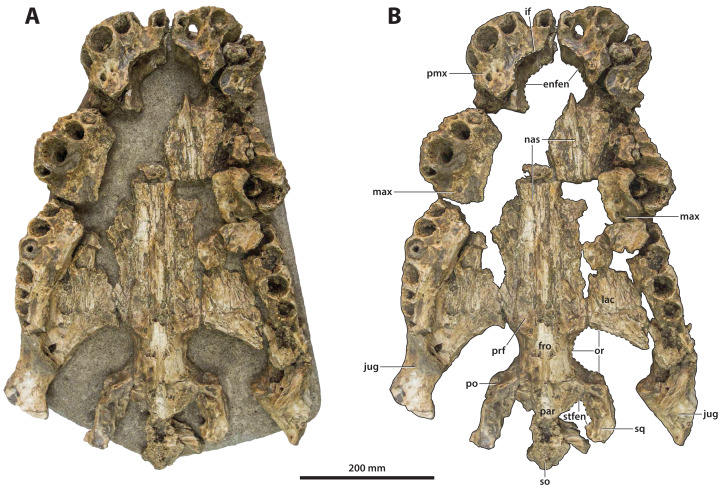
*Paludirex* cf. *P. vincenti*, PVM 89-1072, partial skull in ventral view. (A) Non-annotated photograph of the specimen as embedded in a concrete slab, and (B) annotated photograph, with the concrete slab digitally removed. Abbreviations: enfen, external narial fenestra; fro, frontal; if, incisive foramen; jug, jugal; lac, lacrimal; max, maxilla; nas, nasals; or, orbit; par, parietal; pmx, premaxilla; po, postorbital; prf, prefrontal; so, supraoccipital; sq, squamosal; stfen, supratemporal fenestra.

**Figure 5:**
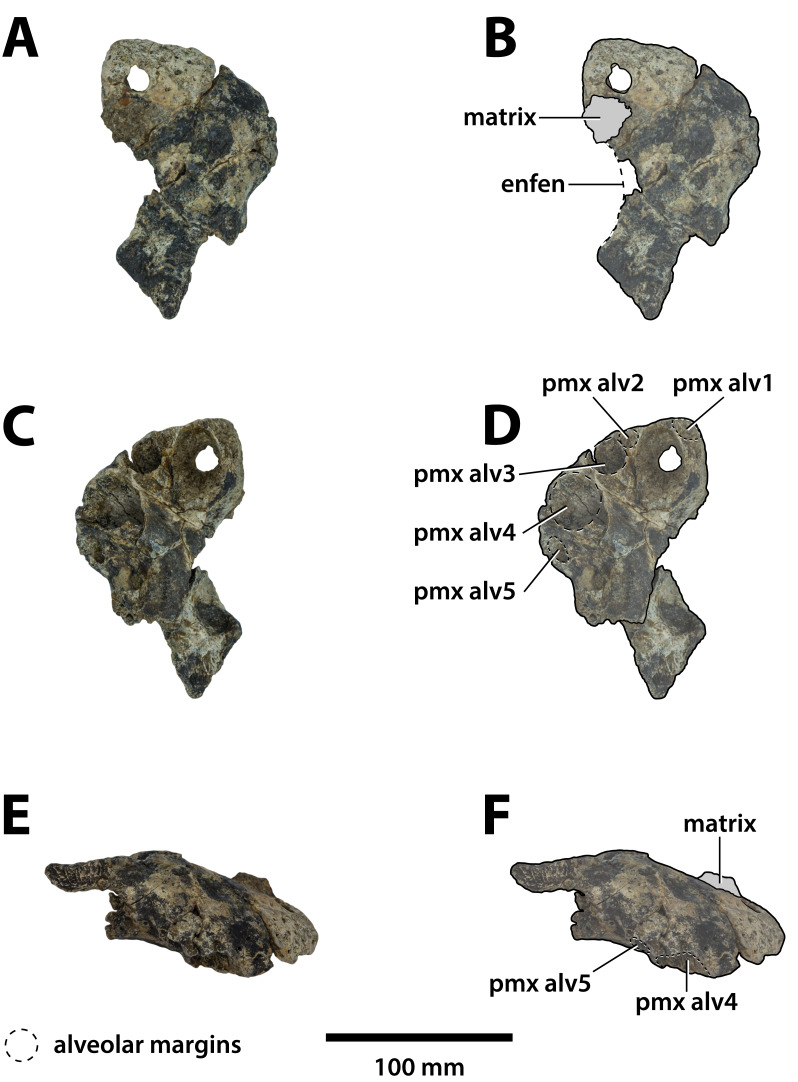
*Paludirex gracilis* gen. et comb. nov., QMF17065, holotype, right premaxilla. Dorsal view of the specimen (A) non-annotated photograph, and (B) annotated photograph. Ventral view of the specimen (C) non-annotated photograph, and (D) annotated photograph. Right lateral view of the specimen (E) non-annotated photograph, and (F) annotated photograph. Abbreviations: enfen, external narial fenestra; pmx alv, premaxillary alveolus.

**Figure 6:**
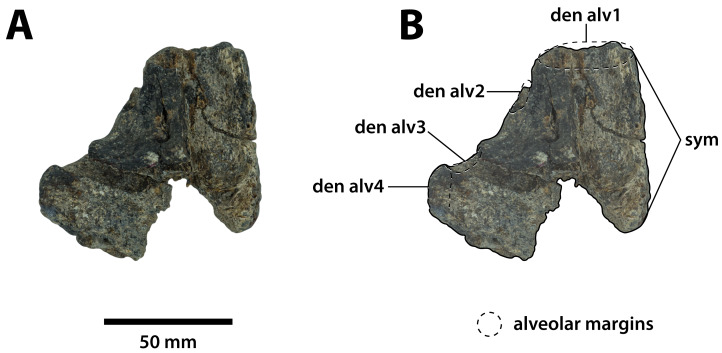
*Paludirex gracilis* gen. et comb. nov., QMF17066, referred specimen, anterior dentary fragment. Dorsal view of the specimen (A) non-annotated photograph, and (B) annotated photograph. Anterior is towards the top of the figure. Abbreviations: den alv, dentary alveolus; sym, mandibular symphysis.

**Figure 7:**
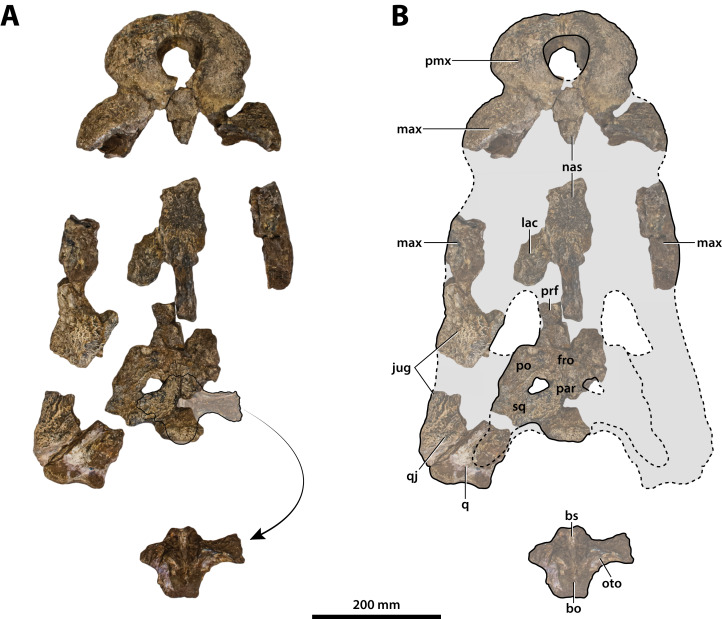
*Paludirex vincenti* gen. et sp. nov., ‘Geoff Vincent’s specimen’ (CMC2019-010 + QMF59017), holotype, all skull pieces in dorsal view. (A) Non-annotated photograph, and (B) annotated photograph. The arrow in (A) points to a clearer view of the basicranium (QMF59017) from its approximate anatomical position ventral to the cranial table (CMC2019-010-5). The dashed lines in (B) highlight the hypothetical outline of the skull. Abbreviations: bo, basioccipital; bs, basisphenoid; fro, frontal; jug, jugal; lac, lacrimal; max, maxilla; nas, nasals; oto, otoccipital; par, parietal; pmx, premaxilla; po, postorbital; prf, prefrontal; q, quadrate; qj, quadratojugal; sq, squamosal.

**Figure 8:**
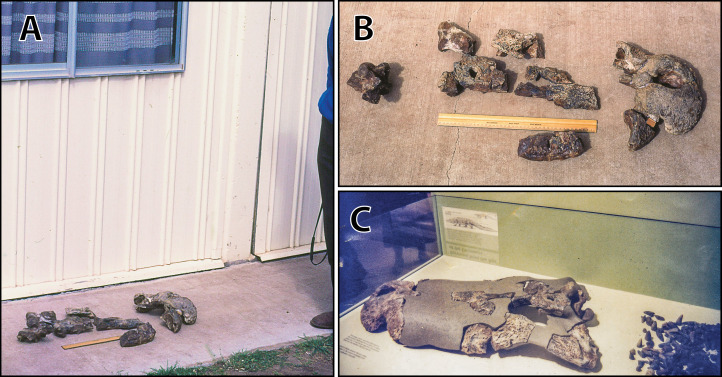
*Paludirex vincenti* gen. et sp. nov., ‘Geoff Vincent’s specimen’ (CMC2019-010 + QMF59017), holotype—specimen background. (A) and (B) ‘Geoff Vincent’s specimen’ as photographed at Mr. Vincent’s house, and (C) the specimen as photographed on display at the Queensland Museum sometime in the 1990s. Several pieces are missing in B (also in A, albeit less apparent), specifically, the posterior portion of the left maxilla (from CMC2019-010-4); the cranial table unit (CMC2019-010-5) is missing its right half; and, the quadratojugal & posterior jugal process unit (CMC2019-010-7). The reason why these pieces are not in the photographs is unknown, as they are obviously present in (C) when the specimen was publicly displayed. Also notice a small unidentified piece glued posteriorly to the lacrimal (B) that is not depicted in the display mount (C) and could not be located with the specimen as examined for the purposes of this study. Photographs in (A) and (B) taken August 1988 by Ralph E. Molnar; photograph in (C) property of the Chinchilla Museum, used with permission.

**Figure 9:**
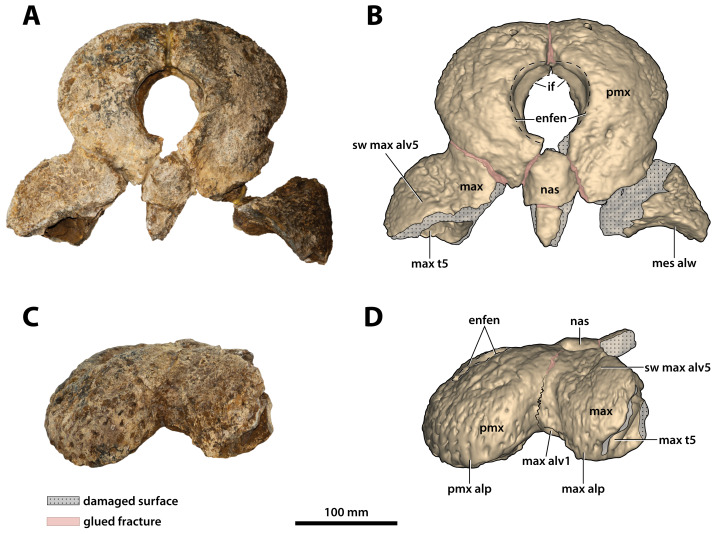
*Paludirex vincenti* gen. et sp. nov., ‘Geoff Vincent’s specimen’ (CMC2019-010-2), holotype, anterior rostrum. Anterior rostrum in dorsal view (A) photograph, and (B) annotated digital model. Anterior rostrum in left lateral view (C) photograph, and (D) annotated digital model. Abbreviations: enfen, external narial fenestra; if, incisive foramen; max, maxilla; max alp, maxillary alveolar process; max alv, maxillary alveolus; max t, maxillary tooth; mes alw, damaged mesial alveolar wall; nas, nasals; pmx, premaxilla; pmx alp, premaxillary alveolar process; sw max alv5, swelling over 5th maxillary alveolus.

**Figure 10:**
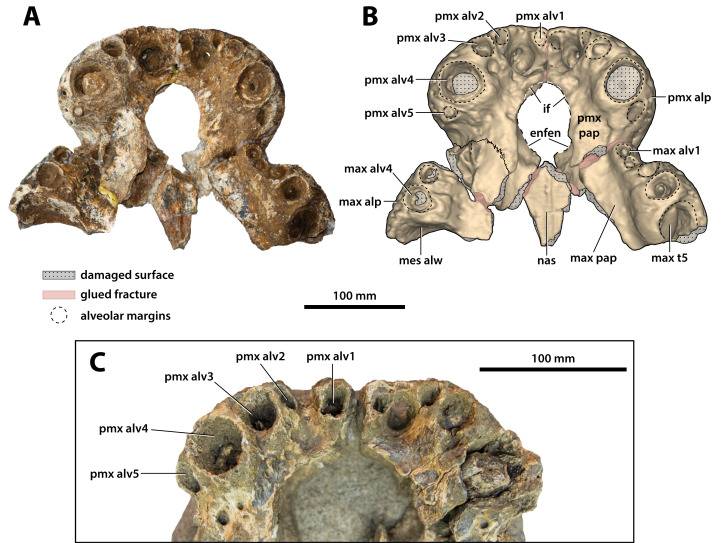
*Paludirex vincenti* gen. et sp. nov., anterior rostrum in ventral view. *Paludirex vincenti* gen. et sp. nov., ‘Geoff Vincent’s specimen’ (CMC2019-010-2), holotype, (A) photograph, and (B) annotated digital model. (C) *Paludirex* cf. *P. vincenti*, PVM 89-1072, close-up of premaxillae in ventral view. Abbreviations: enfen, external narial fenestra; if, incisive foramen; max alp, maxillary alveolar process; max alv, maxillary alveolus; max pap, maxillary palatal process; max t, maxillary tooth; mes alw, damaged mesial alveolar wall; nas, nasals; pmx alp, premaxillary alveolar process; pmx alv, premaxillary alveolus; pmx pap, premaxillary palatal process.

**Figure 11:**
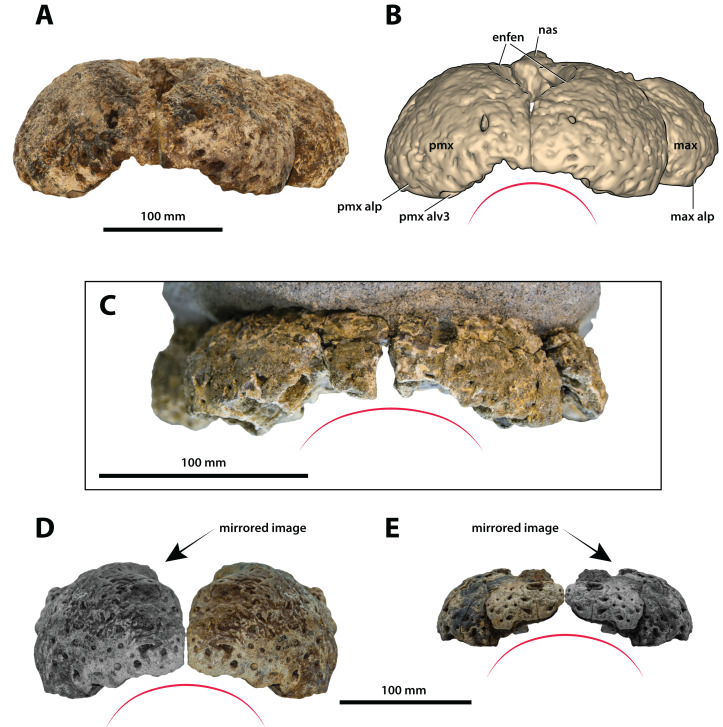
*Paludirex* gen. nov., rostra in anterior view. *Paludirex vincenti* gen. et sp. nov., ‘Geoff Vincent’s specimen’ (CMC2019-010-2), holotype, (A) photograph, and (B) annotated digital model. (C) *Paludirex* cf. *P. vincenti*, PVM 89-1072, premaxillae in anterior view. (D) *Paludirex vincenti* gen. et sp. nov., QMF11626, referred specimen, premaxilla in anterior view. (E) *Paludirex gracilis* gen. et comb. nov., QMF17065, holotype, premaxilla in anterior view. The curved red lines indicate the anterior arching of the premaxillae. The indicated grayscale images in (D) and (E) are mirrored in order to aid comparison. Abbreviations: enfen, external narial fenestra; max, maxilla; max alp, maxillary alveolar process; nas, nasals; pmx, premaxilla; pmx alp, premaxillary alveolar process; pmx alv, premaxillary alveolus.

**Figure 12:**
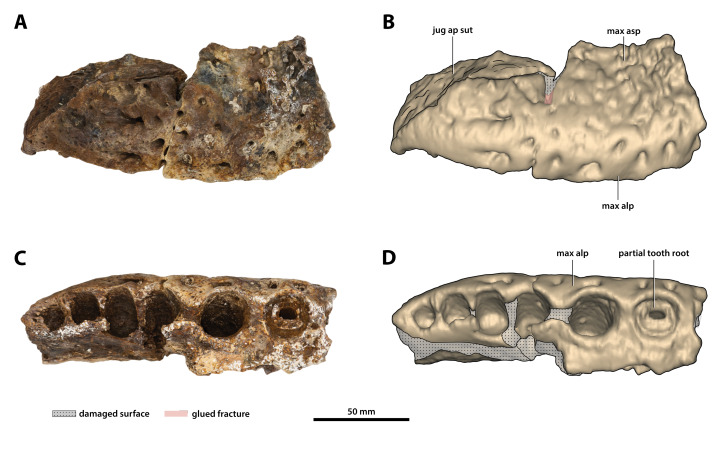
*Paludirex vincenti* gen. et sp. nov., ‘Geoff Vincent’s specimen’ (CMC2019-010-1), holotype, posterior piece from the right maxilla. (A) Photograph, and (B) annotated digital model in lateral views. (C) Photograph, and (D) annotated digital model in ventral views. Abbreviations: jug ap sut, sutural surface on maxilla for articulation with the anterior process of the jugal; max alp, maxillary alveolar process; max asp, maxillary ascending process.

**Figure 13:**
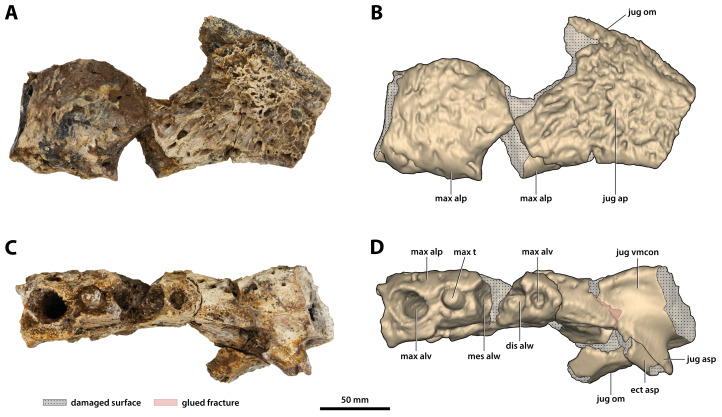
*Paludirex vincenti* gen. et sp. nov., ‘Geoff Vincent’s specimen’ (CMC2019-010-4), holotype, left anterior jugal process and posterior piece from the left maxilla. (A) Photograph, and (B) annotated digital model in lateral views. (C) Photograph, and (D) annotated digital model in ventral views. Abbreviations: dis alw, damaged distal alveolar wall; ect asp, ectopterygoid ascending process; jug ap, jugal anterior process; jug asp, jugal ascending process; jug om, jugal orbital margin; jug vmcon, jugal ventromedial concavity; max alp, maxillary alveolar process; max alv, maxillary alveolus; max t, maxillary tooth; mes alw, damaged mesial alveolar wall.

**Figure 14:**
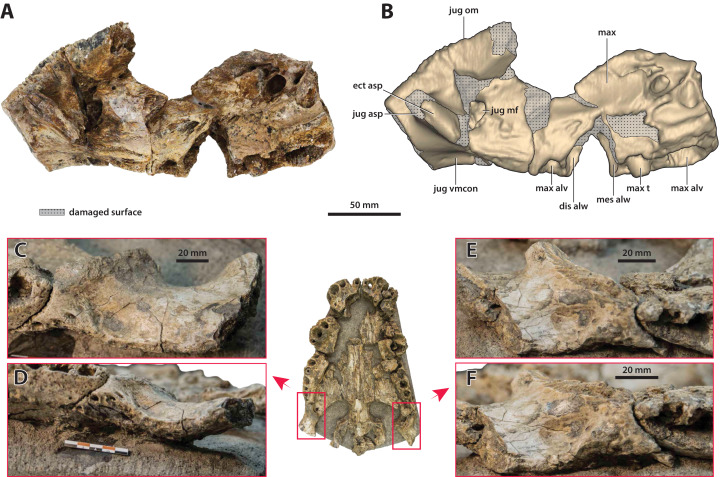
*Paludirex vincenti* gen. et sp. nov., details on the jugals. ‘Geoff Vincent’s specimen’ (CMC2019-010-4), holotype, (A) photograph, and (B) annotated digital model of the left anterior jugal process and posterior piece from the left maxilla in medial views. *Paludirex* cf. *P. vincenti*, PVM 89-1072, photographs of the right jugal in (C) oblique ventromedial, and (D) ventral views, showing the ventromedial concavity. *Paludirex* cf. *P. vincenti*, PVM 89-1072, photographs of the left jugal in (E) oblique ventromedial, and (F) ventral views, showing the ventromedial concavity. The scale bar in (D) is 50 mm. Abbreviations: dis alw, damaged distal alveolar wall; ect asp, ectopterygoid ascending process; jug asp, jugal ascending process; jug mf, medial jugal foramen; jug om, jugal orbital margin; jug vmcon, jugal ventromedial concavity; max, maxilla; max alv, maxillary alveolus; max t, maxillary tooth; mes alw, damaged mesial alveolar wall.

**Figure 15:**
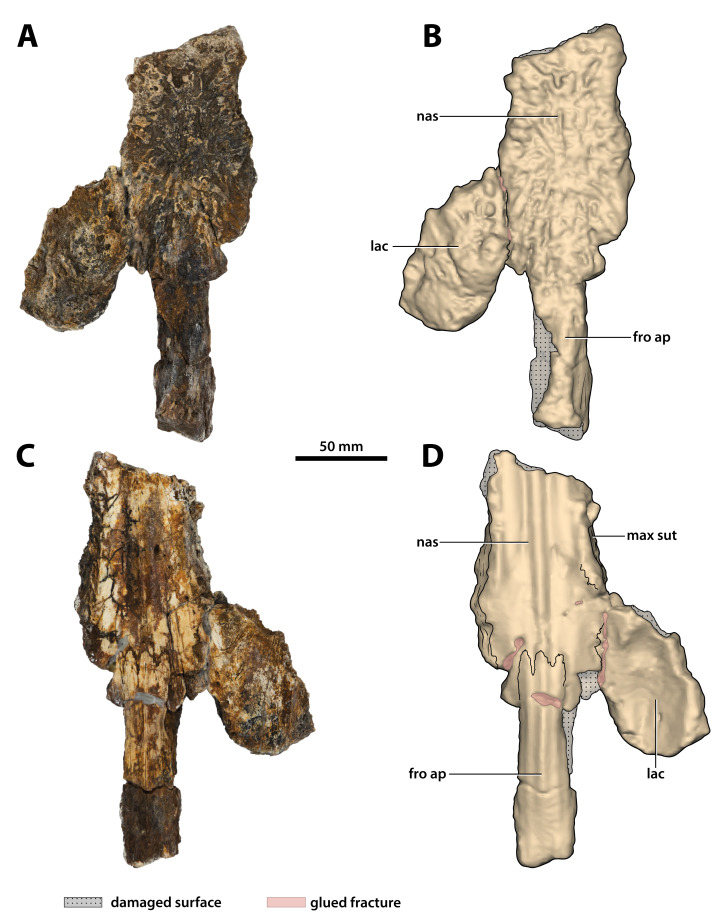
*Paludirex vincenti* gen. et sp. nov., ‘Geoff Vincent’s specimen’ (CMC2019-010-3), holotype, partial nasals, left lacrimal and anterior frontal process. (A) Photograph, and (B) annotated digital model in dorsal views. (C) Photograph, and (D) annotated digital model in ventral views. Abbreviations: fro ap, frontal anterior process; lac, lacrimal; max sut, sutural surface on nasal for articulation with the maxilla; nas, nasals.

**Figure 16:**
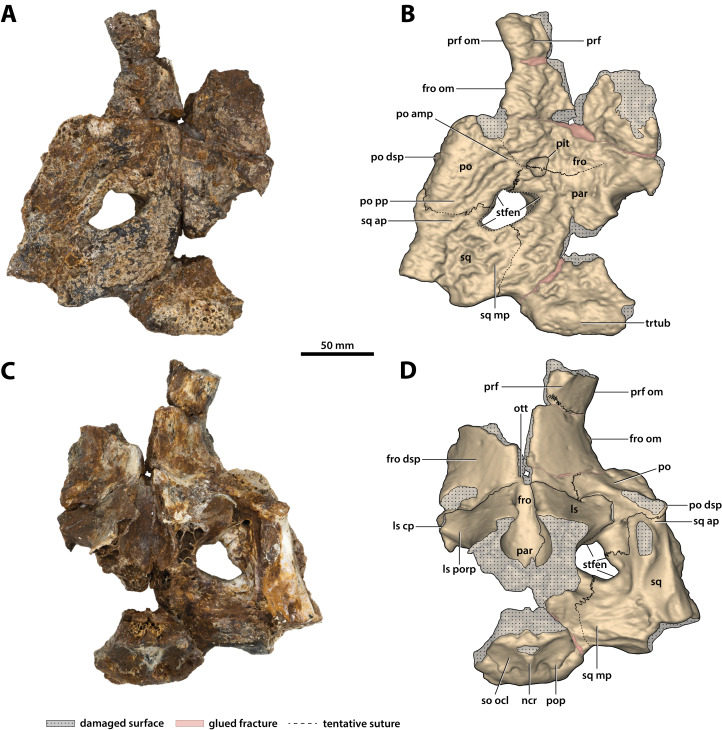
*Paludirex vincenti* gen. et sp. nov., ‘Geoff Vincent’s specimen’ (CMC2019-010-5), holotype, cranial table unit. (A) Photograph, and (B) annotated digital model in dorsal views. (C) Photograph, and (D) annotated digital model in ventral views. Abbreviations: fro, frontal; fro dsp, frontal descending process; fro om, frontal orbital margin; ls, laterosphenoid; ls cp, laterosphenoid capitate process; ls porp, laterosphenoid postorbital process; ncr, nuchal crest; ott, olfactory tract trough; par, parietal; po, postorbital; po amp, postorbital anteromedial process; po dsp, postorbital descending process; po pp, postorbital posterior process; pop, postoccipital process of the supraoccipital; prf, prefrontal; prf om, prefrontal orbital margin; so ocl, supraoccipital occipital lamina; sq, squamosal; sq ap, squamosal anterior process; sq mp, squamosal medial process; stfen, supratemporal fenestra; trtub, transverse tuberosity.

**Figure 17:**
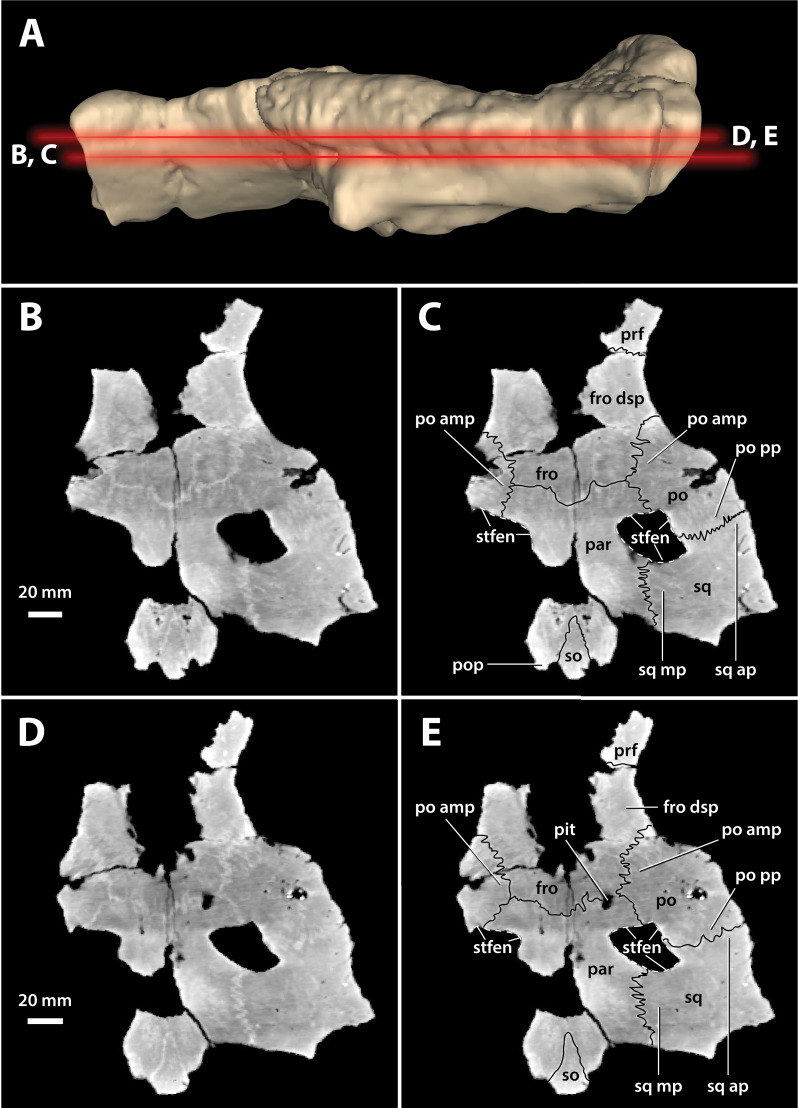
*Paludirex vincenti* gen. et sp. nov., ‘Geoff Vincent’s specimen’ (CMC2019-010-5), holotype, cranial table unit. (A) Digital model of the cranial table in left lateral view, with the red lines indicating the levels where the screenshots of the axial slices in B–E were taken. Non-annotated axial slices of CMC2019-010-5 in (B) and (D), and annotated axial slices of CMC2019-010-5 in (C) and (E). Abbreviations: fro, frontal; fro dsp, frontal descending process; par, parietal; po, postorbital; po amp, postorbital anteromedial process; po pp, postorbital posterior process; pop, postoccipital process of the supraoccipital; prf, prefrontal; so, supraoccipital; sq, squamosal; sq ap, squamosal anterior process; sq mp, squamosal medial process; stfen, supratemporal fenestra.

**Figure 18:**
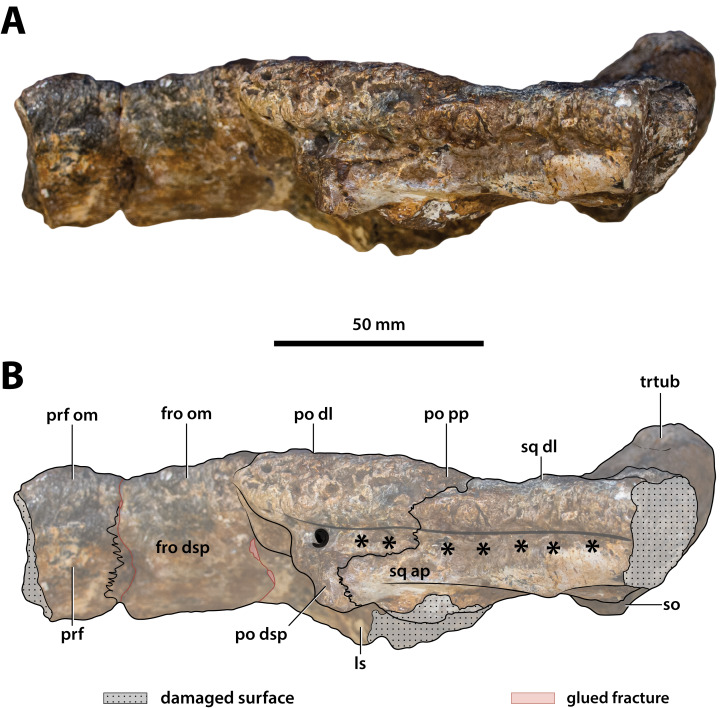
*Paludirex vincenti* gen. et sp. nov., ‘Geoff Vincent’s specimen’ (CMC2019-010-5), holotype, cranial table unit in left lateral view. (A) Non-annotated photograph, and (B) annotated photograph. The asterisks indicate the sulcus for attachment of the upper earlid musculature. Abbreviations: fro dsp, frontal descending process; fro om, frontal orbital margin; ls, laterosphenoid; po dl, postorbital dorsal lamina; po dsp, postorbital descending process; po pp, postorbital posterior process; prf, prefrontal; prf om, prefrontal orbital margin; so, supraoccipital; sq ap, squamosal anterior process; sq dl, squamosal dorsal lamina; trtub, transverse tuberosity.

**Figure 19:**
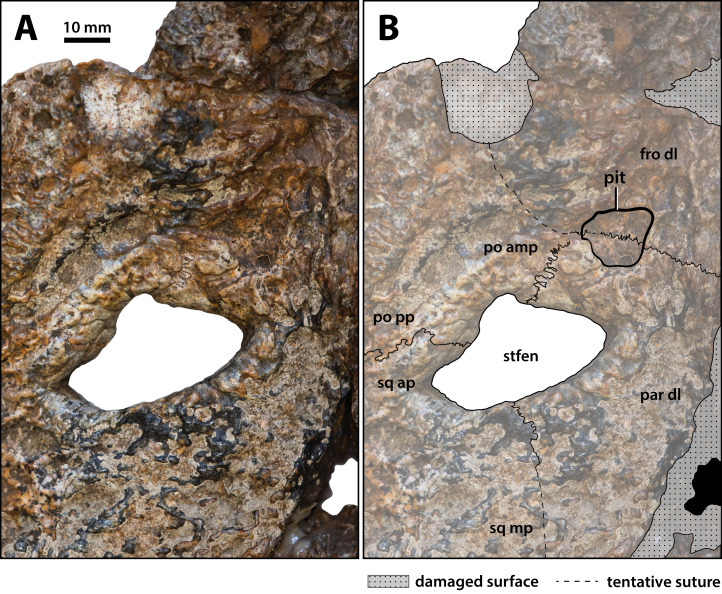
*Paludirex vincenti* gen. et sp. nov., ‘Geoff Vincent’s specimen’ (CMC2019-010-5), holotype, close up of the left supratemporal fenestra and surrounding region on the cranial table unit in dorsal view. (A) Non-annotated photograph, and (B) annotated photograph. Abbreviations: fro dl, frontal dorsal lamina; par dl, parietal dorsal lamina; po amp, postorbital anteromedial process; po pp, postorbital posterior process; sq ap, squamosal anterior process; sq mp, squamosal medial process; stfen, supratemporal fenestra.

**Figure 20:**
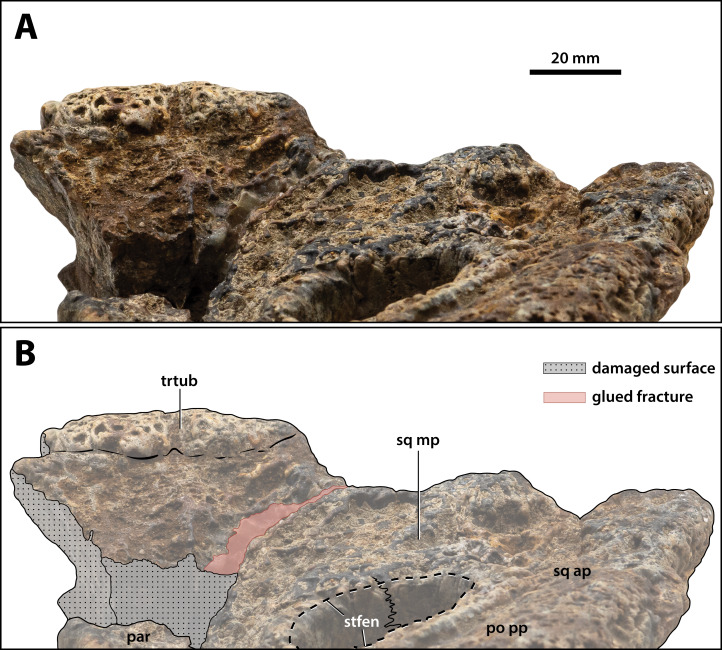
*Paludirex vincenti* gen. et sp. nov., ‘Geoff Vincent’s specimen’ (CMC2019-010-5), holotype, close up of the posterior portion of the cranial table unit in anterodorsal view. (A) Non-annotated photograph, and (B) annotated photograph. Abbreviations: par, parietal; po pp, postorbital posterior process; sq ap, squamosal anterior process; sq mp, squamosal medial process; stfen, supratemporal fenestra; trtub, transverse tuberosity.

**Figure 21:**
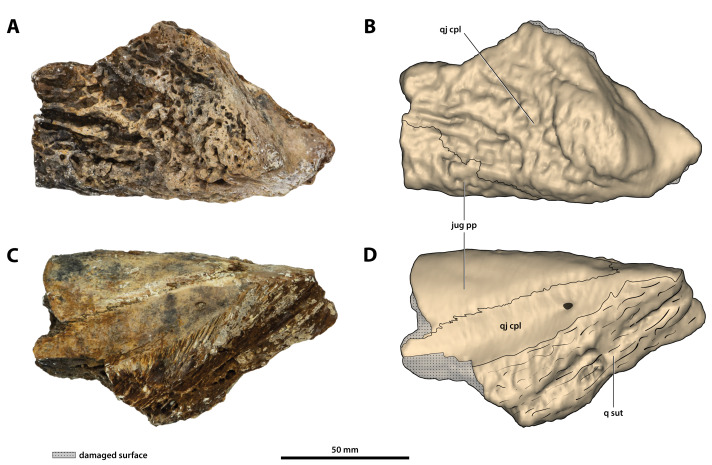
*Paludirex vincenti* gen. et sp. nov., ‘Geoff Vincent’s specimen’ (CMC2019-010-7), holotype, partial left quadratojugal and posterior process of the jugal. (A) Photograph, and (B) annotated digital model in dorsolateral views. (C) Photograph, and (D) annotated digital model in ventromedial views. Abbreviations: jug pp, jugal posterior process; q sut, sutural surface on quadratojugal for articulation with the quadrate; qj cpl, quadratojugal central plate.

**Figure 22:**
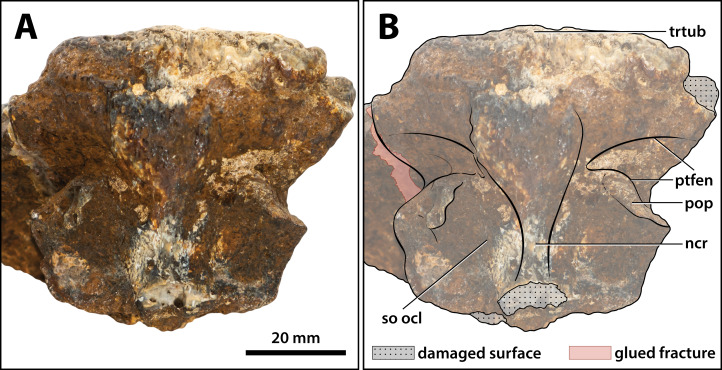
*Paludirex vincenti* gen. et sp. nov., ‘Geoff Vincent’s specimen’ (CMC2019-010-5), holotype, close up of the supraoccipital in occipital view. (A) Non-annotated photograph, and (B) annotated photograph. Abbreviations: ncr, nuchal crest; pop, postoccipital process of the supraoccipital; ptfen, posttemporal fenestra; so ocl, supraoccipital occipital lamina; trtub, transverse tuberosity.

**Figure 23:**
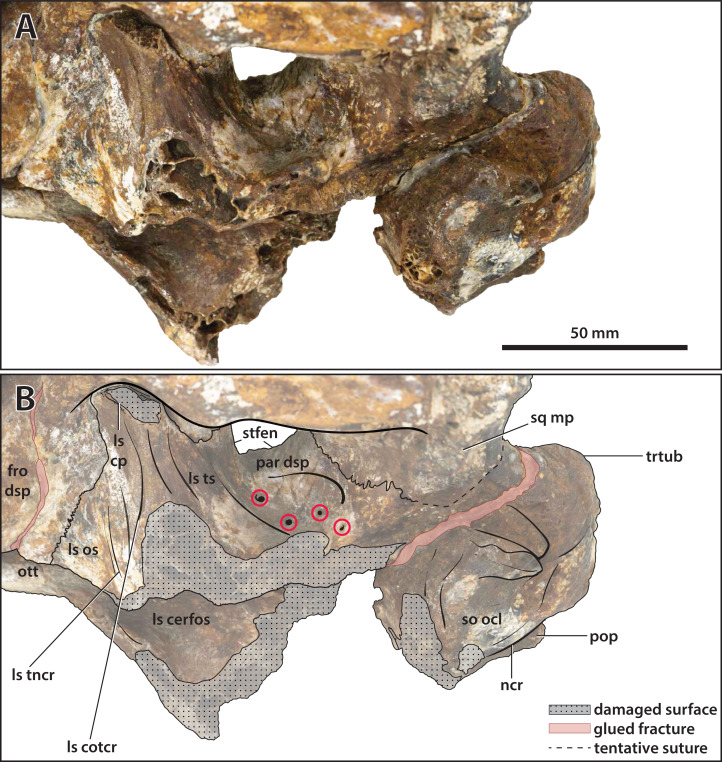
*Paludirex vincenti* gen. et sp. nov., ‘Geoff Vincent’s specimen’ (CMC2019-010-5), holotype, close up of the orbitotemporal region in left lateroventral view. (A) Non-annotated photograph, and (B) annotated photograph. The four red circles in (B) indicate the foramina perforating the descending process of the parietal. Abbreviations: fro dsp, frontal descending process; ls cerfos, laterosphenoid cerebral fossa; ls cotcr, laterosphenoid cotylar crest; ls cp, laterosphenoid capitate process; ls os, laterosphenoid orbital surface; ls tncr, laterosphenoid tensor crest; ls ts, laterosphenoid temporal surface; ncr, nuchal crest; ott, olfactory tract trough; par dsp, parietal descending process; pop, postoccipital process of the supraoccipital; so ocl, supraoccipital occipital lamina; sq mp, squamosal medial process; stfen, supratemporal fenestra; trtub, transverse tuberosity.

**Figure 24:**
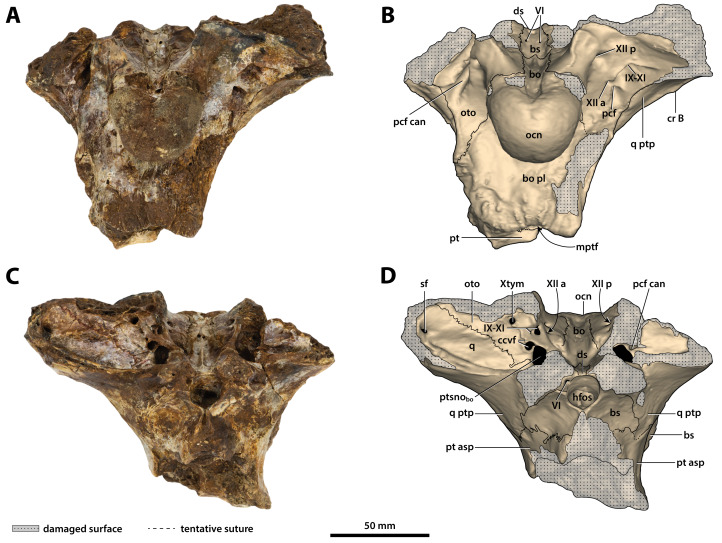
*Paludirex vincenti* gen. et sp. nov., ‘Geoff Vincent’s specimen’ (QMF59017), holotype, basicranium. (A) Photograph, and (B) annotated digital model in posterior views. (C) Photograph, and (D) annotated digital model in anterior views. Abbreviations: bo, basioccipital; bo pl, basioccipital plate; bs, basisphenoid; ccvf, cerebral carotid vasculature foramina; cr B, crest B of quadrate; ds, dorsum sellae; hfos, hypophyseal fossa; IX-XI, foramina for glossopharyngeal, vagus, and accessory nerves (metotic foramen); mptf, medial pharyngeal tube foramen; ocn, occipital condyle; oto, otoccipital; pcf, posterior carotid foramen; pcf can, canal of posterior carotid foramen (damaged); pt, pterygoid; pt asp, pterygoid ascending process; ptsno_bo_, communicating ostium between pharyngotympanic sinus and basioccipital diverticulum; q, quadrate; q ptp, quadrate pterygoid process; sf, siphonial tube foramen; VI, abducens foramina; Xtym, foramen for tympanic branch of glossopharyngeal and vagus nerves; XII a, anterior hypoglossal foramen; XII p, posterior hypoglossal foramen.

**Figure 25:**
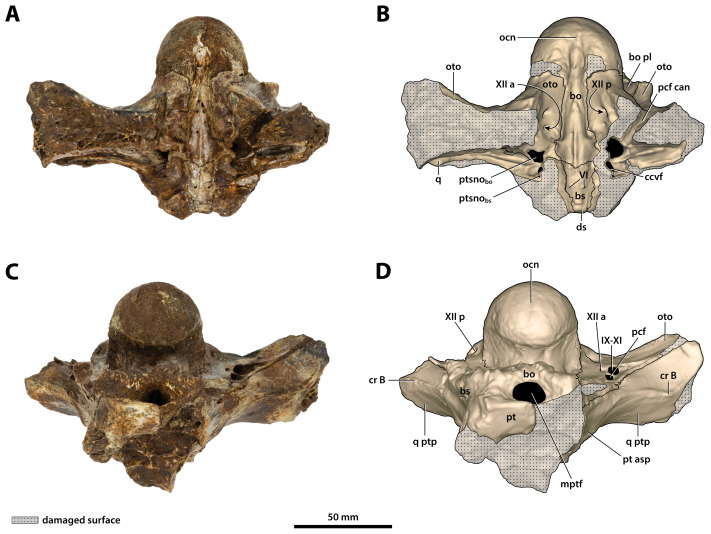
*Paludirex vincenti* gen. et sp. nov., ‘Geoff Vincent’s specimen’ (QMF59017), holotype, basicranium. (A) Photograph, and (B) annotated digital model in dorsal views. (C) Photograph, and (D) annotated digital model in ventral views. Abbreviations: bo, basioccipital; bo pl, basioccipital plate; bs, basisphenoid; ccvf, cerebral carotid vasculature foramen; cr B, crest B of quadrate; ds, dorsum sellae; IX-XI, foramina for glossopharyngeal, vagus, and accessory nerves (metotic foramen) ; mptf, medial pharyngeal tube foramen; ocn, occipital condyle; oto, otoccipital; pcf, posterior carotid foramen; pcf can, canal of posterior carotid foramen (damaged); pt, pterygoid; pt asp, pterygoid ascending process; ptsno_bo_, communicating ostium between pharyngotympanic sinus and basioccipital diverticulum; ptsno_bs_, communicating ostium between pharyngotympanic sinus and basisphenoid diverticulum; q, quadrate; q ptp, quadrate pterygoid process; VI, abducens foramina; XII a, anterior hypoglossal foramen; XII p, posterior hypoglossal foramen.

**Figure 26:**
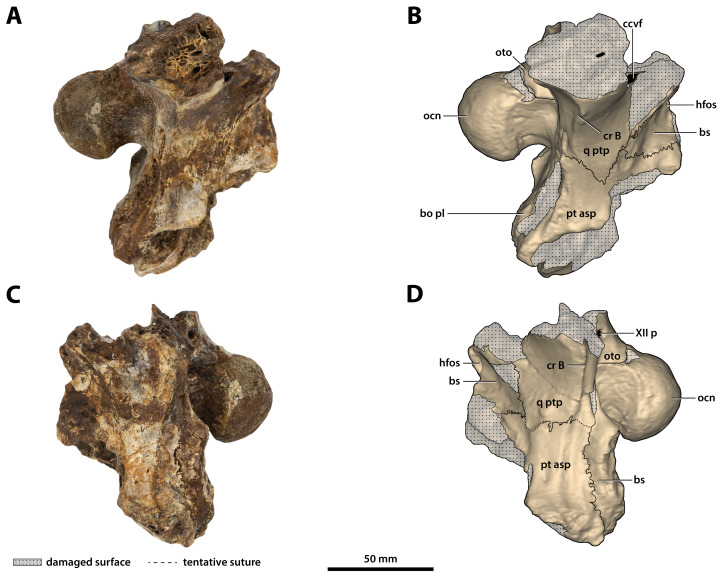
*Paludirex vincenti* gen. et sp. nov., ‘Geoff Vincent’s specimen’ (QMF59017), holotype, basicranium. (A) Photograph, and (B) annotated digital model in right lateral views. (C) Photograph, and (D) annotated digital model in left lateral views. Abbreviations: bo pl, basioccipital plate; bs, basisphenoid; ccvf, cerebral carotid vasculature foramen; cr B, crest B of quadrate; hfos, hypophyseal fossa; ocn, occipital condyle; oto, otoccipital; pt asp, pterygoid ascending process; q ptp, quadrate pterygoid process; XII p, posterior hypoglossal foramen.

**Figure 27:**
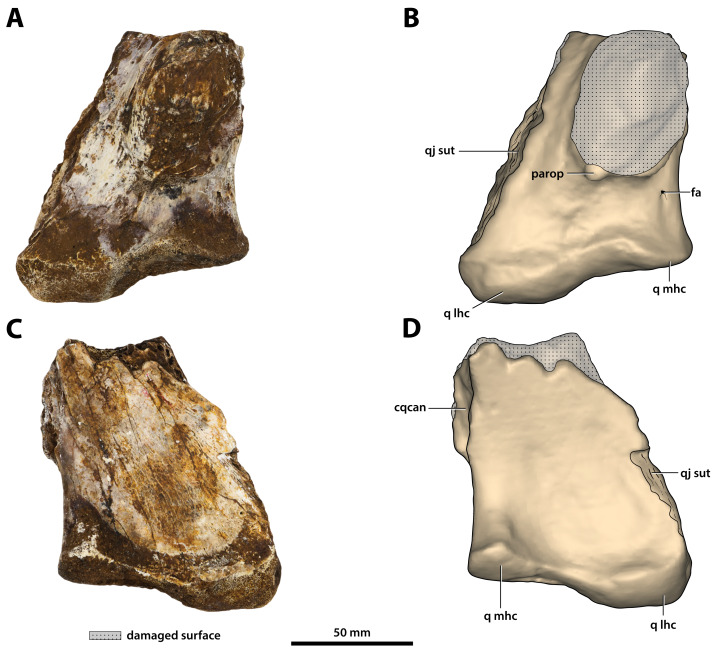
*Paludirex vincenti* gen. et sp. nov., ‘Geoff Vincent’s specimen’ (CMC2019-010-6), holotype, left quadrate body. (A) Photograph, and (B) annotated digital model in dorsal views. (C) Photograph, and (D) annotated digital model in ventral views. Abbreviations: fa, foramen aëreum; cqcan, cranioquadrate canal (lateral wall); parop, paroccipital process (tip); q lhc, quadrate lateral hemicondyle; q mhc, quadrate medial hemicondyle; qj sut, sutural surface on quadrate for articulation with the quadratojugal.

**Figure 28:**
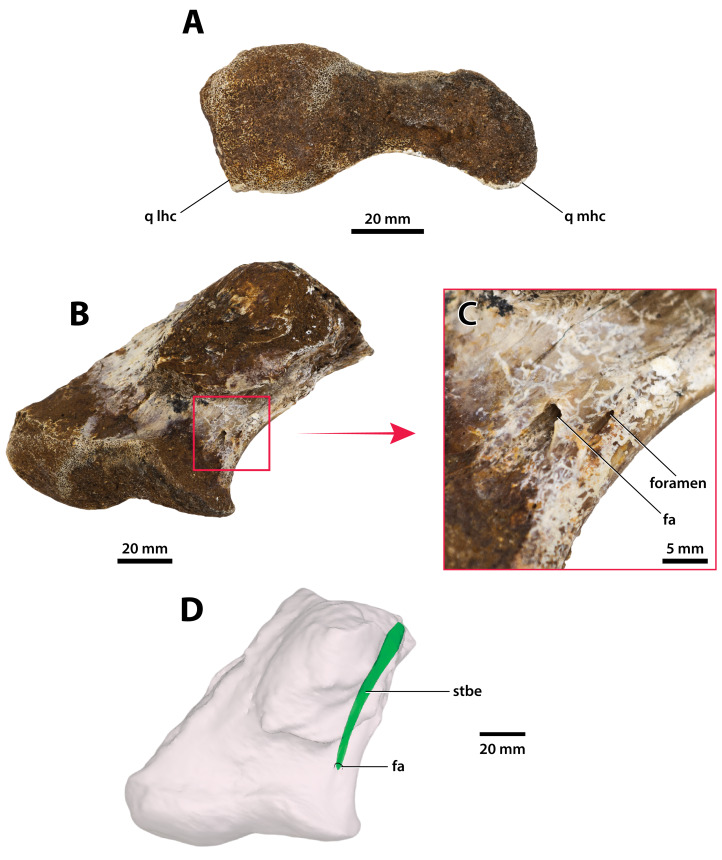
*Paludirex vincenti* gen. et sp. nov., ‘Geoff Vincent’s specimen’ (CMC2019-010-6), holotype, left quadrate body. (A) Photograph of the condylar surface. (B) Left quadrate body in oblique dorsomedial view, with the red square highlighting the portion in (C), which is a close up of the foramen aëreum and its surrounding region. (D) Transparent digital model of the left quadrate body in dorsal view, exposing the digitally segmented siphonial tube. Abbreviations: fa, foramen aëreum; q lhc, quadrate lateral hemicondyle; q mhc, quadrate medial hemicondyle; stbe, siphonial tube.

**Figure 29:**
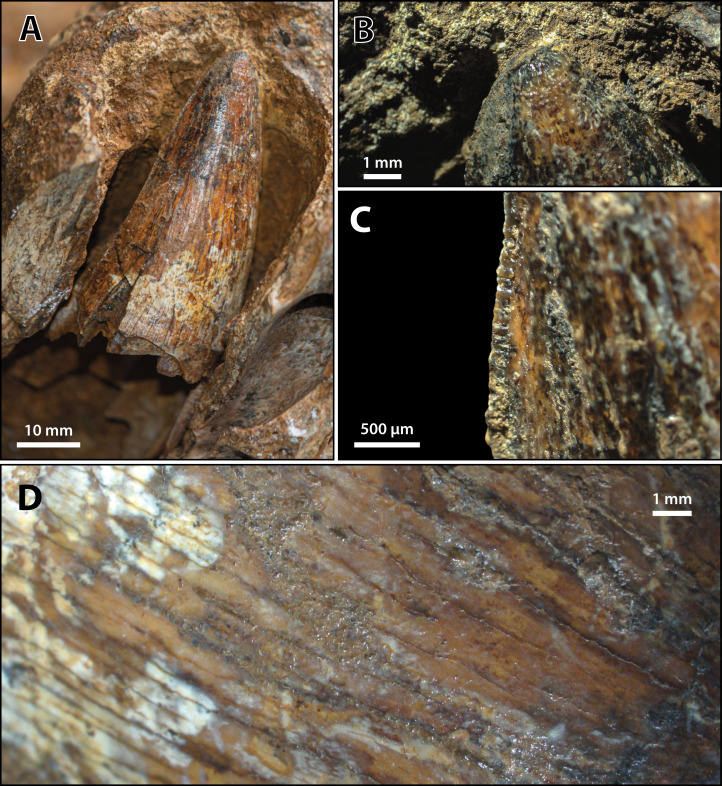
*Paludirex vincenti* gen. et sp. nov., ‘Geoff Vincent’s specimen’ (CMC2019-010-2), holotype, 5th maxillary tooth from the left maxilla. (A) Photograph of the tooth crown in labial view. (B) Close up of the crown apex, showing the anastomosed enamel texture around the apical region. (C) Close up on the distal carina in labial view. (D) Enamel texture on the labial surface.

**Figure 30:**
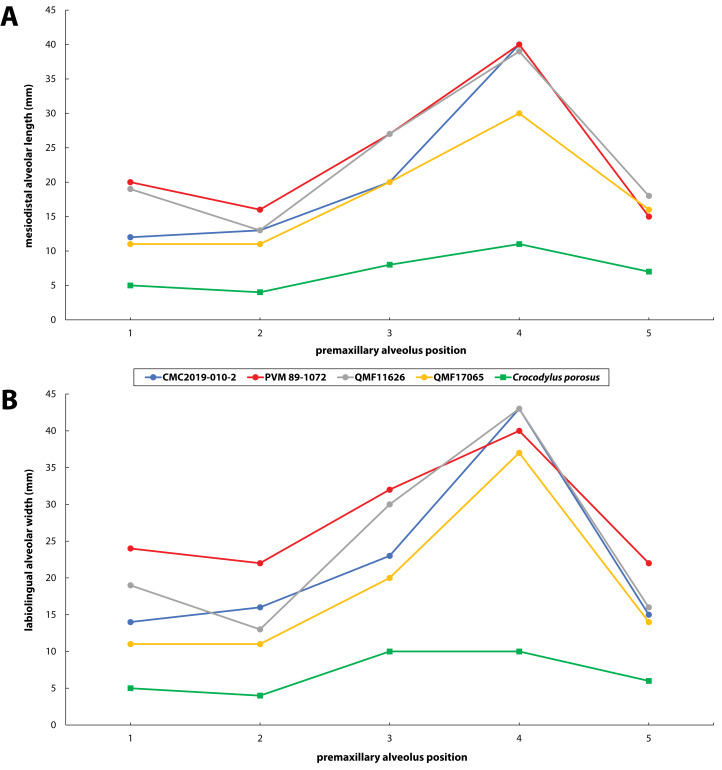
Premaxillary alveoli size comparisons between four *Paludirex* gen. nov. specimens and *Crocodylus porosus* Schneider, 1801. Graphs showing the (A) mesiodistal length, and (B) the labiolingual width of the premaxillary alveoli. Notice how the *Paludirex* specimens have a greater size disparity between the premaxillary alveoli, especially the 3rd and 4th, when compared to the *Crocodylus porosus* specimen (UQSSAL J7). Measurements of the premaxillary alveoli for UQSSAL J7 can be found in the **Table S2.2**.

**Figure 31:**
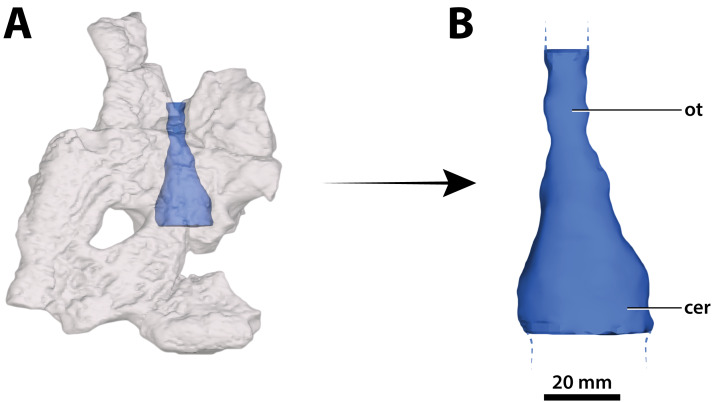
*Paludirex vincenti* gen. et sp. nov., ‘Geoff Vincent’s specimen’ (CMC2019-010-5), holotype, part of the brain endocast. (A) Transparent digital model of the cranial table in dorsal view, exposing the digitally segmented brain endocast. (B) The partial digital brain endocast (corresponding with the telencephalic region) in dorsal view. Abbreviations: cer, cerebrum (endocast); ot, olfactory tract (endocast).

**Figure 32:**
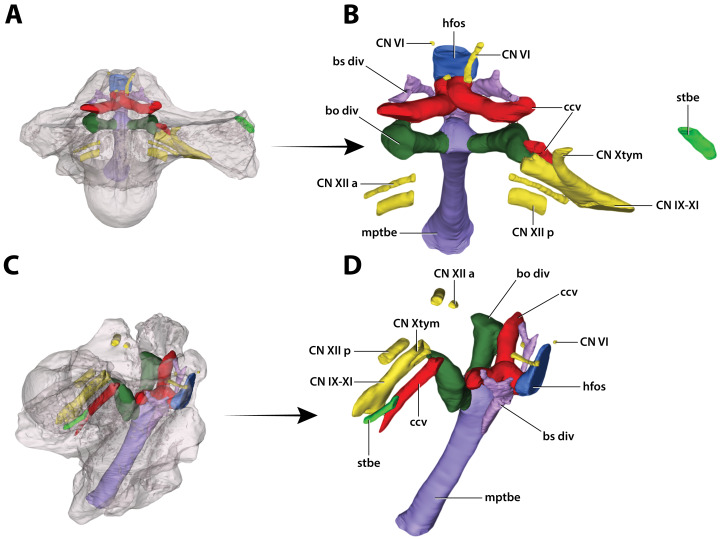
*Paludirex vincenti* gen. et sp. nov., ‘Geoff Vincent’s specimen’ (QMF59017), holotype, digitally segmented basicranial elements. (A) Transparent digital model of the basicranium in dorsal view, exposing the digitally segmented basicranial elements as detailed in (B). (C) Transparent digital model of the basicranium in oblique right lateral view, exposing the digitally segmented basicranial elements as detailed in (D). Abbreviations: bo div, basioccipital diverticulum; bs div, basisphenoid diverticulum; ccv, cerebral carotid vasculature canal; CN IX-XI, shared canal for the glossopharyngeal, vagus, and accessory nerves and accompanying vessels; CN VI, abducens nerve canal; CN Xtym, canal for the tympanic branch of glossopharyngeal and vagus nerves; CN XII a, anterior hypoglossal canal; CN XII p, posterior hypoglossal canal; hfos, hypophyseal fossa endocast; mptbe, median pharyngeal tube; stbe, siphonial tube.

**Figure 33:**
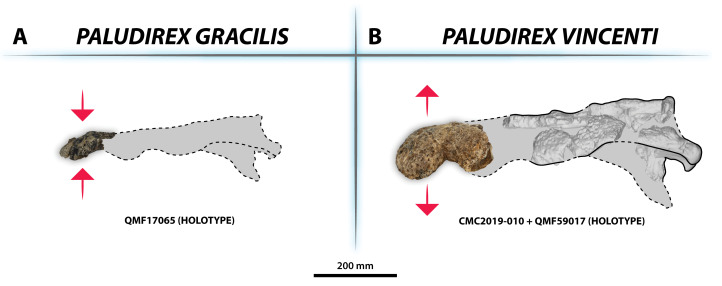
Interspecific skull and rostrum robustness variation within *Paludirex* gen. nov. (A) *Paludirex gracilis* gen. et comb. nov. (B) *Paludirex vincenti* gen. et sp. nov. Notice the proportionally deeper rostrum of *Paludirex vincenti* as opposed to *Paludirex gracilis*. The dashed lines highlight the hypothetical outlines of the complete skulls (based on ‘Geoff Vincent’s specimen’). The photograph of the right premaxilla QMF17065 has been reflected in order to aid the comparison.

ZooBank LSID for the genus: [urn:lsid:zoobank.org:act:62FE01E1-BC2C-4EC0-8022-0DE6DE9A1A88].

**Type species**—*Paludirex vincenti* gen. et sp. nov.

**Etymology**—The generic name is from the Latin *paludis* and *rex*, meaning ‘swamp king’. This roughly preserves the original meaning of *Pallimnarchus* which is a Greek contraction with the meaning ‘ruler of all the swamps’.

**Generic diagnosis**—Large-bodied mekosuchine crocodyloids (capable of attaining a TL of at least 4 m at maturity) with proportionately broad snouts characterized by the following unique combination of features: (**1**) dorsally arched premaxillary alveolar processes at the level of the first two alveoli; (**2**) anterior profile of the premaxilla in lateral view is shallowly sloping; (**3**) premaxillary alveoli with circular to sub-circular outlines; (**4**) 1st and 2nd premaxillary alveoli approximately in line with each other, and separated by substantial interalveolar spaces more so than the other premaxillary alveoli; (**5**) large 4th premaxillary alveolus with a circumference 1.5 to 2 times greater than the 3rd premaxillary alveolus.

*PALUDIREX VINCENTI* GEN. ET SP. NOV.

ZooBank LSID for the species: [urn:lsid:zoobank.org:act:BF91D49B-642D-404A-BA31-13D53240D056].

**Etymology**—The specific epithet honors the late Mr. Geoff Vincent, discoverer of the holotype specimen.

**Holotype**—‘Geoff Vincent’s specimen’ (CMC2019-010 + QMF59017), partial skull.

**Type locality and horizon**—A property adjacent to the Chinchilla Riffle Range, Darling Downs, south-eastern Queensland; Chinchilla Sand deposits, Chinchilla Local Fauna, age Pliocene, Neogene.

**Referred specimen**—QMF11626, left premaxilla and partial maxilla, found along the Condamine River near Warra; age uncertain.

**Comments on the referred specimen**—QMF11626 (Fig. 3) is a complete left premaxilla that is still in articulation with the anterior portion of its left maxilla. Prior to this study, QMF11626 has been discussed and figured by Willis & Molnar (1997a) who referred it to *Pallimnarchus pollens* (see Fig. 1D in the aforementioned). Based on museum records, QMF11626 was found along the Condamine River, 12 km east of Warra (Fig. 2). Warra is a small town in the Darling Downs region in Queensland, and in its vicinity are outcrops of the Pliocene Chinchilla Sand (Louys & Price, 2015). However, the nearby area is also known to have produced Pleistocene fossils as well (Mackness & Godthelp, 2001). The only age information in the published literature for QMF11626 is in Lees (1986) who reported its age as Pliocene, although how that age was determined was not elaborated. Therefore, the age of QMF11626 is presently uncertain, but could be either Pliocene or Pleistocene.

**Specific diagnosis**—Large-bodied mekosuchine crocodyloids (capable of attaining over 4 m of TL at maturity) with proportionately broad snouts (maximum snout width is ~50% the skull length) characterized by the following unique combination of features (autapomorphies indicated with *): (**1**) ornamented portion of anterior process of the jugal oriented laterally; (**2**) ascending process of the jugal deeply inset from the lateral surface of jugal; (**3**) medial surface of jugal ventral to the ascending jugal process concave*; (**4**) a pair of sculpture pits that are larger than the surrounding cranial table ornamentation located anterior to the supratemporal fenestrae, spread over the frontoparietal sutural junction; (**5**) small supratemporal fenestrae (occupying less than 10% of cranial table surface) with approximately D-shaped dorsal outlines; (**6**) descending process of the parietal perforated by conspicuous foramina*; (**7**) posteromedial portion of cranial table with a gentle anterior inclination, bearing a thickened transverse tuberosity and protruding far posteriorly relative to the medial process of the squamosal*; (**8**) occipital lamina of the supraoccipital oriented posteroventrally*; (**9**) postoccipital processes of supraoccipital relatively small; (**10**) occipital lamina of supraoccipital bears a nuchal crest that is not laterally delimited by concavities; (**11**) pterygoid process of the quadrate with evident occipital exposure ventrolateral to otoccipital; (**12**) significant ventral process of the quadrate on lateral braincase wall; (**13**) dentary teeth occlude lingual to maxillary teeth, except for the 4th dentary tooth which fits in a deep notch at the premaxillomaxillary suture; (**14**) maxillary teeth/alveoli with high size-disparity, having the largest maxillary tooth/alveolus at least twice the diameter of the smallest interfestoonal tooth/alveolus; (**15**) all premaxillary and maxillary alveoli with circular to sub-circular outlines; (**16**) anterior maxillary alveoli with minimal to no interalveolar spaces between them; (**17**) teeth conidont, with large individuals having non-fluted enamel surfaces and crenulated carinae.

*PALUDIREX* CF. *P. VINCENTI*

**Referred specimen**—PVM 89-1072, partial skull from the Pioneer Valley area, Mackay region, Queensland; age unknown.

**Comments**—PVM 89-1072 (Fig. 4) is a partial and fractured skull. As the specimen has been embedded in a slab of concrete during preparation, only its ventral surface is fully exposed; small sections of the anterior, posterior and lateral parts of the skull that protrude out of the concrete are also observable. This specimen was first mentioned by Molnar (1991). The specimen was further discussed by Willis & Molnar (1997a) as the ‘Mirani Museum skull’ or ‘Mirani Shire skull’ and referred to *Pallimnarchus gracilis* (see Fig. 7 in Willis & Molnar, 1997a; Fig. 2.28 in Molnar, 2004; Fig. 11 in Willis, 2006). The specimen was originally part of the collection of John (‘Jack’) Henry Williams (also known as Jack Williams Jr.), purchased by the Mirani Museum (now the PVM) in 1986, and was added to the museum catalogue in 1989. The exact location of the skull’s discovery is unknown, with reports that it came from the top of Mt Robert, approximately 75 km southwest of Mackay, seemingly an error (see Willis & Molnar, 1997a). Jack Williams Jr., who lived in the township of Eungella, approximately 70 km west of Mackay, collected rocks and fossils throughout the Pioneer Valley and surrounding region (Steven W. Salisbury and D. Ruthenberg, 2020, personal communication). It is likely the skull came from one of the Pleistocene fluvial deposits that occur in this area, similar to those a little farther to the south at South Walker Creek, near Nebo, which were recently described by Hocknull et al. (2020).

*PALUDIREX GRACILIS* (Willis & Molnar, 1997a) GEN. ET COMB. NOV.

*Pallimnarchus gracilis* Willis & Molnar, 1997a, pp. 223–240, figs. 1A–1C, 2, 5A (original published description)

*Pallimnarchus gracilis* Willis & Molnar, 1997a; Molnar, 2004, pp. 57–58

*Pallimnarchus gracilis* Willis & Molnar, 1997a; Archer et al., 2006, p. 9

*Pallimnarchus gracilis* Willis & Molnar, 1997a; Willis, 2006, pp. 340–341

*Pallimnarchus gracilis* Willis & Molnar, 1997a; Webb, 2010, p. 319

*Pallimnarchus gracilis* Willis & Molnar, 1997a; Sobbe, Price & Knezour, 2013, p. 605

*Pallimnarchus gracilis* Willis & Molnar, 1997a; Webb, 2013, p. 160

*Pallimnarchus gracilis* Willis & Molnar, 1997a; Cook & Rozefelds, 2014, p. 243

‘*Pallimnarchus*’ *gracilis* Willis & Molnar, 1997a; Yates, 2017, p. 33

‘*Pallimnarchus*’ *gracilis* Willis & Molnar, 1997a; Lee & Yates, 2018, figs. 1, 2 and 4, Appendix 1, figs. S1–S8

*Pallimnarchus gracilis* Willis & Molnar, 1997a; Hocknull et al., 2020, pp. 9–11 in Supplementary Information

ZooBank LSID for the species: [urn:lsid:zoobank.org:act:457C24F2-948E-4477-AD42-5504A516FA85].

**Holotype**—QMF17065, right premaxilla.

**Type locality and horizon**—Terrace Site, Riversleigh, late Pleistocene, Quaternary.

**Comments on the holotype specimen**—QMF17065 (Fig. 5) is a virtually complete, yet toothless right premaxilla. This specimen was described in detail by Willis & Molnar (1997a; also, Willis, 1995) and designated as the holotype of *Pallimnarchus gracilis* (see Fig. 13.1 in Willis, 1995; Figs. 1A–C and 5A in Willis & Molnar, 1997a). QMF17065 was recovered from the Terrace Site at Riversleigh, Queensland (Fig. 2), a deposit that is thought to be late Pleistocene in age (Willis & Molnar, 1997a; Archer, Hand & Godthelp, 2000, Archer et al., 2006; Woodhead et al., 2016).

**Referred specimen**—QMF17066, anterior fragment of a left dentary recovered in association with QMF17065. From Terrace Site at Riversleigh, age late Pleistocene, Quaternary.

**Comments on the referred specimen**—QMF17066 (Fig. 6) is a poorly preserved anterior fragment of a left dentary that, according to Willis & Molnar (1997a) was recovered in association with QMF17065, and hence assigned to *Pallimnarchus gracilis* by the same (see Fig. 2A–D in Willis & Molnar, 1997a).

**Specific diagnosis**—Broad-snouted species of *Paludirex* characterized by the following unique combination of features (autapomorphy indicated with *): (**1**) large 4th premaxillary alveolus with a circumference 1.5 times greater than the 3rd premaxillary alveolus; (**2**) proportionally less robust and with a dorsoventrally shallower cranial rostrum than P. vincenti*.

**The ‘Geoff Vincent specimen’ (CMC2019-010 + QMF59017)**

The so-called ‘Geoff Vincent’s specimen’ (Fig. 7) was first mentioned in the publication by Willis & Molnar (1997a), but hitherto barely described and never figured. The reason why the ‘Geoff Vincent’s specimen’ was chosen as the centerpiece for this study’s morphological assessment is because it is by far the best preserved and most complete *Paludirex* specimen. Indeed, it is also one of the better preserved and arguably most complete when compared to other material that was referred to *Pallimnarchus* in past studies. Even though ‘Geoff Vincent’s specimen’ is fractured, its remains provide important morphological information that aid with comparative anatomy and inclusion in cladistic analyses. Where appropriate, the description is augmented with data from the other *Paludirex* specimens. Accompanying the detailed morphological description is an interactive 3D PDF document containing a digital model of ‘Geoff Vincent’s specimen’, serving as a supplementary file to this paper.

**Specimen background**

The holotype of *P. vincenti*—‘Geoff Vincent’s specimen’ (CMC2019-010 + QMF59017; also referred to as ‘the Dalby specimen’ by Willis, 1995)—was discovered by the late Mr. Geoff Vincent, a former resident of the town Dalby in south-eastern Queensland, sometime between the years 1984 and 1990 near the town of Chinchilla, southeast Queensland (Figs. 2, 8A and 8B). All individual pieces of the skull were found in association and also match in preservation and proportions (Willis, 1995; Willis & Molnar, 1997a). Because the exact locality of discovery was not disclosed in the initial publication that reported this specimen (Willis & Molnar, 1997a), it was uncertain whether it is Pliocene (typical of the western Darling Downs) or Pleistocene (typical of the eastern Darling Downs) in age. Personal correspondence between Ralph E. Molnar and Mr. Vincent revealed that the holotype was recovered on a property adjacent to the Chinchilla Rifle Range, which indicates it derives from the western Darling Downs. That means the specimen is from the Chinchilla Sand, a Pliocene fluviatile deposit rich in vertebrate fossils (i.e., the Chinchilla Local Fauna; Price, 2012; see Louys & Price, 2015 for a review; also referred as the western Downs fauna, Molnar & Kurz, 1997). This is further supported by the lithology and preservational state of the specimen, which conforms with other material from the same deposit. Crocodylian fossils from the Chinchilla Sand formerly referred to *Pallimnarchus* have been reported on multiple occasions (Hill, Playford & Woods, 1970; Bartholomai & Woods, 1976; Gorter & Nicoll, 1978; Molnar, 1982b; Vickers-Rich & Rich, 1999; Willis & Molnar, 1997a, 1997b; Mackness et al., 2010; Louys & Price, 2015; Chiotakis, 2018, 2019). Other than *Paludirex*, ziphodont crocodylian remains are also components of the Chinchilla Local Fauna (Molnar, 1981; Mackness et al., 2010; Louys & Price, 2015; Chiotakis, 2018, 2019).

Subsequent to its discovery, Mr. Vincent loaned the specimen to the Queensland Museum for research where it was also put on display (first in 1990) for several years (Fig. 8C), before it was placed in storage. Eventually, in 2008 the specimen was returned to Mr. Vincent’s wife, the late Mrs. Dot Vincent, after which it was donated to the Chinchilla Museum in 2011 following Mr. Vincent’s wish to have the crocodylian fossil exhibited there. However, out of the eight associated skull pieces only seven were returned to Chinchilla, with the basicranial unit being left behind at the Queensland Museum in Brisbane. In the meantime, the basicranium remained an undetermined and unregistered fossil at the Queensland Museum until it was identified as part of the ‘Geoff Vincent specimen’ by Jorgo Ristevski in 2018. Hence, the basicranium has a different registry (QMF59017) from its other related elements (CMC2019-010). Prior to this study, the specimen was only briefly discussed by Willis (1995) and Willis & Molnar (1997a) where it was assigned to *Pallimnarchus pollens*. Out of convenience, the appellation ‘Geoff Vincent’s specimen’ given by the aforementioned publication will be used throughout this paper.

**Description**

**Major cranial fenestrae and foramina**

**External narial fenestra**

Opening dorsomedially on the rostral piece (CMC2019-010-2) is the external narial fenestra (Figs. 9, 10A, 10B, 11A and 11B). The external narial fenestra is complete, except for its broken posteromedial rims. The fenestra is encompassed almost exclusively by the premaxillae, with the exception of its posteromedial narial margin which likely received a modest contribution by the nasals (character 82, state 1). Its overall outline is circular (character 83, state 0), with an anteroposterior diameter of ~67 mm and ~66 mm in transverse diameter. The external narial fenestra is oriented almost fully dorsally with a very mild anterior inclination (character 81, state 1). The fenestra is not situated too close to the anterior edges of the premaxillae, with the distance from the anterior-most margins of the premaxillae to the anterior-most margin of the external narial fenestra being ~51 mm. None of the circumscribing premaxillary rims overhang the fenestra itself, with the lateral margins of the external narial fenestra not being noticeably more rugose from the circumambient external premaxillary surfaces. The rims surrounding the fenestra seem devoid of any crests or excrescences (character 85, state 0).

**Incisive foramen**

The incisive foramen is the aperture opening centrally on the premaxillary palatal processes (Figs. 9A, 9B, 10A and 10B). It is fairly large, with its anterior margin being clearly visible from a dorsal aspect when viewed through the external narial fenestra. Unfortunately, most of its rims are damaged and largely missing, thus hindering assessment of its exact shape and size. The incisive foramen has a semicircular anterior margin that as preserved attains a transverse width of ~41 mm. The anterior margin of the incisive foramen is nearly at the same anterior level as the anterior margin of the external narial fenestra, only slightly more posterior. Furthermore, the anterior margin of the foramen is not close to the lingual alveolar margins, and is on the same plane as the 4th premaxillary alveoli (character 89, state 0). The anterior edge of the foramen is only a short distance from the posterior margins of the reception pits for the 1st dentary teeth.

**Supratemporal fenestrae**

The supratemporal fenestra (=dorsotemporal or upper temporal fenestra; supratemporal foramen of Salisbury et al., 2003) on the left side is complete, whereas the right fenestra preserves only its anterior and anteromedial margins (Figs. 16, 17, 19, 20 and 23); hence, this description is based entirely on the left fenestra. The supratemporal fenestra is very small relative to the cranial table (~27 mm in length as measured along anterior margin; ~44 mm in length as measured along medial margin; ~32 mm in length as measured along lateral margin) and irregularly shaped, appearing in the form of the letter D (character 207, state 5). The shape of the supratemporal fenestra as seen in the *P. vincenti* holotype is unique, although somewhat resembling that of *Baru wickeni* Willis, 1997 NTM P91171-1 (Figs. S1.14F and S1.14H; see also Yates, 2017). The anterior margin of the fenestra is oriented transversely, the lateral fenestral margin is sub-parallel to the lateral margin of the supratemporal arch, while the medial margin of the fenestra is oriented diagonally, resulting with a medially angled anteromedial margin. Due to the peculiar shape of the fenestra, the medial and lateral margins merge smoothly at the posterior angle, which render a relatively indistinct posterior fenestral margin. None of the surrounding cranial table bones overhang the rims of the fenestra (character 152, state 0), and yet the supratemporal fossa is diminished. The frontoparietal fossa (sensu Holliday et al., 2020) has virtually no exposure dorsally on the cranial table (character 210, state 0; Fig. 19). Most of the anterior and medial margins of the fenestra are comprised by the left lateral margin of the parietal, with the parietal descending process (=crista cranii parietalis; parietal descending lamina of Sertich & O’Connor, 2014) forming the anterior and medial walls of the supratemporal fossa. The parietal-postorbital contact completely excludes the frontal from contributing to the fenestra (character 150, state 2). The postorbital comprises at least 50% of the lateral and a small part of the anterolateral margins, while the squamosal contributes to the posterolateral, posterior, and to a small degree the posteromedial margins of the supratemporal fenestra. The sutures are difficult to see on the dorsal cranial surface but are visible around the perimeter of the left fenestra and along the lateral walls of its rudimentary supratemporal fossa (however, the sutures of the cranial table can be viewed with greater clarity thanks to the CT scan data; Fig. 17).

**Foramen magnum**

The only remnants of the foramen magnum are its ventrolateral and ventral margins, allowing for a very limited description (Figs. 24A and 24B). The foramen is situated at the center of the occipital surface, having an arch-like ventral outline and a maximum preserved transverse width of ~35 mm. The lateral and ventrolateral margins are comprised of the otoccipitals, and its medioventral margin by the basioccipital.

**Dermatocranial bones**

**Premaxillae**

Both premaxillae are mostly complete and relatively well preserved (Figs. 9, 10A, 10B, 11A and 11B), although much of their external surfaces have a poorly preserved texture, which is partially obscuring their ornamentation. What is observable of the dorsal and lateral ornamentation hints at a grooves-and-ridges type of pattern, which is consistent with the other ornamented pieces of the same specimen (i.e., CMC2019-010-1, CMC2019-010-3, CMC2019-010-4, CMC2019-010-5 and CMC2019-010-7). The palatal processes of the premaxillae have sustained the most significant damage, missing most of their medial portions that surrounded the incisive foramen and formed the premaxillary section of the secondary bony palate (Figs. 10A and 10B). The ascending processes of the premaxillae (sensu Iordansky, 1973; =dorsolateral process sensu Kley et al., 2010) posterior to the external narial fenestra are also incomplete to a degree, as they are missing their posterior tips. In addition, there is a very thin crack across the dorsolateral surface on the left premaxilla, and some sections are covered in glue (anteromedially at the premaxillary symphysis and posterolaterally at the contact with the anterior maxillary pieces; some glue residue also remains posteromedially where the nasals used to be glued to the premaxillae) after they underwent restoration.

The premaxillary pair comprised the anterior of the rostrum (snout). They are robust, broad (~240 mm collective premaxillary width at their widest portion, which corresponds to the level of the fourth premaxillary alveoli) and laterally expanded. Their collective width is greater than their height, and thus indicative of both a brevirostrine (‘short’ or ‘blunt snouted’), platyrostral (‘flat snouted’) snout morphology. As preserved, the premaxillae contact the maxillae posterolaterally and the nasals posteromedially. The premaxillary-maxillary sutures are visible laterally and ventrally on both sides (Figs. 9C, 9D, 10A and 10B), however, the glue covering on the left ventral surface has obliterated the premaxillary-maxillary suture. In lateral views, the premaxillary-maxillary sutures ascend in a sub-vertical path, while ventrally the sutures are oriented posteromedially. The sutural surfaces between the premaxillae and nasals are also damaged, and the preserved anterior of the nasals are no longer in sutural articulation with the premaxillae, but were glued instead. The premaxillae are in contact with each other anteromedially, along the premaxillary symphysis; the premaxillary symphyseal plates are now glued together as well. Measured ventrally (from the labial rims of the first alveoli anteriorly, to the anterior rim of the incisive foramen posteriorly), the anteroposterior length of the premaxillary symphysis is ~52 mm. The premaxilla does not form an internarial process (sensu Iordansky, 1973; =ascending premaxillary process sensu Buckley et al., 2000; dorsomedial premaxillary process sensu Kley et al., 2010). The lateral surface on each side at the premaxillary-maxillary suture is concave because of a deep notch that accommodated a dentary tooth during jaw occlusion (character 195, state 0). These notches have sub-conical outlines, reflecting the shape of the mandibular tooth that lay within each notch. The notches give the posterolateral portions of the premaxillae (and to an extent the anterolateral portions of the maxillae) a markedly constricted appearance when viewed in dorsal and ventral aspects.

Large neurovascular foramina adorn the anterior laminae of the premaxillary alveolar processes, yet the foramina are also present further from the alveolar margins as well, up to approximately halfway on the premaxillae’s lateral and anterior external surfaces. The interforaminal spaces on the anterior laminae of the alveolar processes are smooth and unornamented, and alongside the large neurovascular are also tiny foramina spread over these surfaces. In anterior view, the alveolar processes of the premaxillae adopt an arch-like appearance anteromedially, due to their medially rising margins, starting around the mesiolabial rims of the 3rd alveoli laterally, and peaking at the level of the 1st alveoli medially (character 226, state 1; Fig. 11). The anteromedial margins of the premaxillae (corresponding to the level of the aforementioned arch-like peak) are straight in dorsal and ventral views, whereas the laterally expanded margins are rounded. Immediately anterior to the external narial fenestra, the external surfaces of the premaxillae begin to gently slope ventrally for a short distance (character 201, state 0), which is unlike the sub-vertical anterior premaxillary profiles of *Baru darrowi* Willis, Murray & Megirian, 1990 and *B. wickeni*. The labial alveolar rims are rugose.

Anteromedially on each premaxilla is a conspicuous perforation caused by the first dentary tooth (Figs. 11A and 11B). On the anterior laminae, the perforation on the right premaxilla has a highly elliptic shape (~16 mm dorsoventral height; ~8 mm transverse width), while the perforation on the left premaxilla is sub-circular and smaller than its right counterpart (~9 mm dorsoventral height; ~7 mm transverse width). The perforations are present on the posterior (lingual) laminae as very large and deep sub-circular reception pits, adapting the conical shapes carved by the now gone 1st dentary teeth that they received (Figs. 10A and 10B). The external (labial) rims around these perforations are rugose, as are their internal walls close to the labial surfaces. The nasal vestibulum, encased between the premaxillae, is relatively deep and sub-vertical (~48 mm dorsoventral height measured from the lateral rims of the external narial fenestra dorsally to the lateral rims of the incisive foramen ventrally). The walls of the vestibulum (=medial surfaces of the premaxillae) are concave and smooth, bearing eight large foramina on the left premaxillary vestibular wall, and seven large foramina on the right premaxillary vestibular wall. What is still present of the palatal premaxillary processes reveals a smoother surface texture than that on the dorsal surfaces. Because of the relatively large incisive foramen, the palatal processes of the premaxillae are not very expansive anteriorly. The anterior laminae of the alveolar processes do not have a well-preserved surface textures, however, it can be deduced that they are rugose, with a few neurovascular foramina visible along the right posterior alveolar process.

**Maxillae**

The maxillae are broken and missing large sections, with modest anterior and posterior portions of both the left and right maxilla being the only remains of these elements (Figs. 9, 10A, 10B, 11A, 11B, 12, 13, 14A and 14B). Because of their incompleteness, precise estimates of the rostrum’s length and width are precluded (although as preserved, the width of the rostrum at the level of the 5th maxillary alveoli is ~315 mm). The anterior maxillary pieces are glued to the premaxillae (CMC2019-010-2; Figs. 9, 10A, 10B, 11A and 11B), whereas the right posterior piece (CMC2019-010-1; Fig. 12) is isolated. The left posterior maxilla (CMC2019-010-4; Figs. 13, 14A and 14B) is glued to the anterior process of the jugal.

The left anterior maxillary piece, glued firmly to the left premaxilla, is more complete and generally better preserved than its right counterpart, preserved posteriorly up to the 5th alveolus. Unlike the left, the right anterior maxillary piece is broken in two parts—the anterior-most part (preserved approximately for the length of the first two alveoli) is still in sutural contact with the right premaxilla, while the posterior part is loosely glued to the aforementioned. The premaxillary-maxillary suture is visible ventrally on the right side and directed towards the posterior of the palatal surface, although it is not preserved along its entire length since much of the palatal processes of the maxillae and premaxillae are missing. What little is preserved of the maxillary palatal processes (close to the medial, or lingual, laminae of the alveolar processes) is unornamented and with relatively smooth textures. In both anterior maxillary pieces, the lingual alveolar margins are very rugose and slightly convex by loosely following the curvature of the lingual alveolar rims. The external (=lateral) surface of the right anterior maxillary piece is heavily ornamented with prominent ridges and grooves. Large neurovascular foramina are present all along the lateral lamina of the maxillary alveolar process, also extending slightly more dorsally away from it. The external surface on the left anterior maxillary piece is somewhat poorly preserved, but the ornamental pattern and neurovascular foramina distribution looks the same as on the right side. As evidenced by the left anterior maxillary piece, the largest preserved tooth/alveolus is the 5th, which consequently causes a wide swelling (specifically, caused by the root of the tooth) on the dorsolateral surface of the maxillary ascending process (Fig. 9). Immediately anterior to this swelling, the maxillary surface is slightly concave. The more complete left anterior maxillary piece has an undulating ventral profile, which is indicative of festooning in the upper jaws.

As preserved, the right posterior maxillary piece (CMC2019-010-1; Fig. 12) has an anteroposterior length of ~170 mm. Its external surface is most prominently ornamented with ridges and grooves at the anterior preserved region. More posteriorly, CMC2019-010-1 has a less intensely ornamented external surface. Large neurovascular foramina are spread on the lateral lamina of the alveolar process and fewer foramina are present more dorsally as well, otherwise, the alveolar process is relatively smooth. A very faint festoon is evident in lateral view, particularly at the level of the last alveolus. Posteriorly on CMC2019-10-1, the rugose sutural surface for contact with the anterior jugal process is exposed. The medial surface of CMC2019-010-1 is partially damaged, although the preserved surface has a smooth texture. Several concavities are present on the anterior part of the medial surface, while approximately in the middle is a perforation that resulted from breakage.

The left posterior maxillary piece is glued to the anterior process of the left jugal (CMC2019-010-4; Figs. 13, 14A and 14B), and it is not as well preserved as CMC2019-010-1. It is preserved for the length of the last five to six maxillary alveoli (anteroposterior length as preserved = ~133 mm). The ornamentation on its external surface matches that of CMC2019-010-1. Like in the right posterior maxilla, the medial surface on the left is also characterized by several concavities, with a shallow, ovoid depression being present on the medial (lingual) laminae of the alveolar processes of both maxillary pieces.

**Nasals**

What is still preserved of the nasals is comprised primarily of their posterior sections that are connected to the anterior process of the frontal posteromedially and the left lacrimal posterolaterally (part of CMC2019-010-3; Fig. 15). Much smaller portions of the anterior nasals are preserved as a separate broken piece that was glued to the premaxillae (part of CMC2019-010-2); this portion of the nasals has a more crudely preserved dorsal surface as compared to the larger posterior sections. The internasal suture is not visible either dorsally or ventrally on both the posterior and anterior sections, and it could not be detected from the CT scanned data either, despite the fact that other sutures are easily observable in the CT scans. This could be an indication that the nasals in the ‘Geoff Vincent’s specimen’ are fused—fusion of the nasals can occur in large and mature crocodylians, as observed in large *C. porosus* specimens (e.g., QMJ39853 and an unnumbered skull at The University of Queensland); this condition also seems to be present in the *B. wickeni* specimen NTM P91171-1.

The more substantially preserved posterior section (CMC2019-010-3) manifest the nasals as flat and relatively thick elements (dorsoventral thickness ~20 mm), with grooves and ridges intensely ornamenting their dorsal surface. The lateral edges expose the sutural surfaces for contact with the (now missing corresponding portions of the) maxillae. As preserved, their lateral margins appear sub-parallel. Ventrally, the nasals have smooth surfaces although affected by superficial cracks. Several small foramina are visible on the posteroventral surface of the left nasal. Three longitudinal ridges, which are continuous from the ventral surface of the anterior frontal process, are spread on the ventral surface of the nasals, with the ridge in the middle being the most pronounced. The middle ridge is delimited from the blunter lateral ridges by smooth and gently concave surfaces. Amongst the specimens available to us for assessment, such ridges on the ventral surface of the frontal and nasals were also noticed in *C. porosus*.

The anterior sections of the nasals (part of CMC2019-010-2) are incomplete, preserved for only a short length (~88 mm). This anterior nasal piece has a gently convex dorsal surface, and widens laterally as it starts to wedge between the premaxillae before it tapers anteriorly. The anterior-most tips of the nasals (i.e., the internarial processes) are broken off, and thus, how far they entered into the external narial fenestra posteriorly is unknown, although it is likely they did not extend significantly within the fenestra, as can be inferred from PVM 89-1072 (Fig. 4). On the ventral surface, the middle ridge is also present on the anterior nasals unit, although with lessened intensity, reaching as far anteriorly as the elements are preserved. The lateral ridges are present on the ventral surface of the anterior nasals unit as well, however, they are very blunt and barely detectable.

**Lacrimal**

The relatively large, but incomplete left lacrimal is preserved and glued to the left nasal (part of CMC2019-010-3; Fig. 15). Although it is missing the posterior portion, the majority of the element is mostly complete and in good condition. The lacrimal’s anteromedial margin is in contact with the nasal, and while most of this contact is covered by glue, the lacrimonasal suture is partially discernable in ventral aspect. The lateral margin of the lacrimal reveals the sutural surface for contact with the left maxilla. Additionally, the medial lacrimal margin partially discloses the sutural surface for what is missing of the left prefrontal—together with the lateral margin of the anterior frontal process and the distal tip of the posterior nasal process permit an approximate deduction of the prefrontal’s outline.

As preserved, the lacrimal is longer than wide (~91.7 mm long, ~52.3 mm wide) and relatively thick (~13 mm dorsoventral thickness). The dorsal surface of the lacrimal is primarily facing dorsally, with a small lateral component. Furthermore, the lacrimal’s gently convex dorsal surface is rugose by being heavily ornamented with ridges, pits and grooves. Anteromedially on the dorsal surface, the lacrimal bears a slightly bulged rugosity, however, due to incompleteness it is unclear if this is related to a possible lacrimal bulge or if it is an isolated ornamental excrescence. Ventrally, the lacrimal is concave and rather smooth, with few small foramina evident more medially on the ventral surface.

**Prefrontal**

A very small piece represents the only preserved segment of a prefrontal, corresponding with the posteromedial portion of the bone contributing to the anteromedial margin of the orbit. This piece is on the left side of CMC2019-010-5 and glued to the frontal (Figs. 16–18). As preserved, its anteroposterior length is ~27 mm, its transverse with ~40 mm and dorsoventral height of ~48 mm. As this is a very small part of the prefrontal, not much can be described of it, and unfortunately it does not inform significantly of the prefrontals morphology. The sutural contact with the frontal in dorsal view is obliterated by glue, however, the suture is clearly visible laterally and ventrally between the two elements (i.e., between the orbital lamina of the prefrontal and descending process of the frontal). The medial margin of the prefrontal piece, which corresponds to an anteromedial section of the left orbital margin, is very rugose and slightly elevated by a bulge. The orbital rim as comprised by the frontal, however, is not elevated like on the prefrontal. Otherwise, the ornamentation/rugosity of the entire medial rim on the left orbit is consistent and confluent as comprised by the prefrontal piece and the frontal. The orbital lamina of the prefrontal is smooth and faintly concave, having two modest foramina piercing it. A scar can be seen anteromedially on the orbital lamina that is caused by breakage where the descending process of the prefrontal (that formed the dorsal part of the prefrontal pillar) would have been. The medial broken surface of the prefrontal piece loosely articulates with the posteromedial piece (?anterior process of the frontal) that is a part of CMC2019-010-3, demonstrating that these two units are of the same skull.

**Postorbitals**

The anterolateral portions of the cranial table are comprised by the postorbitals (Figs. 16–20). The left postorbital is mostly complete, except for missing a significant component of its descending process, and there is also shallow surficial damage anteriorly on its dorsal lamina. As the broken and partially preserved anteromedial process is the only remaining section of the right postorbital, this description will be based on its more complete left counterpart.

The postorbital is a triradiate element, with clearly definable yet stout anteromedial and posterior processes, and a descending process. The anteromedial process contacts the frontal and parietal medially, while the posterior process contacts the squamosal posteriorly. Also, the capitate process of the laterosphenoid fits in a discreet concavity on the ventral postorbital surface, just posteroventral to the dorsoventrally squat and smooth orbital lamina of the postorbital (Fig. 23). Upon external observation of CMC2019-10-5, the sutures between the right postorbital and its adjacent elements could not be clearly distinguished on the dorsal surface. Similarly, the full extent of the suture on the left side between the anteromedial postorbital process, the frontal and parietal is difficult to interpret. Likewise, the suture between the posterior postorbital process and the anterior squamosal process is difficult to observe on the dorsal surface—however, the suture with the latter is evident medially (i.e., on the lateral wall of the supratemporal fenestra), laterally and ventrally. Fortunately, all of these sutures are visible in the CT scan, and are illustrated in Fig. 17. The dorsal lamina (i.e., dorsal surface) of the postorbital is flat, with a blunt and sub-rounded anterolateral margin. Its lateral margin is very rugose, thickened and covered with foramina. The ornamentation on the dorsal lamina is comprised almost exclusively of grooves and ridges, with few pits. Medially on the dorsal lamina of the posterior process and close to the anterolateral margin of the supratemporal fenestra is an especially long and deep, diagonally oriented ornamental groove (Fig. 19). The surface texture on the ventral lamina is smooth, with a single large foramen situated posterolaterally to the capitate process of the laterosphenoid.

The sulcus for attachment of the upper earlid is horizontal and spread over the lateral surfaces of the postorbital and squamosal, and while most of the sulcus is stretched on the squamosal’s lateral margin, the postorbital accommodates a comparatively deeper segment (Fig. 18). The smooth sulcus is a longitudinal concavity on the lateral margin of the supratemporal arch, serving as an intermediate surface between the rugose dorsolateral and smooth ventrolateral edges of the arch. The dorsolateral edge of the arch that is formed by the anterolateral portion of the postorbital is particularly rugose and overhangs the smooth ventrolateral portion of the element that continues ventrally as the descending postorbital process (character 136, state 1). The descending postorbital process is incomplete but would have formed the dorsal part of the postorbital bar. A large circular foramen pierces the postorbital just dorsal to the descending process. Although incomplete, the inset descending process is laterally inclined and has a preserved anteroposterior length of ~27 mm (measured at the dorsal margin of the process), whereas mediolaterally, the process seems somewhat compressed (~15 mm wide, as measured just ventral to the large vascular foramen). The squamosal abuts the posterior margin of the descending process, with the preserved external surfaces of the process being smooth.

**Frontal**

A substantial portion of the frontal is preserved (Figs. 15–19 and 23), although it has suffered some fractures and breakage that resulted with the element missing some of its right descending process (=crista cranii frontalis; frontal descending lamina of Sertich & O’Connor, 2014) and orbital margin. Thus, most of the frontal is still connected to the cranial table unit (CMC2019-10-5), except for the frontal anterior process which is connected to the nasals (CMC2019-10-3).

The frontal comprises a significant portion of the cranial table, being in firm sutural contact with the parietal posteriorly, the anteromedial postorbital processes posterolaterally, prefrontal anterolaterally, and the posteroventral edges of the descending frontal processes contact the dorsal edges of the laterosphenoid orbital surfaces. The frontoparietal suture is difficult to see externally, but fortunately, the CT scanned data reveals its full extent (Fig. 17). The frontoparietal suture is relatively straight for most of its length until its midline where it is anteriorly concave such that the frontal forms a short and blunt posteromedial process wedged anteromedially into the parietal (character 151, state 0). As previously mentioned, the anterior frontal process contacts the nasals anteriorly, and the rugged sutural surfaces for contact with the missing prefrontals are exposed laterally on the same. The frontal formed part of the medial orbital margins, as evidenced by the largely complete medial margin of the left orbit. Just a small extent of the right orbital margin is preserved on the frontal, however, enough to infer that the interorbital space was relatively wide (= ~95 mm). The orbital margins are very rugose and medially concave. The frontal’s descending process is gently concave and smooth. The sharp ventral edges of the descending processes bounded the trough for the olfactory tract, which at its posterior preserved portion has a width of ~10 mm.

The dorsal lamina of the frontal is heavily ornamented with grooves, ridges and few pits, but also shallowly concave medially, slightly posterior to the orbital margins. Towards the suture with the parietal, the dorsal frontal lamina loses the concavity and consequently gets slightly elevated. Posteriorly, at the frontoparietal junction are two large subcircular pits that are discussed in more detail below (see “Parietal” subsection). The texture on the dorsal lamina of the anterior frontal process is not as clear, but the ornamentation appears similar to that on the dorsal frontal lamina (i.e., grooves and ridges), and is consistent with that on its adjacent nasals. The frontal’s dorsal lamina of *Paludirex vincenti* bears no sagittal crest (character 187, state 0). Though slightly damaged, the lateral margins of the anterior frontal process are parallel to each other, giving the process a sub-rectangular shape. The suture between the anterior frontal process and nasals on the dorsal surface could not be seen. What is possibly the suture between the anterior frontal process and the nasals is visible ventrally (Figs. 15C and 15D). This tentatively interpreted suture appears trident-like, with each ‘prong’ being at approximately the same anterior level. The ventral surface of the anterior frontal process is smooth, but convex, with small foramina near the ‘trident prongs’. The ventral surface of the anterior frontal process also bears three sharp longitudinal ridges that continue over the nasals anteriorly (see “Nasals” subsection above). As on the ventral surfaces of the nasals, the ridges on the anterior frontal process are straight, and between each is a gently concave, smooth surface.

**Parietal**

Although a substantial portion of the parietal (Figs. 16, 17, 19, 20 and 23) remains, the element is missing most of the right posterolateral and a piece of its medial sections. The parietal is subtly severed in half, and the left and right parts of the cranial table are glued together. The dorsal lamina (=dorsal surface) of the parietal forms much of the medial portion of the cranial table. Anteriorly, the parietal contacts the frontal, anterolaterally the postorbitals and posterolaterally the squamosal. Anteroventrally, the descending processes of the parietal contact the dorsal margins of the temporal laterosphenoid surfaces, although the sutures between them are difficult to discern (Fig. 23). Contact with the quadrates may be expected posteroventrally as well, however, it seems it is not preserved. Finally, the parietal contacts the supraoccipital posteriorly. Unfortunately, precise interpretation of the parietal-supraoccipital contact in this specimen is problematic. The posteromedial piece of the cranium, to which the supraoccipital is a part of, is a separate broken piece that is glued to the rest of the cranial table. Whether this piece is comprised entirely of the supraoccipital, or if it includes the posteromedial portion of the parietal as well is difficult to determine due to the preservational condition that obstructs any potential sutures. This piece is described in detail below (see “Supraoccipital” subsection).

The proportionately large parietal dorsal lamina appears to have a gently concave surface (unless we accept that the supraoccipital has no dorsal exposure, which would imply that the posteromedial portion of the cranial table, consisted exclusively by the parietal, is not concave like its more anterior region; read below) along the interfenestral bar that is also heavily ornamented in a similar manner to the rest of the cranial table. The ornamentation near the contact with the postorbital and squamosal is made primarily of grooves and dull ridges; more medially on the dorsal parietal lamina, the poor preservational state hinders interpretation of the ornamentation. Anteromedially, crossing over the parietal-frontal suture on the dorsal surface is a faint convexity. Anterolaterally on the left side of the parietal’s dorsal lamina, at the junction with the frontal and very close to (but apparently excluding) the postorbital is a distinct, moderately-sized pit (Figs. 16A, 16B, 17D, 17E and 19; character 211, state 1). A somewhat shallower, yet corresponding concavity is also present on the right side, but it is not as apparent due to the worsened preservation on the right side. These paired pits set anterior to the supratemporal fenestrae are like those seen in *Kambara* Willis, Molnar & Scanlon, 1993.

The interfenestral bar, comprised of the parietal, is hourglass shaped in dorsal view with anterior and posterior mediolateral extensions. Due to the small-sized supratemporal fenestrae, the interfenestral bar is relatively wide (character 208, state 2). However, the width of the bar is not consistent, by being most constricted at the level of the anteromedial corners of the fenestrae (~45 mm transverse width). The width of the interfenestral bar increases gradually towards the posterior, along the medial margins of the fenestrae, until it attains its widest point at the sutural contact between the parietal and medial squamosal process; because the right posterolateral part of the cranial table is missing, the maximum width of the bar and thus the parietal cannot be measured reliably. The postfenestral bar (formed by the medial process of the squamosal and parietal) separating the posterior margin of the supratemporal fenestra from the posterior margin of the cranial table is also very broad (~42 mm minimum anteroposterior length; character 209, state 0). The ventrally directed descending processes of the parietal are sub-vertical and not noticeable in dorsal view through the supratemporal fenestrae. The better-preserved left descending process has a deeply concave lateral (=external) surface. As can be seen on the left side, formed largely by the parietal descending process but also the temporal surface of the laterosphenoid, the medial parietal wall of the supratemporal fenestra is pierced by four conspicuous foramina (character 154, state 1; Fig. 23).

**Squamosal**

Only the left squamosal is preserved, forming the posterolateral portion of the cranial table (Figs. 16–20and 23). While its preservational quality is relatively good, the squamosal is incomplete, having retained only its anterior and medial processes. The sutures between the squamosal and its conjoining elements are difficult to trace on the dorsal cranial surface, however, they are visible around the rims of the supratemporal fenestra, as well as laterally (along the supratemporal arch for the postorbital-squamosal suture) and ventrally. The sutures can be seen with great clarity in the CT scans (Fig. 17). Although the overall shape of the squamosal cannot be evaluated due to incompleteness, it can be noted that in dorsal view, the lateral squamosal margin gets slightly constricted posterior to the contact with the posterior postorbital process, which indicates that the laterodorsal outline of the element was not straight. The entire dorsal lamina of the squamosal is heavily ornamented with large pits, grooves and ridges, with the dorsolateral portion being on a slightly lower horizontal plane relative to the adjacent dorsal laminae of the postorbital and parietal. Additionally, the squamosal’s dorsal lamina is relatively flat, except for a small faintly concave surface seen posteriorly, near the junction of the anterior and medial processes. This very shallow concavity is delimited by faintly elevated and flat surfaces, one positioned along the lateral and the other along the posteromedial squamosal margin. The breakage on the squamosal occurs immediately posterolateral to the aforementioned concavity.

The anterior and medial squamosal processes are broad but short extensions of the squamosal dorsal lamina, with the medial process being the slightly longer one. The anterior squamosal process contacts the posterior postorbital process, and the medial squamosal process is in contact with the parietal. In occipital aspect, the posterior margin of the medial squamosal process has a smooth surface. Most of the sulcus for attachment of the upper earlid is spread on the lateral surface of the squamosal, which is slightly shallower in comparison to its anterior portion found on the postorbital (Fig. 18). The sulcus for the upper earlid musculature attachment is well defined (unlike the relatively indistinct sulcus in the holotype of *Harpacochampsa camfieldensis* Megirian, Murray & Willis, 1991, NTM P87106-1), slightly overhung by the dorsolateral rim of the squamosal, and with a smooth face that is covered by tiny foramina. Laterally on the supratemporal arch, the postorbital-squamosal suture is irregularly shaped such that the anterior squamosal process extends to the point where it abuts the descending postorbital process. Ventrally, the squamosal has a smooth texture, except for the rugose sutural surface on the anterior squamosal process that served for contact with the now lost anterodorsal process of the quadrate. The preservation of the squamosal indicates that both the quadrate and quadratojugal were prevented from making contact with the postorbital (character 143, state 0). Two moderate-sized foramina pierce the squamosal at the posterolateral wall of the supratemporal fenestra, at the junction of the anterior and medial squamosal processes.

**Jugal**

The left jugal is preserved as two separate pieces—–one piece, which is glued to the posterior portion of the left maxilla (CMC2019-010-4), preserves the anterior and ascending processes of the jugal, and the second (CMC2019-010-7) preserves part of the posterior jugal process which is connected to the central plate of the quadratojugal (Figs. 13, 14A, 14B and 21). The anterior jugal process has a broken anterior portion but is otherwise mostly complete and relatively well preserved. An intermediate portion that would have connected the two jugal pieces is missing.

The anterior process of the jugal is broad dorsoventrally, especially at its more anterior section, with gently concave ventral and convex dorsal margins. The dorsal margin of the anterior process would have formed the ventrolateral margins of the left orbit and laterotemporal fenestra, with the ascending jugal process indicating where the two openings were delimited. This entire edge is more distinctly rugose than the ornamentation on the lateral surface, is sloping posteriorly and is mediolaterally thickened. The lateral surface of the anterior process is very rugose and heavily ornamented with irregular excrescent grooves, thickened ridges and small pits that are primarily concentrated in the middle of the preserved element, not too distant from the orbital margin. Near the ventral margin on the lateral surface the texture is smoother, yet still covered in ridges and large vascular foramina. The ventral margin of the anterior process is relatively thin, and the ornamented portion is facing laterally (character 205, state 0).

In medial aspect, the surface of the anterior process is smooth, but has endured some damage. Immediately anterior to the ascending jugal process is a large perforation surrounding the jugal foramen that partially disfigures its contours, that nonetheless indicates its relatively large size (character 102, state 1). A gentle but wide concavity is located at the posteroventral portion on the medial surface, ventral to the ascending process (Figs. 13C, 13D, 14A and 14B). This ventromedial concavity on the jugal is considered an autapomorphy of *P. vincenti*, and it is also present on both jugals in specimen PVM 89-1072 (Figs. 14C–14F). The breakage at the posterior portion of the anterior jugal process shows that the jugal is mediolaterally compressed, although the element is nonetheless appropriately robust due to the large size of the individual.

What is still present of the ascending jugal process is located on the medial side of the anterior jugal process (Figs. 13C, 13D, 14A and 14B). The ascending process of the jugal formed much of the ventral portion of the postorbital bar. The ascending jugal process is incomplete, being broken dorsally and apparently ventromedially as well. Fused medially to it is a small piece from the ascending ectopterygoid process—this is the only preserved remain of the ectopterygoid in the *P. vincenti* holotype, and due to its meager presence it is fairly nondescript, but demonstrates that the ectopterygoid also contributed to the postorbital bar (character 132, state 0). A sutural scar for the ectopterygoid is discernable, spreading ventral from the ascending jugal process—this sutural scar is also visible posteromedially on the maxillary piece and is adjacent to at least the last two maxillary alveoli, however, it is difficult to trace through its entire extent. In PVM 89-1072, the sutural scar of the ectopterygoid appears to be adjacent to the last four maxillary alveoli (character 104, state 0). Like the medial surface of the jugal, the ascending jugal process is smooth and unornamented throughout its entire preserved surface. As preserved, the ascending jugal process has equal dimensions (anteroposterior length = ~17 mm; mediolateral width = ~17 mm). It is also deeply inset from the lateral surface of the jugal, separated from the latter by a smooth trough (character 135, state 1). This condition is like that of *Baru* Willis, Murray & Megirian, 1990 (NTM P91171-1; see Yates, 2017), *Crocodylus, Kalthifrons* Yates & Pledge, 2016 (SAM P35062) and *Kambara* (QMF21127, QMF29662; see Salisbury & Willis, 1996, Buchanan, 2009), but unlike *Mekosuchus* Balouet & Buffetaut, 1987 which has an ascending process of the jugal that is flush with the lateral jugal surface (character 135, state 0; Buffetaut, 1983; Balouet & Buffetaut, 1987; Mead et al., 2002; see Fig. S1.11C in Supplemental Document S1). Other mekosuchines have ascending jugal processes that are comparable to those of *Mekosuchus*, albeit subtly inset (*Australosuchus* Willis & Molnar, 1991, QMF18102; *Trilophosuchus* Willis, 1993, QMF16856). The ascending jugal process of *P. vincenti* is inclined medially, and it is also proportionally slender (character 133, state 1), as opposed to the more robust ascending jugal process of *H. camfieldensis* (NTM P87106-1, see Fig. 6D in Megirian, Murray & Willis, 1991; character 133, state 0).

The posterior process of the jugal is a smaller preserved section compared to the above described. Similarly to the anterior jugal process, the posterior is also heavily ornamented on its lateral (i.e., external) surface (Fig. 21). The ornamentation reaches the ventrolateral margin, yet ventromedially the posterior jugal process is smooth and unornamented. The posterior-most portion of the posterior process is gradually tapered and does not reach the posterior-most preserved edge of the quadratojugal.

**Quadratojugal**

The partially preserved central plate of the left quadratojugal is connected to the posterior jugal process (Fig. 21). Although disarticulated, the quadratojugal was also in firm contact with the quadrate body, as demonstrated by a relatively broad sutural surface that is now exposed along the medial margin of the element. Its dorsolateral surface is heavily ornamented with deep furrows and ridges that are mostly concentrated anteriorly on the preserved plate, while towards the posterior the ornamentation is replaced by pits until it culminates with a swollen, pitted excrescence. The ornamentation is ceased at the posterior preserved sections. The ventral surface of the quadratojugal plate is smooth and gently concave, pierced by a moderate foramen near its posterior.

**Pterygoids**

Most of the pterygoids are missing, with the only preserved portions being their ascending processes (=basisphenoid process of pterygoid sensu Dufeau & Witmer, 2015) that are exposed ventrolaterally on the basicranium (QMF59017; Figs. 24–26). A more considerable part of the left ascending pterygoid process is preserved as opposed to the right. The external surfaces are generally smooth and covered by dim striae, as well as two very small foramina on the left side. A small segment of the pterygoid is also present ventrally on the left half of the basicranium that probably formed the posterodorsal wall of the secondary choana (sensu Witmer, 1995), and has a smooth surface that is minimally concave at its lateral-most corner. The same flake of bone, at the posteroventral-most side contributes to the ventral margin of the median pharyngeal tube foramen (sensu Young & Bierman, 2019; =ostium for the median pharyngeal sinus sensu Dufeau & Witmer, 2015), located immediately to the ventral-most edge of the basisphenoid (Figs. 24A, 24B, 25C and 25D). The anteromedial part of the basicranium that would have been composed of the basisphenoid (primarily the basisphenoid rostrum) and pterygoids has suffered substantial damage, obliterating almost the entire anteromedial contact of these elements (Figs. 24C and 24D). The preserved pterygoids contact the basisphenoid posteriorly and anterodorsally, and they also have posterodorsal contact with the quadrates. The sharp crest B (sensu Iordansky, 1964) of the quadrate transitions over to the pterygoidal surfaces, present as blunt and gently curved sub-vertical ridges spread anteriorly to the sutures with the basisphenoid.

**Chondrocranial bones**

**Laterosphenoids**

The two laterosphenoids are connected to cranial piece CMC2019-010-5. Both laterosphenoids are partially preserved as they are missing their bodies (sensu Holliday & Witmer, 2009), with the dorsal portions (i.e., the postorbital and capitate processes, orbital surfaces and the dorsal parts of the temporal surfaces; see Holliday & Witmer, 2009 for terminology) being the only remaining sections of the elements. Minor and poorly preserved ventral fragments of the laterosphenoids (and possibly the prootics) may also be present on the basicranial unit (QMF59017), lateral to the basisphenoid, however, these small pieces are very poorly preserved and non-descript; it is also difficult to confirm that they indeed belong to the laterosphenoids (or prootics). All exposed and preserved surfaces of the laterosphenoids are smooth. In ventral aspect, the breakage reveals the bony trabeculae inside the bones (Fig. 23A). The right laterosphenoid is slightly more complete than the left. Anterodorsally, the laterosphenoids (specifically, their orbital surfaces; =laterosphenoid anterolateral laminae sensu Kley et al., 2010) contact the frontal (specifically, the posterior of the frontal’s descending processes), the capitate processes contact the postorbitals, and posterodorsally (i.e., the dorsal portions of the temporal surfaces; =laterosphenoid posterolateral laminae sensu Kley et al., 2010) they are in contact with the parietal (specifically, the parietal’s descending processes). The suture with the left postorbital is visible just posterior to the left capitate process (Fig. 23B). Although the contact between the laterosphenoids and the descending parietal processes is implicit, the sutures between them are not obvious.

The columnar postorbital process of *P. vincenti* (Figs. 16C, 16D and 23) is relatively verticalized, similarly to mature *C. porosus* and *K. implexidens* Salisbury & Willis, 1996 (J. Ristevski, 2018, personal observations). Lying laterally on the postorbital process is the partially preserved cotylar crest (sensu Busbey, 1989 and Holliday & Witmer, 2009; =longitudinal oblique crest sensu Iordansky, 1964; Fig. 23). The cotylar crest is blunt and not immediately obvious upon visual inspection yet is conspicuous on touch. The cotylar crest is spread just ventral from the capitate process dorsally and oriented posteroventrally, although its full ventral extent is lost due to breakage on the laterosphenoid. More anteriorly and slightly ventrally to the cotylar is the tensor crest. The tensor crest is thinner and more acute than the cotylar crest, and what is present of it appears to be gently bowed anteriorly when viewed from a lateral perspective (Fig. 23). As mentioned above, the capitate process contacts the postorbital, and on the more complete left side, the capitate process fits in a concavity located on the postorbital’s ventral surface. Both the left and right capitate processes have experienced slight superficial damage. The orbital surfaces contact the ventral edges of the frontal’s descending processes, with their dorsal edges (i.e., at the sutural contact with the frontal) oriented and inclined anteromedially. The anteromedial-most margins of the orbital surfaces are slightly damaged, yet nonetheless sufficiently complete to affirm that dorsomedially they bounded the lateral margins of the olfactory foramen (foramen for cranial nerve I). The anteromedial and dorsal-most edges of the laterosphenoid orbital surfaces (contributing to the margins of the olfactory foramen) are positioned anteriorly relative to the capitate processes (character 166, state 1; see also Brochu, 1997 and the supplementary to Mateus, Puértolas-Pascual & Callapez, 2019 for added clarification on this character from the matrix). Along their medial edges, the orbital surfaces have sustained additional, albeit superficial fractures. Medially, the smooth surfaces of the laterosphenoids (=cerebral fossae) are concave (Fig. 23).

**Supraoccipital**

The supraoccipital (Figs. 16–18, 22 and 23) is mostly complete and seemingly undeformed. While its occipital lamina is sufficiently well preserved (Fig. 22), the internal preservation is rather poor, with diminished clarity on the exposed lateral, ventral and anterior surfaces. Upon external examination, the degree of pneumatization is difficult to evaluate (bar a localized area on the ventral surface that exposes some bony trabeculae inside; Fig. 16C), and the supraoccipital’s contribution, or lack thereof, to the tympanic bullae (also not preserved) is likewise unclear. The CT scan data did not reveal extensive pneumaticity within the supraoccipital, and the intertympanic diverticulum is not preserved. Hence, the following description will be devoted to the occipital (and supposed dorsal) morphology of the supraoccipital as that is the best-preserved portion of the element. Most unfortunate is the perplexing relationship between the supraoccipital and the rest of the cranium. The supraoccipital is connected ventrally to the sub-trapezoid piece (?parietal) that comprises the posteromedial portion of the cranial table, which is glued to the cranium along its left lateral surface. However, no clear sutures are evident on this piece to delimit the supraoccipital from the (presumed) parietal, and the likely sutures visible in the CT scan do not reveal the full extent of this relationship (Figs. 17B–17E). A prominent exposure of the supraoccipital on the dorsal cranial surface has been suggested as a putative synapomorphy of the Mekosuchinae (Willis, Molnar & Scanlon, 1993). Therefore, it is plausible that this entire sub-trapezoid piece consists solely of the supraoccipital; if so, then the supraoccipital has significant dorsal exposure, forming the entire posteromedial portion of the cranial table although without excluding the parietal from the posterior edges of the cranium (character 160, state 2). If, however, the supraoccipital does not constitute the entirety of the aforementioned piece, then the supraoccipital of *P. vincenti* has no dorsal exposure (character 160, state 1). Due to this irresolution, both options will be discussed in the description.

Ignoring the sub-trapezoid dorsal surface (which may belong to the parietal), the occipital lamina of the supraoccipital (Figs. 16C, 16D, 22 and 23) occupies the dorsomedial part of the occiput, having a transverse width of ~57 mm as measured at its lateral margins, ventral to the postoccipital processes. In this scenario, when the cranial table is observed from a dorsal aspect the supraoccipital would be completely hidden from view. Assuming no dorsal contribution to the cranium by the supraoccipital, then the supraoccipital is quite distant from the dorsomedial margin of the occiput as a result of the thick posterior margin of the (?)parietal—this scenario would imply a rather substantial occipital exposure of the parietal. Medially on the occipital surface is a subtle convexity (=?dorsomedial projection of Iordansky, 1973) that appears ventrally confluent with the supraoccipital, continuing over it as a blunt, but salient sub-vertical median ridge at the center of the occipital lamina—the nuchal crest. The nuchal crest is almost complete, except for its ventral section which is missing due to the scraped ventromedial margin of the supraoccipital (Fig. 22). The nuchal crest has a width of ~6 mm and is not especially sharp, at least not as sharp as the nuchal crest of *C. johnstoni* (e.g., UQSSAL J9), but neither as wide and blunt as the crest of *C. porosus* which tends to have much duller edges. The remainder of the occipital lamina of the supraoccipital, laterally adjacent to the nuchal crest, is flat and devoid of lateral concavities that are present in some crocodylians (e.g. *C. porosus*, *Kambara implexidens*). The postoccipital processes (sensu Kälin, 1933) are small, with smooth dorsolateral surfaces (having suffered slight superficial damage) that gently slope lateroventrally. Although the posttemporal fenestrae are not fully preserved, their medial margins are partly discernable as slit-like openings dorsal to the postoccipital processes (Fig. 22). Another peculiarity is the orientation of the supraoccipital’s occipital lamina, most obvious in lateral view—the element is deflected anteriorly, such that its occipital lamina is oriented posteroventrally (to best observe this feature, see the supplementary 3D PDF that includes an interactive digital model of the *P. vincenti* holotype). Such an orientation of the supraoccipital was not observed in any other Australian crocodylian that has a preserved occiput available for comparison and is at present unique to *Paludirex* (character 228, state 1).

Assuming that the supraoccipital has significant exposure on the cranial table, it can then be stated that the element forms a highly developed and expansive supraoccipital dorsal surface. Although the (possible) dorsal expansion of the supraoccipital is slightly incomplete anteriorly, it would be inferred as roughly trapezoid in shape (character 200, state 0). Relative to the medial squamosal process, the posteromedial portion of the cranial table (under this scenario discussed as the dorsal expansion of the supraoccipital) protrudes significantly more posteriorly (Figs. 16 and 17B–17E). In occipital aspect, the dorsal surface of the supraoccipital emerges dorsally to the dorsomedial margins of the posttemporal fenestrae, broadly separating (~20 mm) the occipital lamina of the supraoccipital (described above) from the posterodorsal margin of the cranial table. The most glaring feature is the transverse tuberosity situated along the posteromedial margin of the cranial table (Figs. 16A, 16B, 18, 20 and 22). This transverse tuberosity (~59 mm in transverse width, and ~20 mm in anteroposterior length) has a convex surface and is ornamented with small to moderate sized pits, which contrasts with the ornamentation immediately anterior to it, as well as that on the rest of the cranial table (Fig. 20). The posterior margin of the tuberosity is rugose, and its right lateral margin has suffered slight damage. Another unusual feature of the posteromedial cranial table is that the dorsal surface gradually inclines from its anterior-most preserved portion until it peaks posteriorly at the transverse tuberosity. Among the known Australian crocodylians, a posteromedial cranial table margin that is gently inclined and bears a transverse tuberosity appears to be unique to *P. vincenti*.

The ambiguity of the parietal-supraoccipital contact is inconvenient, and a verdict on this issue depends on examination of better-preserved cranial tables referable to *P. vincenti*. Regardless of which above-discussed morphology is accurate, either would depict a distinct and hitherto unrecognized supraoccipital complex among Australian crocodylians. If the supraoccipital has no dorsal exposure on the cranial table, then it is distinct mainly because of its wide separation from the dorsal cranial edge by a very tall occipital exposure of the parietal, and the curious posteroventral orientation of its occipital lamina. If the supraoccipital has dorsal exposure, then in addition to the above-described form, it can be further distinguished thanks to the highly exposed trapezoidal dorsal surface that occupies a hefty portion of the cranial table, and its unusual inclination and transverse tuberosity.

**Otoccipitals**

Most of the otoccipitals (opistothics-exoccipitals) are broken off with only their ventral portions remaining, specifically those forming the ventrolateral margins of the foramen magnum (i.e., the descending pillars sensu Kley et al., 2010) and the ventral descending processes (Figs. 24–26). Of the two, the right otoccipital is more complete. Externally (i.e., on the occiput), the surfaces of the otoccipitals are smooth and only slightly concave. Due to damage on the posterior occipital surface of QMF59017, the right descending process is missing its ventral ‘tip’, or ventral most portion at the posterolateral region of the basicranium. This damage affects the basioccipital the most, and to a smaller extent the basisphenoid as well (Figs. 24A and 24B; see “Basioccipital” and “Basisphenoid” subsections below). As preserved, the otoccipitals are in contact with the basioccipital ventrally and ventromedially, and laterally with the quadrate pterygoid processes. In addition to comprising the ventrolateral margins of the foramen magnum, the descending pillars of the otoccipitals contribute to the dorsolateral parts of the occipital condyle neck. The preserved external (occipital) surface of the right otoccipital is mostly occupied by cranial nerve foramina (the anterior and posterior hypoglossal foramina, and the common foramen for cranial nerves IX-XI) and the posterior carotid foramen. On the occipital surface of the left otoccipital, only the anterior and posterior hypoglossal foramina remain (with only the posterior hypoglossal foramen being intact, although see “Cranial nerve canals” subsection below), along with a partial canal for the cerebral carotid vasculature (Figs. 24, 25A and 25B).

The descending pillars are robust and relatively long anteroposteriorly, spread from near the basioccipital-basisphenoid suture anteriorly, and occupying almost the entire dorsal length of the occipital condyle neck (Figs. 25A and 25B). Medially, each of the subtly concave surfaces of the descending pillars is pierced by two hypoglossal (cranial nerve XII) foramina, a smaller anterior and a larger posterior hypoglossal foramen, set one in front of the other. Corresponding hypoglossal foramina are opening on the occiput as well, with the small anterior hypoglossal foramen located immediately medial to the common foramen for cranial nerves IX-XI and their associated vessels (i.e., the metotic foramen, or sometimes referred as the foramen vagi; Iordansky, 1973; Bona, Carabajal & Gasparini, 2017; Herrera, Leardi & Fernández, 2018), while the posterior hypoglossal foramen is much more conspicuous on the occiput, opening far dorsomedial to the metotic foramen and close to the occipital condyle neck (Figs. 24A and 24B). It can be remarked that on the occiput, the anterior hypoglossal foramen of the ‘Geoff Vincent specimen’ is not enclosed within a hypoglossal fossa like in *C. johnstoni* or *C. porosus* where the anterior hypoglossal foramina are not as apparent (although a *C. novaeguineae* skull—QMJ5664—has a less enclosed anterior hypoglossal foramen on its right otoccipital as opposed to the left, which may indicate variability of this feature in extant taxa; J. Ristevski, 2018, personal observations). On the medial surfaces, towards the anterior section of the descending pillars are a pair of short and shallow grooves, one on each medial side, rising from the anterolateral portions of the basioccipital and transitioning over the medial surfaces of the otoccipitals, eventually terminating in front of the anterior hypoglossal foramina. As the tympanic bullae are missing, the metotic fissures are obliterated and thus five foramina that would have been partially covered by the bullae are now exposed (Figs. 24C and 24D). In internal aspect, two of these foramina are those for the cerebral carotid arteries and veins (Porter, Sedlmayr & Witmer, 2016), and located immediately dorsal to the communicating ostia between the pharyngotympanic sinus and basioccipital diverticula (see Dufeau & Witmer, 2015). The other three foramina, which open dorsal to the above described internal carotid foramina, relate to the IX-XI nerves (see “Cranial nerve canals” subsection below). On the occiput, the posterior carotid foramen is large and circular (~6 mm in diameter), opening closely ventral to the anterior hypoglossal and common IX-XI foramina. The common foramen for cranial nerves IX-XI and associated vessels (metotic foramen) is the largest external opening on the otoccipital, being transversely elongated (~18 mm transverse width). Both the posterior carotid and metotic foramina are close to the otoccipital-quadrate sutures. Based on the complete left ventral descending process, its tip is tapered and terminates on a slightly more ventral plane than the ventral margin of the occipital condyle, without closely approaching the ventral portion of the basioccipital (character 176, state 0).

**Basioccipital**

The basioccipital (Figs. 24–26) is virtually complete and well preserved, save for some superficial damage on its right posterolateral side and around the occipital condyle. The element constitutes almost the entire posterior portion of the basicranium. The basioccipital is in firm contact with the basisphenoid anteriorly, and with the otoccipitals laterally and dorsolaterally. The occipital condyle neck is formed primarily by the basioccipital (with comparatively smaller dorsolateral contributions from the otoccipitals), and distally on the neck the basioccipital bears the relatively large occipital condyle. The occipital condyle is spherical, slightly wider than tall, being widest at the level of the otoccipital-basioccipital sutures on the occipital condyle neck (~45 mm transverse width, ~38 mm dorsoventral height), and gets transversely narrower ventrally, or approximately at its midline. A long median groove is present on the dorsal surface of the basioccipital, with its posterior-most section reaching the dorsomedial surface of the occipital condyle. The condyle is oriented posteriorly and slightly ventrally, with the outer-most bone surface layer stripped off (except for very small lateral sections), revealing a coarse texture. The ventral and lateral rims of the occipital condyle are rugose.

The dorsal surface of the basioccipital forms much of the posteromedial floor of the braincase (Figs. 25A and 25B). The abovementioned median groove present dorsally on the basioccipital is gently concave, with one of the deepest concavities evident dorsal to the occipital condyle, or just “outside” the foramen magnum. More anteriorly, the basioccipital is set between the descending pillars of the otoccipitals, having sub-parallel sutures with them. The dorsal surface of the basioccipital is smooth and bears multiple tiny and eight to nine more conspicuous foramina. The most peculiar feature dorsally on the basioccipital is a long and pronounced crest (Figs. 25A and 25B). This crest arises approximately halfway on the basioccipital’s dorsal surface and is present as a sharp ridge that transitions anteriorly over to the basisphenoid. The crest is ~40 mm long, with the basioccipital bearing most of its total length (~31 mm or ~77%), and the remainder is found on the basisphenoid. Among all Australian crocodylian specimens that have this portion of the basicranium preserved and available to us for assessment, such a crest was also observed in *H. camfieldensis* (NTM P87106-1, holotype), *Trilophosuchus rackhami* Willis, 1993 (QMF16856, holotype), QMF31075, and we managed to notice it in four *C. porosus* skulls (NTM R12638, QMJ22550, QMJ52809 and an unnumbered specimen at The University of Queensland). The difference between the basicranial crests of the *T. rackhami* holotype (QMF16856) and that in the *P. vincenti* holotype basicranium (QMF59017), is that the crest of *Trilophosuchus* is situated only on the basisphenoid (J. Ristevski, 2019, personal observations). In QMF31075 (Cranial Form 1 as in Willis, 1997; *Ultrastenos willisi* Stein, Hand & Archer, 2016 according to Stein, Hand & Archer, 2016; *Baru wickeni* according to Yates, 2017), a small, partial skull from the Riversleigh World Heritage Area, the crest is faint and similarly to QMF59017 it is spread mostly on the basioccipital, but also on the basisphenoid for a short length. The crest in *H. camfieldensis* (NTM P87106-1) is similar to that in QMF59017. As in QMF59017, the basicranial crest of *Harpacochampsa* Megirian, Murray & Willis, 1991 is also quite long (~27 mm) and situated mostly on the basioccipital, with a very short and blunter portion spreading anteriorly over the basisphenoid (just ~4 mm). On the dorsal surface of the basioccipital, the basicranial crest of *Harpacochampsa* is quite acute and laterally delimited by a few foramina. In two of the aforementioned *C. porosus* specimens (QMJ22550, skull length ~420 mm; and, QMJ52809, skull length ~560 mm; both measured from the premaxillae anteriorly to the posteromedial margin of the parietal posteriorly), the crests were seen on the basioccipital, however, as these are complete and articulated skulls, observation of this feature was limited only through the foramen magnum and any possible extension over the basisphenoid was difficult to see. In *C. porosus* NTM R12638 (skull length ~430 mm, as measured from the premaxillae anteriorly to the posteromedial margin of the parietal posteriorly), the basicranial crest was more clearly observed through the foramen magnum and appeared very similar to those of the *P. vincenti* holotype and the *H. camfieldensis* holotype specimens. While measurements of the crest in NTM R12638 could not be taken as that skull is also articulated, it can be seen that the crest is quite acute and spread primarily over the dorsal surface of the basioccipital with a smaller section crossing over to the basisphenoid. Laterally to the crest on the basioccipital, the crest in *C. porosus* NTM R12638 is also delimited by a few small foramina. The seemingly random appearance of basicranial crests among different taxa, and even within a single species, indicates that this feature is unlikely to be of diagnostic value for crocodylians, including *Paludirex*.

Two large openings (i.e., the communicating ostia between the pharyngotympanic sinus and basioccipital diverticula, or median pharyngeal recesses; see Dufeau & Witmer, 2015) are located anterolaterally on both sides of the basioccipital, at the suture with the basisphenoid (Figs. 25A and 25B; see “Cranial pneumaticity” subsection below).

The basioccipital plate (i.e., the posterior lamina of the basioccipital) is oriented fully posteriorly (character 170, state 1) and interspersed with tiny foramina, while ventral to the occipital condyle neck are four large and two moderate-sized vascular foramina. Most of the plate’s surface is smooth, however, marked rugosities characterize the left lateral and most of the ventral portions of the element (i.e., the portions that correspond with the basioccipital tubera). Ventromedially in occipital aspect, the rugose surface is also convex, yet there is no presence of a basioccipital crest (basioccipital eminence sensu Young & Bierman, 2019) on the basioccipital. In contrast to *P. vincenti* QMF59017, other Australian crocodylians (*Australosuchus*, QMF17983; *Harpacochampsa*, NTM P87106-1; *Kambara*, QMF21129, QMF29662, QMF29663; *Mekosuchus sanderi* Willis, 2001, QMF31166; and, *Trilophosuchus*, QMF16856; J. Ristevski, 2018, personal observations) possess relatively well-developed median crests on their basioccipitals. Both *Crocodylus porosus* and *Crocodylus johnstoni* bear median crests on their basioccipitals as well, and are comparatively more developed in the latter. In larger *C. porosus* skulls, the posteroventral basioccipital portions are also rumpled and may have receded median crests, while more laterally, the basioccipitals develop sub-vertical rugosities. This condition of the basioccipital seems to be fairly comparable between *Paludirex* (QMF59017) and some large *C. porosus*.

Opening ventrally in the center of the basioccipital is the large (~14 mm transverse and ~11 mm anteroposterior length), ocular-shaped median pharyngeal tube foramen (sensu Young & Bierman, 2019; =ostium for the median pharyngeal sinus sensu Dufeau & Witmer, 2015; Figs. 25C and 25D). The internal ventral lamina of the basioccipital that bounds the median pharyngeal tube foramen is concave, corresponding with the aforementioned external convexity. The preservational state of QMF59017 is lessened at the ventral contact between the basioccipital and basisphenoid, and thus, the pharyngotympanic foramina (sensu Young & Bierman, 2019) are not visible.

**Basisphenoid**

The basisphenoid (Figs. 24–26) is well preserved and mostly complete, but missing the basisphenoid rostrum (=cultriform process) anteriorly. Similarly to the damaged right posterolateral portion of the basioccipital, the posterolateral external exposure of the basisphenoid on the right side of the basicranium is effaced. The basisphenoid has preserved its contact with the basioccipital posteriorly, ventral and ventrolateral with the ascending pterygoid processes, and lateral with the quadrate pterygoid processes. The suture between the basisphenoid, pterygoid and quadrate on the left posterior side is clearly visible through most of its length as an anteriorly bent arch, however, the dorsal-most and ventral-most traces of the suture are difficult to deduce (Figs. 26C and 26D). Only some of the equivalent suture is barely detectable on the right side. Relative to the rest of the basicranium, the basisphenoid has modest external exposure on the left posterolateral side of the braincase (~7 mm anteroposterior length, between ~34 mm and ~47 mm dorsoventral length). There, the lateral surface of the basisphenoid is convex and smooth, getting somewhat rugose posteriorly at the contact with the basioccipital. Ventrally, the basisphenoid exposes only a small and narrow piece that forms the anterior margin of the median pharyngeal tube foramen (character 172, state 0). The basisphenoid has additional external exposure anteriorly on both left and right sides as sub-triangular concave sheets of bone. At these anterior portions, the sutures with the pterygoids are ventral to the basisphenoid and sub-horizontal.

Anteriorly, where the base of the basisphenoid rostrum would have been, the damaged surface of the basisphenoid is dorsomedially convergent, adapting a sub-triangular shape. Immediately dorsal to this point is the large hypophyseal (pituitary) fossa (Figs. 24C and 24D). The fossa’s subcircular outlines are relatively impaired (~18 mm dorsoventral height, ~16 mm transverse width), and deep within the fossa are the orifices for the left and right anterior carotid foramina.

Dorsally, the basisphenoid covers a smaller surface compared to the basioccipital (~65 mm total anteroposterior length of the dorsal basicranial surface, as measured from the foramen magnum to the dorsum sellae; basioccipital length = ~42 mm or ~64% of total length, as measured from the foramen magnum to the basioccipital-basisphenoid suture; basisphenoid length = ~23 mm or ~35% of total length, as measured from the basioccipital-basisphenoid suture to the dorsum sellae). In dorsal view, the basioccipital-basisphenoid suture is mildly convex, with the basioccipital lightly wedging into the basisphenoid (Figs. 25A and 25B). The abovementioned basicranial crest is slightly blunter on the basisphenoid’s dorsal surface as compared to that on the basioccipital and stretches approximately 35% of the basisphenoid’s surface length. Anteriorly, the crest trifurcates into very faint (almost imperceptibly so) ‘branches’, with the lateral ‘branches’ reaching the medial margins of the dorsal abducens (cranial nerve VI) foramina. These faint trifurcations give the crest a trident-like appearance dorsally on the basisphenoid. The paired abducens foramina are small yet conspicuous, piercing the dorsal surface of the basisphenoid. The abducens foramina open again at the (poorly preserved) anterior-most portion of the basisphenoid, appearing as small openings dorsolateral to the hypophyseal fossa. The basisphenoid is gently inclined anteriorly, reaching its highest point at the dorsum sellae. For the most part, the dorsal surface is smooth, except for the region around the basioccipital-basisphenoid suture which is slightly rugose and covered by multiple tiny and three medium-sized foramina. Irregularly shaped sub-parallel lateral sutures demarcate the basisphenoid from the ventral preserved portions of what are most likely the prootics and possibly the laterosphenoids. Unfortunately, what little may be present of these elements on QMF59017 is so poorly preserved that additional commenting is futile. Opening anterior to the posterior openings for the cerebral carotid canals on the basisphenoid are two very small communicating ostia related to the basisphenoid diverticulum (Figs. 25A and 25B; see “Cranial pneumaticity” subsection below).

**Splanchnocranial bones**

**Quadrates**

The isolated left quadrate body (CMC2019-010-6) is largely complete and in a good preservational condition (Figs. 27 and 28). The sutural surface for the quadratojugal extends laterally along the element. The quadrate body is quite large, robust and wider than tall, with its maximum preserved length being ~139 mm (measured from the lateral quadrate hemicondyle posteriorly to the anteromedial-most preserved edge). The dorsal surface of the body is flat to faintly convex, while the ventral is only gently concave; the ventral surface is most concave posteriorly near the condylar margins. The dorsal surface is relatively smooth, save for some small-scale rugosities. Most of the anteromedial half on the dorsal surface is covered by a badly preserved sutural remnant of the otoccipital—the only potentially identifiable remnant of the otoccipital here is a small piece that was likely a part of the rounded lateral tip of the paroccipital process, pointing posterolaterally in the direction of the lateral quadrate hemicondyle (Figs. 27A and 27B). Willis & Molnar (1997a) described this lump as a ‘distinct plinth’ that was formed by the squamosal contact; by observing the corresponding area in *C. porosus* skulls, most of the ‘plinth’ in this specimen would have served as contact for the otoccipital, although the lateral side of the ‘plinth’ may have received contribution by the posterior descending lamina of the left squamosal—this is difficult to confirm due to the preservational state. The quadrate body is relatively short, with the width at the quadrate hemicondyles (see below) exceeding the length (~27 mm) as measured from the (possible) tip of the paroccipital process to the condylar surface (character 215, state 0). Along the anteromedial surface, medially to the ‘plinth’ is the lateral wall of the cranioquadrate canal. As the lateral wall of the cranioquadrate canal is exposed, it unveils a concave and smooth surface. A fragment of the otoccipital may be present near the cranioquadrate canal, however, if so, then the preservational state around this area precludes it. In medial profile, the body is dorsoventrally more compressed as opposed to its dorsoventrally taller lateral profile (described as “The lateral sutured margin on the quadrates for the quadratojugal is very deep dorso-ventrally” by Willis & Molnar, 1997a, p. 235).

The quadrate body attains its greatest width posteriorly at the quadrate hemicondyles (~98 mm transverse width as measured from the lateral-most margin of the lateral hemicondyle to the medial-most margin of the medial hemicondyle). The articular (=posterior) condylar surfaces are generally flat and subtly convex, having coarse outer textures akin to that of the occipital condyle. The margins surrounding the hemicondyles are rugose. There is no deep intercondylar groove to clearly demarcate the lateral from medial hemicondyle, which makes the limit between them somewhat arbitrary. The lateral quadrate hemicondyle is larger than the medial (lateral hemicondyle = ~42 mm dorsoventral height and ~47 mm transverse width; medial hemicondyle = ~33 mm dorsoventral height and ~38 mm transverse width) and positioned more posteriorly relative to the latter. In posterior view, the outlines of both hemicondyles appear irregularly oval, and the entire condylar surface has a ‘figure eight’ look (Fig. 28A). Additionally, the rounded medial margin of the medial hemicondyle is oriented medioventrally (character 181, state 0).

The foramen aëreum of CMC2019-010-6 is small yet conspicuous and set mediodorsally on the quadrate body anterior to the medial hemicondyle (characters 177 and 178, states 0; Figs. 28B and 28C). The foramen aëreum is positioned close to the medial hemicondyle (~22.8 mm anteriorly from the medial hemicondyle, with a distance of ~7.5 mm from the medial margin of the foramen to the medial edge of the quadrate) and has a transverse width of ~1.4 mm. The foramen is accompanied by a shallow sulcus that is continuous from the foramen’s opening and spread posteriorly towards the medial hemicondyle. However, this sulcus stretches only a short distance before it becomes flush with the dorsomedial surface of the quadrate. The sulcus has an anteroposterior length of ~6.6 mm and ~2.1 mm transverse width, with laterally diverging margins before they become indistinct. The siphoneal tube coursing through the quadrate body exits posteriorly via the foramen aëreum. The siphoneal tube is narrow (~5 mm) and as digitally segmented has a length of ~74 mm (Fig. 28D).

The ventral surface of the quadrate body is relatively smooth and gently concave, with tiny foramina, almost imperceptible to the naked eye, interspersed over it. No quadrate crests (sensu Iordansky, 1964) are distinguishable on the ventral surface of the preserved quadrate body. A possible exception may be a subtle and very blunt bulge on the ventral surface near the lateral hemicondyle that approximates with the usual location for quadrate crest D in crocodylians (see Iordansky, 1964, 1973).

Parts of the pterygoid processes of the quadrates (=quadrate pterygoid processes) and small sections relating to the dorsal primary heads (sensu Kley et al., 2010) of both quadrates are connected to the basicranial unit (QMF59017), and thus separate from the isolated left quadrate body described above (Figs. 24–26). The right quadrate is more substantially preserved on the basicranial unit as opposed to the left. Here, the quadrates are in contact with the otoccipital remnants posteriorly, and the sutural contacts with the ascending pterygoid processes (=pterygoid quadrate processes sensu Kley et al., 2010; basisphenoid processes of the pterygoids sensu Dufeau & Witmer, 2015) are present ventrally. Interestingly, the suture between the pterygoid quadrate process and the ascending pterygoid process is ventrally reflected (character 168, state 0; Figs. 26A and 26B). This feature is observable on the right side of QMF59017, whereas on the left side the suture is not as clear to see. Contact with the lateral exposure of the basisphenoid is externally visible and brief on the left side, occurring posteriorly; the more substantial contact between the pterygoid processes of the quadrates and the basisphenoid is not exposed, with the basisphenoid being covered laterally by the former. As in the isolated quadrate body, the broken surfaces (as well as CT scans of QMF59017) reveal the trabecular bone inside. All external surfaces are smooth, and only weakly rugose at the immediate vicinity of the sutural contact with the ascending pterygoid processes. Pronounced quadrate crests B stretch along the pterygoid processes of the quadrates, having sharp edges that curve posterodorsally. On the right side, the surface anterior to crest B is gently convex, while posteriorly to the crest the surface is more concave. A sub-elliptic foramen located at the laterodorsal margin on the right dorsal primary head most likely represents the opening that led into the siphoneal tube and exited as the foramen aëreum on the quadrate body (in *C. porosus*, this corresponding orifice opens anteroventrally to the otic buttress sensu Montefeltro, Andrade & Larsson, 2016; J. Ristevski, 2018, personal observations). This short canal was segmented from the CT scanned data (Fig. 32) and is herein interpreted as a segment of the siphoneal tube. A long and concave trench is present on the dorsal surface of each dorsal primary head, pertaining to the pharyngotympanic sinus (sensu Dufeau & Witmer, 2015; =tympanic cavity sensu Colbert, 1946). The trenches terminate at the basioccipital-basisphenoid suture, close to the posterior openings for the cerebral carotid arteries on the basisphenoid, and immediately lateral to the communicating ostia between the pharyngotympanic sinus and basioccipital diverticula. The suture with the right otoccipital is clearly evident dorsal to the ‘pharyngotympanic sinus trench’ (Figs. 24C and 24D). The quadrates, specifically their pterygoid processes, are well visible in occipital view ventrolateral to the otoccipitals (character 214, state 1; Figs. 24A and 24B).

**Dentition**

Most inferences on the dentition of ‘Geoff Vincent’s specimen’ are based on its preserved premaxillary and maxillary alveoli. Although several partial teeth are present, only one (5th maxillary on the left side; Fig. 29) provides useful information of the dental morphology. *Paludirex* displays evident size differentiation between the alveoli (size-related pseudoheterodonty/size-related heterodonty; D’Amore et al., 2019), especially obvious among the anterior maxillary dentition (i.e., at least the first five maxillary teeth) of CMC2019-010-2 (character 222, state 1). Size-related heterodonty is present to various degrees in all Cenozoic Australian crocodylians for which their dentition is at least partially known, both extant (albeit to a much lesser degree in *C. johnstoni* as opposed to *C. porosus*) and extinct (although in *Quinkana* the heterodonty tends to be very subtle; Molnar, 1977, 1981). Heterodonty as related to the shape of the teeth (i.e., caniniform vs molariform) could not be assessed directly in ‘Geoff Vincent’s specimen’ since most of its teeth are missing. All preserved alveoli are circular and without labiolingual compression (character 79, state 0), unlike the labiolingually compressed alveoli of the ziphodont *Quinkana*. The alveolar rims in the *P. vincenti* holotype are rugose, and there is almost no scalloping between them. There are no interdental reception pits for dentary teeth in the preserved maxillae of the *P. vincenti* holotype. In *Paludirex* cf. *P. vincenti* PVM 89-1072, there is no indication of interdental pits on the maxillae either, which implies that all dentary teeth occluded lingual to the maxillary (character 92, state 0)—the exception being the large 4th dentary teeth which were exposed during occlusion between the premaxillae and maxillae. Measurements of the sufficiently complete alveoli in the *P. vincenti* holotype are given in Table 2. Specimen PVM 89-1072 preserves more of its alveoli compared to the *P. vincenti* holotype, which counts 14 maxillary alveoli in its right maxilla (character 223, state 1); their measurements can be found in Table 3. For the remaining *Paludirex* specimens, the measurements for their preserved alveoli are presented in Table 4.

**Table 2:** Premaxillary and maxillary alveoli dimensions of *Paludirex vincenti* gen. et sp. nov., ‘Geoff Vincent’s specimen’, holotype.

Alveolus position	Mesiodistal length (mm)	Labiolingual width (mm)
RIGHT PREMAXILLA (CMC2019-010-2)		
1st	~12	~14
2nd	~13	~16
3rd	~20	~23
4th	~40	~43
5th	~15	–
LEFT PREMAXILLA (CMC2019-010-2)		
1st	~11	~16
2nd	~13	~13
3rd	~20	~24
4th	~41	~40
5th	–	~15
RIGHT MAXILLA, ANTERIOR (CMC2019-010-2)		
1st	–	–
2nd	–	–
3rd	~20	~20
4th	~22	~25
5th	–	–
LEFT MAXILLA, ANTERIOR (CMC2019-010-2)		
1st	~14	~15
2nd	~12	~14
3rd	~22	~25
4th	~24	~29
5th	–	~45
RIGHT MAXILLA, POSTERIOR (CMC2019-010-1)		
anterior-most preserved	~26	~26
2nd preserved	~29	~25
3rd preserved	~24	~21
4th preserved	~19	–
5th preserved (penultimate)	~18	~15
6th preserved (last)	~12	~12
LEFT MAXILLA, POSTERIOR (CMC2019-010-4)		
anterior-most preserved	~25	~25
2nd preserved	~20	~22
3rd preserved	–	–
4th preserved (penultimate)	–	–
5th preserved (last)	~12	~12

**Note:**

Alveoli that could not be reliably measured due to having their rims substantially damaged or missing have omitted data (indicated with a dash).

**Table 3:** Premaxillary and maxillary alveoli dimensions of *Paludirex* cf. *P. vincenti*, PVM 89-1072.

Alveolus position	Mesiodistal length (mm)	Labiolingual width (mm)
RIGHT PREMAXILLA		
1st	~20	~24
2nd	–	~22
3rd	~27	~32
4th	~40	~40
5th	~15	~22
LEFT PREMAXILLA		
1st	~16	~22
2nd	~16	~18
3rd	~25	~28
4th	–	–
5th	–	–
RIGHT MAXILLA		
1st	–	–
2nd	–	–
3rd	~20	~22
4th	~28	~30
5th	~49	~50
6th	~19	~19
7th	–	–
8th	~23	~22
9th	~27	~28
10th	~30	~29
11th	~31	~32
12th	~17	~19
13th	~19	~19
14th	~15	~15

**Note:**

Measurements of the alveoli in the left maxilla are not given due to poor preservation. Other alveoli that could not be reliably measured due to having their rims substantially damaged or missing have omitted data (indicated with a dash).

**Table 4:** Premaxillary and maxillary alveoli dimensions of *Paludirex* gen. nov.

Alveolus position	Mesiodistal length (mm)	Labiolingual width (mm)
*P. VINCENTI* QMF11626—COMPLETE LEFT PREMAXILLA AND PARTIAL MAXILLA		
1st premaxillary	~19	~19
2nd premaxillary	~13	~13
3rd premaxillary	~27	~30
4th premaxillary	~39	~43
5th premaxillary	~18	~16
1st maxillary	~11	~13
2nd maxillary	~15	~15
*P. GRACILIS* QM17065 (HOLOTYPE)—RIGHT PREMAXILLA		
1st	~11	~11
2nd	~11	~11
3rd	~20	~20
4th	~30	~37
5th	~16	~14

**Premaxillary alveoli**

Each premaxilla has five alveoli, the largest of which are the 4th (character 189, state 2). Among those measured, the 1st and 2nd are the smallest with the 1st being slightly smaller, although it appears that the 5th are similarly small as the aforementioned (Table 2). Unfortunately, the damaged alveolar rims of the 5th premaxillary alveoli hamper reliable measurements. The lingual rims of all premaxillary alveoli are not as high as the labial rims—in part, this slight disparity is exaggerated by the dilapidated alveolar rims, however, it seems that it is genuine. The same is true for the preserved alveoli on the posterior maxillary pieces (CMC2019-010-1 and CMC2019-010-4). Both 1st premaxillary alveoli have suffered some damage along their rims. The 1st premaxillary alveoli are situated anteromedially on the premaxillae such that their mesial margins abut the interpremaxillary suture. A substantial interalveolar gap separates the distal margins of the 1st from the mesial margins of the 2nd premaxillary alveoli (~18 mm on the left and ~23 mm on the right premaxilla). The 1st and 2nd premaxillary alveoli are positioned in the same transverse plane, such that each alveolus on the premaxilla is neither more anterior or posterior relative to the other (Figs. 10A and 10B). The same positioning of the first two premaxillary alveoli is present in PVM 89-1072 (Fig. 10C), QMF11626 (Figs. 3C and 3D) and the *P. gracilis* holotype QMG17065 (Figs. 5C and 5D). When the premaxillae are observed in anterior aspect, the 2nd alveoli are positioned on a more ventral plane than the 1st as a result of the distinctive anteromedial curvature of the premaxillary alveolar processes (Figs. 11A and 11B). The alveolar rims of the 2nd premaxillary alveoli are well preserved, bar some tenuous damage of the mesiolingual rim on the right alveolus. Compared to those between the 1st and 2nd, the interalveolar spaces between the 2nd and 3rd premaxillary alveoli are substantially smaller (~4 mm on both left and right premaxillae). The 3rd premaxillary alveoli are immediately posterior to the 2nd, but also positioned more labially. Overall, the alveolar rims of the 3rd premaxillary alveoli are well preserved. The interalveolar spaces between the 3rd and 4th premaxillary alveoli are more accentuated as compared to the spaces between the 2nd and 3rd (~8 mm on the right and ~10 mm on the left premaxilla). In anterior view, the 3rd premaxillary alveoli are set more ventrally relative to the 2nd, and only slightly more dorsally to the 4th. The 4th alveoli are slightly more labial than the 3rd, with their large size conforming to the expansion of the premaxillae which are the widest at the level of these alveoli. Their alveolar rims are well preserved. On the right premaxilla, the interalveolar space between the 4th and 5th alveoli is ~5 mm, while measurement on the left is prohibited due to the ruined alveolar rims of the 5th alveolus. As the alveolar processes of the premaxillae are gently curved posterodorsally, the 5th alveoli are on a more dorsal level than the 4th, most obvious in lateral view. In the premaxillae, remnants of teeth are present within the 3rd and 4th alveoli, as well as the right 5th alveolus. These remnants are tooth roots and/or basal portions of the crowns, however, their very poor preservation and inaccessibility makes them non-descript. The large and deep reception pits for the first dentary teeth (right side reception pit = ~31 mm anteroposterior length and ~29 mm transverse width; left side reception pit = ~32 mm anteroposterior length and ~24 mm transverse width) are located lingual to and between the 1st and 2nd premaxillary alveoli. Shallow reception pits are present lingual to and between the 3rd and 4th premaxillary alveoli. Comparisons of the premaxillary alveoli size ratios among *Paludirex* is shown in Fig. 30.

**Maxillary alveoli**

Beginning with the anterior maxillae (attached to the premaxillae as part of CMC2019-010-2; Figs. 10A and 10B), the first two alveoli are the smallest and positioned more lingually relative to the successive three alveoli. This lingual disposition of the 1st and 2nd maxillary alveoli is due to the constrictions at the premaxillary-maxillary sutures caused by the notches for receiving the 4th dentary teeth. Information on the first two maxillary alveoli is derived from those on the left maxillary piece, since they are better preserved than those on the right. The 1st maxillary alveolus on the left is set subtly more lingual to the 2nd. Observed in lateral aspect, the anterior maxillary festoon affects the arrangement of the alveoli such that the 1st alveolus is more dorsal relative to the other preserved alveoli, followed by the 2nd which is slightly more ventral to the 1st, while the 3rd is slightly more dorsal to the 4th, but still set on a similar horizontal plane as the latter. In ventral (=palatal) view, the lateral expansion/festooning of the maxillae also affects the labiolingual disposition of the alveoli, and in this case the 3rd alveolus is more labial in relation to the first two, while the 4th and 5th alveoli are roughly aligned with each other, but set slightly more labially relative to the 3rd. There is insignificant or virtually no interalveolar space between the anterior maxillary alveoli. Although incomplete and unable to provide exact measurements, it is obvious that the 5th maxillary alveoli are the largest (character 93, state 1). This is akin to many crocodyloids, yet different from species of *Quinkana* (*Q. babarra* Willis & Mackness, 1996, QMF23220; *Q. fortirostrum* AM F.57844; *Q. meboldi* Willis, 1997, QMF31056, QMF31057 and QMF31058; *Q. timara* Megirian, 1994, NTM 895-19 and NTM P9464-168) which have 4th maxillary alveoli sub-equal to, or marginally larger than the 5th. However, as mentioned above, *Quinkana* species have more subtle size-related heterodonty, and their alveolar size differences are not as nearly stark as those of *Paludirex*. All alveoli of the left anterior maxillary piece have teeth remnants in situ. Deeply embedded within the 1st alveolus is a partial hollow root, while within the 2nd and 3rd are partial roots and basal portions of tooth crowns. Like the teeth remnants in some of the premaxillary alveoli, these are also poorly preserved and of no descriptive value. Peering from the matrix filled 4th alveolus is a slightly damaged tooth crown apex. Also visible are the apical portions of the mesial and distal carinae of the same. Due to the inaccessibility and preservational state of this tooth, its description is significantly impeded.

The posterior maxillary pieces preserve the last five (left piece, CMC2019-010-4; Figs. 13C and 13D) to six (right piece, CMC2019-010-1; Figs. 12C and 12D) alveoli, however, their consecutive order in the dental arcade is also undetermined. It should be noted that the distal walls of alveoli are also preserved mesial/anterior to the first complete alveoli in the posterior maxillary pieces. These alveolar walls are non-descript, other than mentioning that their walls are highly rugose with apicobasal ridges and furrows. As opposed to the anterior maxillary alveoli, the posterior have ample interalveolar spaces. In the right posterior maxillary piece (CMC2019-010-1), the two anterior-most alveoli are aligned with each other; the successive three alveoli are subtly offset labially relative to the former, while the last alveolus conforms to the gentle lingual curvature of the posterior maxilla. The subtle lingual curvature is also seen for the last alveolus in the left posterior maxilla. As all except two alveoli (one in CMC2019-010-1 and one in CMC2019-010-4) are empty, it can be observed that the depth of each subsequent alveolus, from anterior to posterior, decreases such that the last maxillary alveoli are shallowest. Also in the posterior maxillary pieces, the labial alveolar rims are on a slightly more ventral plane than the lingual rims; this is not the case with the alveoli of the anterior maxillary pieces, however. The anterior-most preserved alveolus in CMC2019-010-1 has a poorly preserved root embedded in matrix. The root remnant is circular, and its inside hollow. Similarly, the second anterior preserved alveolus in CMC2019-010-4 also has a partial tooth. The last alveolus in CMC2019-010-4 has some matrix within it.

**The 5th maxillary tooth**

The best-preserved tooth in the specimen is the 5th maxillary on the left side (Fig. 29), and thus it is the only one adequate for description. This is a large and fully grown caniniform tooth that probably collapsed into its alveolus post-mortem, leaving its mesiolingual side fused to the mesial alveolar wall. Thanks to the breakage of the left maxilla that severed the 5^th^ alveolus, the labial surface of the tooth is almost entirely exposed. This positioning of the tooth also allows observation of the distal carina, whereas the mesial is obscured from view. The orientation of the tooth is inferred by the gently convex exposed surface (interpreted as labial), and the (lingual) curvature of the distal carina. In all examined *Crocodylus* specimens, if the teeth were curved, they were always curved lingually. Also, the tooth was digitally removed from its alveolus in order to better assess its morphology; its digital model can be seen in the interactive 3D PDF of the *P. vincenti* holotype, available as a supplement.

The crown of the tooth is virtually complete and generally well preserved, except for a missing enamel slice basally, near the cervix (=neck of the tooth, or cervix dentis; see Smith & Dodson, 2003 and Hendrickx, Mateus & Araújo, 2015) and close to the distal carina—this is the most substantial damage visible on the crown, and there are no (or at most, only insignificant) deformations (Fig. 29A). However, the enamel surface is also affected by small and very thin superficial cracks, and some sections are lightly covered by matrix. The crown apex is not very sharp (but not especially blunt either), and upon inspection under a microscope, there is no apparent damage or wear (Fig. 29B). The inside of the tooth is hollow until approximately mid-crown, after which is filled with reddish-brown matrix. Basally, the tooth crown is circular in cross-section. It starts to taper from approximately mid-crown towards the apex, with its overall shape being conical. The mesiodistal width as measured at the cervix is ~28 mm (measured from the digitally segmented tooth), while halfway between the mid-crown and apex is ~16 mm (due to how it is preserved, this is the only measurement that could be taken directly from the tooth). The apicobasal height of the tooth crown is ~48 mm (measured from the digitally segmented tooth). The exposed enamel surface is rather smooth, covered only with fine apicobasally aligned striae and there is no fluting like in *C. johnstoni* and *C. porosus* (character 197, state 0; Fig. 29D). Relatively smooth and non-fluted enamel surfaces can also be inferred from the partially preserved crowns in the 5th premaxillary alveolus on the right side and the crown in the second anterior preserved alveolus in CMC2019-010-4. Lack of fluting was reported for teeth previously referred to *Pallimnarchus* by Willis & Molnar (1997b) and Chiotakis (2018). Around the apex, the enamel texture is faintly anastomosed (Fig. 29B). What is preserved of the root is highly fragmented, with all its external surfaces fused to the alveolar walls. The breakage reveals a smooth inner texture of the remaining root.

The carinae are spread apicobasally from the cervix to the apex. Due to the manner of preservation, description is possible only of the distal carina, since the mesial is obscured from view. The distal carinal keel is conspicuous but not very high, although it becomes more prominent from around the mid-crown to the apex. Upon reaching the apex, the carina becomes more suppressed but not terminated—instead, it crosses over to merge with the mesial carina (Fig. 29B).

The carina bears irregular crenulations that are most easily discernable mid-crown length (Fig. 29C). However, interpretation of the crenulations as true denticles is problematic in this tooth. Significant sections of the carina are obstructed by minute matrix coverings, and the preservation on the adjacent crown regions is also rather poor, which hinders precise evaluation on whether the crenulations are caused by the crown enamel ornamentation spreading over the carina (which would result with false denticles), or if the crenulations on the carina are formed independently and are thus true denticles. At least at the crown apex, it is evident that the anastomosed pattern transfers over the carinae which forms false denticles (=“false ziphodont condition” sensu Prasad & De Lapparent de Broin, 2002). Therefore, we tentatively interpret the carina as having superficial crenulations along its length, but not true denticles (sensu Young et al., 2015; character 80, state 0).

Overall, the described tooth from the ‘Geoff Vincent specimen’ can be categorized as conidont sensu Hendrickx, Mateus & Araújo (2015). The tooth morphology is easy to distinguish from the fluted teeth of *Crocodylus*, and from labiolingually compressed and serrated (true ziphodont) teeth. Hopefully, an in-depth examination of other better-preserved teeth from Chinchilla that correspond with the morphology described in this study could elucidate whether the carinae of *Paludirex* teeth bear false denticles as inferred from the ‘Geoff Vincent specimen’ here.

**Endocranial morphology**

Certain endocranial elements of the *P. vincenti* holotype specimen, mainly from the basicranial unit (QMF59017), but also the cranial table unit (CMC2019-010-5), can be described using data obtained via CT scanning. These endocranial elements pertain to the median pharyngeal sinus system (sensu Dufeau & Witmer, 2015), cranial vasculature, brain endocast and certain cranial nerves. Additionally, the siphoneal tube (including a short segment in QMF59017 that is very likely related to it) rendered from the left quadrate body (CMC2019-010-6) is described in the “Quadrate” subsection above. This is the first study to provide palaeoneurological data for an extinct Australasian crocodylomorph. Prior, a preliminary study based on CT scan data has been attempted by Willis, Robinson & Kemp (1995), but they did not describe any endocranial features. Readers are encouraged to use the interactive 3D PDF file provided as supplementary to this paper in tandem with the following descriptions.

**Brain endocast**

Only small portions of the brain endocast could be digitally rendered (Figs. 31 and 32) from the cranial table (CMC2019-010-5) and the basicranial (QMF59017) units. Unfortunately, none of the rendered portions provide substantial morphological information on the brain endocast; regardless, a description for what could be segmented is still given, for completeness sake. As reiterated by many authors before (Jerison, 1973; Hopson, 1979; Franzosa, 2004; Witmer et al., 2008; Kley et al., 2010; Jirak & Janacek, 2017), we also note that the brain endocasts of crocodylomorphs, like in other reptiles, do not reflect the precise morphology of the soft tissue brains, as the brain itself is enveloped by the thick dura mater and dural venous sinuses that contribute substantially to the contours and proportions of the endocast.

The cranial table unit (CMC2019-010-5) allows for only a partial segmentation of the telencephalic region of the prosencephalon (forebrain), specifically the cerebral hemispheres and olfactory tract endocasts (Fig. 31B). Neither the cerebral hemispheres nor the olfactory tract endocasts are complete, as the ventral section of the former and its posterior section leading to the mesencephalic (midbrain) region could not be reconstructed. Similarly, the olfactory tract endocast preserves approximately half of its length, and the olfactory bulb endocasts are not present. As digitally rendered, the dorsal half of the cerebral hemispheres endocast has a roughly dome-shaped convex dorsal surface and attains a width of ~34.5 mm. The dorsal longitudinal dural venous sinus that occupied the space dorsal to the telencephalon is not distinguishable on the endocast, but its presence can nonetheless be inferred (see Witmer et al., 2008). Continuing anteriorly from the cerebral hemispheres, the endocast begins to taper gradually as the olfactory tract extends. The maximum width attained at the cerebral hemispheres-olfactory tract endocasts junction is ~21 mm. The full preserved length of the olfactory tract endocast is ~38 mm and its minimum width is ~10–11 mm, the latter parameter corresponding with the width of the olfactory tract trough as bounded by the descending processes of the frontal (see “Frontal” subsection above).

The hypophyseal fossa (along with its associated cavernous dural venous sinuses) endocast, part of the diencephalic region of the prosencephalon, is the only impression of a brain element that could be digitally segmented from QMF59017 (Figs. 32B and 32D). Since the endocast of the hypophyseal fossa is incomplete, it is rendered only at its posterior portion. As digitally rendered, the fossa’s maximum dorsoventral height is ~20 mm, maximum transverse width is ~16 mm and maximum anteroposterior length is ~7 mm.

**Cranial nerve and vasculature canals**

The canals of several cranial nerves are still present in the ‘Geoff Vincent specimen’, all within QMF59017. Their related foramina were described previously in the paper (see “Otoccipital” subsection above).

**Abducens nerve canals**

The canals for the abducens nerves (cranial nerves VI; Figs. 32B and 32D) are the smallest of the rendered nerve canals, excavating the anterodorsal half of the basisphenoid. The abducens nerves are paired, although of the two only the right canal could be fully rendered—the canal on the left could not be traced through its entire length, and thus only its posterior and anterior-most segments are presented. The abducens nerve canals are quite narrow (~2 mm) and only marginally longer (~14 mm) than those of the posterior hypoglossal nerve canals. The abducens canals are closest to each other posteriorly from whence they commence yet diverge laterally from each other as they continue anteriorly. As evident from the complete right abducens canal, in addition to the lateral curvature, the canal is also curved slightly dorsally. They flank the hypophyseal fossa dorsolaterally.

**Common canal for cranial nerves IX-XI and their accompanying vessels**

The largest cranial nerve canal that could be segmented from the *P. vincenti* holotype specimen is the metotic, or the common canal for cranial nerves IX, X and XI (i.e., the glossopharyngeal, vagus and accessory nerves, respectively) and their accompanying vessels (Figs. 32B and 32D). Only the canal(s) on the right side allowed segmentation, as the left descending pillar of the otoccipital is missing the section (metotic canal) where these nerves and associated vessels intruded the element. The right metotic canal has a length of ~44 mm and thickness (cross-sectional width) of ~8 mm and is oriented such that it offshoots posterolaterally and slightly ventrally from the braincase floor. Beginning from the anterior section of the metotic canal, it is noticeable that there are three branches. The first and anteromedial-most branches correspond with two anteromedial foramina on the descending pillar of the otoccipital (Figs. 24C and 24D). The smaller branch of the first two is short (~7 mm) and merges posteriorly with the rest of the trunk after a short distance. The third and most prominent outgrowth is that of the canal for the tympanic branch of the glossopharyngeal and vagus nerves. The tympanic branch is curved dorsolaterally, having a length of ~16 mm and thickness of ~4 mm. Ultimately, the metotic canal expands mediolaterally at its posterior and exits on the occiput through the metotic foramen on the otoccipital (Figs. 24A, 24B, 25C and 25D), described above (see “Otoccipital” subsection).

**Hypoglossal nerve canals**

The canals for the hypoglossal nerves (cranial nerves XII) are paired in that there are anterior and posterior (Figs. 32B and 32D). The hypoglossal canals on each side offshoot lateroventrally from the braincase floor.

The anterior hypoglossal nerve canals are set between the metotic canal anteriorly (specifically on the right side, for where the only metotic canal could be segmented) and posterior hypoglossal canals posteriorly. The anterior canals are long (~25 mm for the more complete right canal and ~20 mm for the left) and narrow (~2 mm in cross-sectional thickness). Since the right anterior canal is more complete, its exit can be traced on the occiput through a small foramen immediately medial to the metotic (described above, see “Otoccipitals” subsection; Figs. 24A, 24B, 25C and 25D). The posterior hypoglossal canals are the posterior-most cranial nerve canals. Both posterior hypoglossal canals are complete, with their associated foramina on the otoccipitals fully preserved as well. The posterior canals are much shorter (~13 mm), but also thicker (~5 mm) than the anterior ones.

**Cerebral carotid vasculature canals**

In life, the cerebral carotid vasculature canals were coursed by the cerebral carotid arteries and veins (Porter, Sedlmayr & Witmer, 2016). In QMF59017 the two cerebral carotid canals are extensive, and both have a thickness of ~7 mm, however, they could not be traced through their entire lengths (Figs. 32B and 32D). Of the two, the right canal is more complete. The canals can begin to be traced anteriorly from the anterior carotid foramina located within the hypophyseal fossa (or, bifurcating from the posterior of the hypophyseal fossa endocast). Then, they stretch posteriorly in a straight line for a very brief length of ~6 mm, after which immediately diverge from each other by curving sharply dorsolaterally. Their anterior segments are slightly undulating along their lengths. Each canal exits the basisphenoid through a large foramen on both sides of the bone and would have continued over to the otoccipital. It is these intermediate segments between the basisphenoid and otoccipitals that are missing. Carving through the otoccipital is the right cerebral carotid canal, whereas on the left side, only the ventral floor of the equivalent canal is still preserved (Figs. 24, 25A and 25B). Due to its completeness, this description will continue following only the canal on the right. On the internal side of the otoccipital, two foramina for the cerebral carotid vasculature are visible—one large (~5 mm in diameter), and immediately dorsolateral to the same is a much smaller foramen (~1 mm in diameter; Figs. 24C and 24D). The smaller foramen accommodates a very short and stout, almost imperceptible branch that offshoots laterally from the rest of the canal. The posterior segment of the cerebral carotid canal has a significant posteroventral and slightly lateral orientation. Finally, the canal exits on the occiput through the posterior carotid foramen (Figs. 24A, 24B, 25C and 25D). An intriguing configuration is seen at the anterior commencement of the canals, where the right canal & anterior carotid foramen abuts and overlies the left—commonly, the anterior cerebral carotid foramina, and hence anterior segments of the cerebral carotid canals, are more-or-less symmetrical and situated laterally to each other.

**Cranial pneumaticity**

As for the cranial nerves and vasculature, the best source of information regarding the cranial pneumaticity in the ‘Geoff Vincent specimen’ is the basicranial unit (QMF59017). Digitally rendered from within the basicranium were the median pharyngeal sinus, and the basioccipital and basisphenoid diverticula (Figs. 32B and 32D). As noted above (see “Basioccipital” subsection), the pharyngotympanic foramina are not detectable externally on QMF59017; likewise, the canals for the pharyngotympanic tubes of the pharyngotympanic sinus could not be traced in the CT scanned data either.

The median pharyngeal sinus (sensu Dufeau & Witmer, 2015; Figs. 32B and 32D) is a subvertical canal found between the basioccipital-basisphenoid contact. This median canal is quite long (~71 mm) but somewhat narrow (~8 mm). More dorsally, the median canal becomes confluent with a short anterior canal (i.e., the basisphenoid diverticulum) and two posterolateral canals (i.e., the basioccipital diverticula). Also, the median pharyngeal canal is partially filled with matrix, which is the only part of QMF59017 affected by such residue.

The anterior canal, or the basisphenoid diverticulum (sensu Dufeau & Witmer, 2015; =basisphenoid branch of the median canal sensu Owen, 1850; Figs. 32B and 32D) is oriented anterodorsally, and is undivided for a short length before it bifurcates into two lateral branches. The two lateral branches of the basisphenoid diverticulum diverge laterodorsally and are set lateral to the anterior sections of the cerebral carotid vasculature canals. At their dorsal most points, the two branches of the basisphenoid diverticulum additionally divide into two stout branches, one anterior and a posterior. The posterior branches exit the basisphenoid through two small communicating ostia between them and pharyngotympanic sinus that open immediately anterior to the exits of the cerebral carotid vasculature canals from the basisphenoid (Figs. 25A and 25B).

Posterodorsally, the median pharyngeal sinus canal/median pharyngeal tube is confluent with the prominent basioccipital diverticula (sensu Dufeau & Witmer, 2015; =rhomboidal sinuses sensu Owen, 1850; Figs. 32B and 32D). The basioccipital diverticula are relatively bulky and somewhat subvertical. These diverticula are located medially and slightly ventrally to the cerebral carotid vasculature canals and exit the basioccipital laterally through large communicating ostia between them and pharyngotympanic sinus (Figs. 25A and 25B). Although the rest of the pharyngotympanic sinus could not be digitally reconstructed, the communication between the pharyngotympanic sinus and the basioccipital diverticula is attested by the confluency of the aforementioned ostia with the ‘trenches’ on the quadrate where the pharyngotympanic sinus on each side of the neurocranium would have invaded.

**Specimen referrals to *Paludirex* and species differentiation**

Amongst the crocodylian material we had available for examination, credible referral to the genus *Paludirex* was possible for just five specimens (Table 1). Only two of them (the *P. vincenti* holotype and PVM 89-1072) are commendably complete, whereas two are isolated rostral pieces and one is a dentary fragment. Regardless, four of the key specimens share the distinctive arching at the anterior premaxillary alveolar processes (character 226, state 1; Fig. 11). In addition to this trait, a few other premaxillary features can be used to support the referral to *Paludirex*, such as the relative size and arrangement of the alveoli, with the first and second premaxillary alveoli being in-line and separated from each other by a considerable gap (Figs. 3C, 3D, 5C, 5D, 10 and 30). The assignment of the dentary fragment QMF17066 to *P. gracilis* is by virtue of association with the holotype specimen (QMF17065) for that species.

Willis (1995) and Willis & Molnar (1997a) recognized that there is more than one species in their concept of the genus *Pallimnarchus*. We concur that their species *Pallimnarchus gracilis* is specifically distinct from any of the specimens they referred to *Pallimnarchus pollens*, including ‘Geoff Vincent’s specimen’. The diagnosis for *Pallimnarchus pollens* by Molnar (1982b) was exclusively based on mandibular features, and so the amended generic diagnosis by Willis & Molnar (1997a) also included several mandibular characters. Hence, their differential diagnosis between *Pallimnarchus pollens* and *Pallimnarchus gracilis* listed 10 morphological features, with only three of them being non-mandibular. Herein, only one *Paludirex* specimen is mandibular, but that fragment offers frustratingly little morphological insight. At present, no other mandible or mandibular elements previously placed in *Pallimnarchus* can be assigned to *Paludirex* (see “On the mandibular elements previously regarded as *Pallimnarchus*” subsection below), since the diagnosis for *Paludirex* is based on the cranial material. The cranial traits used by Willis & Molnar (1997a) to differentiate *Pallimnarchus pollens* from *Pallimnarchus gracilis* were based on the formers more robust proportions, a bulbous premaxilla, and proportionally smaller supratemporal fenestrae. As the more robust proportions and bulbous premaxillae are in accord with each other they can be analyzed simultaneously. This difference was illustrated by Willis & Molnar (1997a) in their figure 3 (also figure 13.2 in Willis, 1995). In Fig. 33 we follow-up on that comparison where we again emphasize the proportional differences between *P. gracilis* and *P. vincenti*. It may be tempting to interpret this robustness disparity between the *P. vincenti* premaxillae and the more slender QMF17065 premaxilla as intraspecific variation. Intraspecific variability is widely acknowledged in extant crocodylians and can be a resultant of several factors. One factor can be age difference, with gerontic individuals typically being larger than younger members of their species even if the younger and smaller individuals are nonetheless sexually mature adults. That ties with the continual growth in combination with the longevity experienced by living crocodylians (Cott, 1961; Grigg & Kirshner, 2015). Additionally, intraspecific sexual dimorphism in adult crocodylians is expressed in size differences that are commonly biased towards males being the much larger sex (Fitch, 1981; Hall & Portier, 1994; Platt et al., 2009, 2011; Barrios-Quiroz, Casas-Andreu & Escobedo-Galván, 2012; Warner et al., 2016; Grigg & Kirshner, 2015; Edwards et al., 2017). However, the difference observed between *P. gracilis* and *P. vincenti* does not conform with the variability seen in extant taxa. The specimens referred to *P. vincenti* have a consistent premaxillary depth that is between 48.9% (CMC2019-010-2) and 53% (QMF11626) the collective premaxillary width, contrasting with the premaxillary depth of 28.3% the collective premaxillary width in the *P. gracilis* holotype specimen (see Table 5 for measurements). This proportional disparity between *P. gracilis* and *P. vincenti* is well outside the range of intraspecific variation we calculated for some extant *Crocodylus*, as our results indicate a premaxillary depth vs collective premaxillary width range between 40–52% in *C. johnstoni*, 40–44% in *C. novaeguineae*, and 39–48% in *C. porosus* (see Table S2.3). Therefore, our data does not support any assumptions that the discordance between the Pliocene *Paludirex* from south-eastern Queensland and the Pleistocene *Paludirex* from northern Queensland stem from intraspecific variation and/or sexual dimorphism.

**Table 5:** Premaxillary depth and width measurements in three specimens of *Paludirex* gen. nov.

Specimen	Premaxilla depth (mm)	Premaxilla width (mm)	Collective premaxillary width (mm)
CMC2019-010-2^v^	~110	~112.5	~225
QMF11626^v^	~105	~99	~198
QMF17065^g^	~51	~90	~180

**Note:**

Out of the three specimens, only the holotype of *Paludirex vincenti* gen. et sp. nov. preserves both premaxillae to allow direct measurement of their collective width. However, since the premaxillae of a single individual are symmetrical and have equal dimensions, the collective premaxillary width for the other two specimens was calculated by multiplying the width of the only preserved premaxilla by two. The *Paludirex vincenti* specimens are indicated with a^v^, whereas the *Paludirex gracilis* gen. et comb. nov. holotype is indicated with a^g^.

Specimen PVM 89-1072 is undoubtably referable to *Paludirex*, although determining its specific assignment is a bit problematic. Previously, Willis & Molnar (1997a) referred PVM 89-1072 to *Pallimnarchus gracilis*. As can be gleaned from Fig. 11C, specimen PVM89-1072 appears to be proportionally less robust than *P. vincenti*, and as such it could be referred to *P. gracilis*. However, the issue here lies in the fact that the concrete PVM 89-1072 is firmly embedded in largely obscures its real rostral depth. It is possible that PVM 89-1072 has also experienced taphonomic dorsoventral compression. Due to these circumstances, the proportional differences necessary to differentiate PVM 89-1072 from either *P. gracilis* or *P. vincenti* cannot be estimated accurately. This leads to the third and final cranial feature proposed by Willis & Molnar (1997a) used to distinguish *Pallimnarchus gracilis* from *Pallimnarchus pollens*—different sizes of the supratemporal fenestrae. Unfortunately, this is another part of the PVM 89-1072 cranial table that is troublesome in interpreting correctly, especially because the dorsal surface of the cranium is completely hidden from view. The only assessment possible of the supratemporal fenestrae of PVM 89-1072 is in (the only exposed) ventral aspect. The right supratemporal fenestra of PVM 89-1072 has sustained significant damage, while the left fenestra appears to be more complete. Nevertheless, only the lateral margin of the left supratemporal fenestra is complete and comfortably exposed to allow a reliable measurement of ~33 mm. When measured in ventral view, the left supratemporal fenestra of the *P. vincenti* holotype (CMC2019-010-5) has a length of ~29 mm, just 4 mm less than in PVM 89-1072. This is too small of a difference to be regarded as a valid differentiating feature between species. Ultimately, we cannot confirm nor deny that PVM 89-1072 has overall proportionally larger supratemporal fenestrae than the ‘Geoff Vincent’s specimen’ because the fenestrae in the former are not complete and are difficult to measure due to the way the specimen is currently placed in. Other than the above discussed features, we could not identify any other characters in PVM 89-1072 that distinguish it from the *P. vincenti* holotype. To err on the side of caution, we provisionally designate PVM 89-1072 as *Paludirex* cf. *P. vincenti*, pending a more detailed study where the specimen would preferably be removed from its concrete confines, or is CT scanned.

**Phylogeny**

**Results from the phylogenetic analyses**

The taxonomic relationships of *Paludirex vincenti* were tested in two separate phylogenetic analyses based on a novel matrix that consists of 228 morphological characters and 141 OTUs. In both analyses, 19 out of the 228 characters were treated as ordered (characters 21, 39, 49, 50, 54, 55, 82, 83, 89, 118, 125, 137, 142, 150, 160, 176, 202, 223 and 224). The goniopholidid crocodyliform *Anteophthalmosuchus epikrator* Ristevski et al., 2018 was selected as the outgroup taxon in the matrix. The first analysis was run under a ‘traditional’ equal weighting (EW) principal search methodology, whereas the second analysis implemented the implied weighting (IW) methodology. *Paludirex gracilis* was not included as a separate OTU because the two specimens referred to that species are insufficiently complete to allow for a meaningful scoring. Out of the 228 morphological characters, *P. vincenti* could be scored for 77 (i.e., 33.77%) characters in total. Besides *P. vincenti*, the matrix also includes 12 additional extinct Australian crocodyliform OTUs. Details on the taxon matrix and character dataset are given in the Supplemental Document S1 to this paper, with the raw data of the matrix (in NEXUS format) and results (in native .tnt formats) also available as supplements.

**Figure 34:**
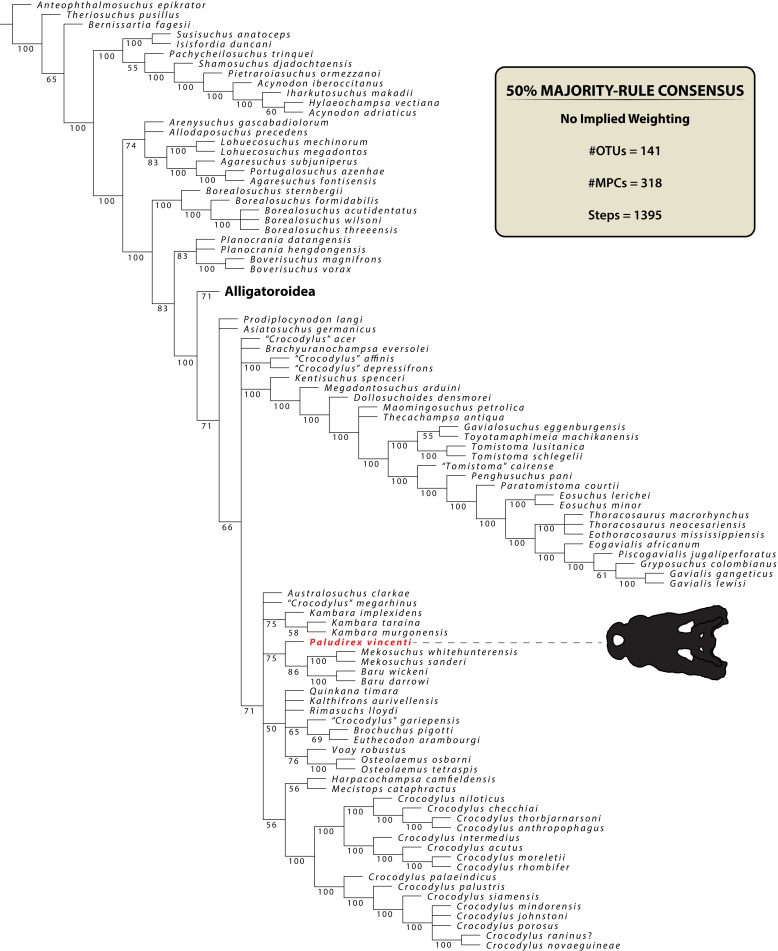
Reduced 50% majority-rule consensus topology from the first phylogenetic analysis, run without using the Implied Weighting method. See Supplemental Document S1 for more information on the recovered topology and nodal values.

The first phylogenetic analysis, performed under an EW search method, recovered 318 most-parsimonious cladograms (MPCs) with a length of 1395 steps (CI = 0.230; RI = 0.727). The resulting strict consensus topology (see Supplemental Document S1) produced a large unresolved polytomy within Crocodylia that incorporates *Paludirex* as well as the other mekosuchine OTUs. Only two mekosuchine clades were recovered in the strict consensus, one formed by the *Baru* OTUs and the second by the *Mekosuchus* OTUs. In general, nodal support is very weak. The *Baru* clade (*B. darrowi* + *B. wickeni*) is one of the few with decent nodal support (Bremer = 1; bootstrap = 66) and represented by three synapomorphies: angular-surangular suture passes broadly along the ventral margin of the external mandibular fenestra late in ontogeny (character 60, state 1); surangular with spur bordering the dentary toothrow lingually for the length of at least one alveolus (character 62, state 0); and, very large maxillary foramen for the palatine ramus of cranial nerve V (character 103, state 1). The *Mekosuchus* clade (*M. sanderi* + *M. whitehunterensis* Willis, 1997) has good nodal support (Bremer = 3; bootstrap = 61) and is characterized by four synapomorphies: non-telescoped orbits, with the frontal having broadly convex orbital margins (character 137, state 3); frontal bears prominent midsagittal crest between orbits (character 187, state 1); maxilla contributes to the orbital margin (character 204, state 2); and, adult skull length is less than 300 mm (character 225, state 1). In this analysis, six autapomorphies were identified for *P. vincenti*: medial parietal wall of supratemporal fenestra bearing foramina (character 154, state 1); significant ventral process of the quadrate on lateral braincase wall (character 168, state 0); quadrate with small, ventrally reflected medial hemicondyle (character 181, state 0); large sculpture pits on cranial table developed anterior to the supratemporal fenestrae and spread over the frontal-parietal junction, with minimal or no involvement of the postorbital (character 211, state 1); alveolar processes of the premaxillae arched (character 226, state 1); and, occipital lamina of the supraoccipital oriented posteroventrally (character 228, state 1). The 50% majority-rule consensus topology (Fig. 34 and Supplemental Document S1) is much more resolved than the one in the strict consensus, although the relationships within Crocodyloidea, particularly among Mekosuchinae, are still unclear. The 50% majority-rule consensus depicts a clade formed by *P. vincenti* as the sister taxon to a clade comprised by *Baru* + *Mekosuchus*. The three *Kambara* OTUs form a monophyletic clade but are independent of the other mekosuchines, as is *Australosuchus clarkae* Willis & Molnar, 1991. The 50% majority-rule consensus found *H. camfieldensis* as a sister taxon to *Mecistops cataphractus* (Cuvier, 1824), while *Kalthifrons aurivellensis* Yates & Pledge, 2016 and *Q. timara* are located in a polytomy within Osteolaeminae. Intriguingly, one of the few monophyletic clades recovered in our strict consensus topology (and in the 50% majority-rule consensus; see Fig. 34 and Supplemental Document S1) consists of Gavialidae, Tomistominae and ‘thoracosaurs’. However, the nodal support for the recovered Gavialoidea clade is extremely poor (Bremer = 0; bootstrap = <50%). The vast majority of published phylogenetic analyses based exclusively on morphological data have recovered Gavialidae in a basal position within Crocodylia, while Alligatoroidea and Crocodyloidea (including Tomistominae) are usually found forming a separate clade (=Brevirostres; Norell, 1989; Brochu, 1999, 2003; Salisbury et al., 2006; Salas-Gismondi et al., 2016; Jouve, 2016). In contrast, results from molecular data are in opposition to those derived from morphological datasets alone by suggesting that *Gavialis* Oppel, 1811 and *Tomistoma* Müller, 1846 are actually closely related (Densmore, 1983; Densmore & Owen, 1989; Hass et al., 1992; Gatesy et al., 2003; McAliley et al., 2006; Willis et al., 2007; Oaks, 2011; Pan et al., 2020). In recent years, several studies that performed analyses combining morphological and molecular data have managed to recover topologies that are in agreement with molecular studies (Gold, Brochu & Norell, 2014; Lee & Yates, 2018; Iijima & Kobayashi, 2019). The study done by Sookias (2020) produced a cladogram after morphology-based analyses where *Gavialis* + *Tomistoma* were recovered as sister taxa (although no OTUs of extinct crocodylians were factored in that study). As such, our results, using an EW search method, are a rare example of finding a monophyletic Gavialoidea that includes tomistomines based on morphological data alone (albeit with the inclusion of ‘thoracosaurs’). Admittedly, these results are far from offering closure to the debate surrounding the relationships of *Gavialis*, *Tomistoma* and their extinct relatives, and further research is most certainly needed on this topic, a topic that is well outside the aims and scope of this study.

**Figure 35:**
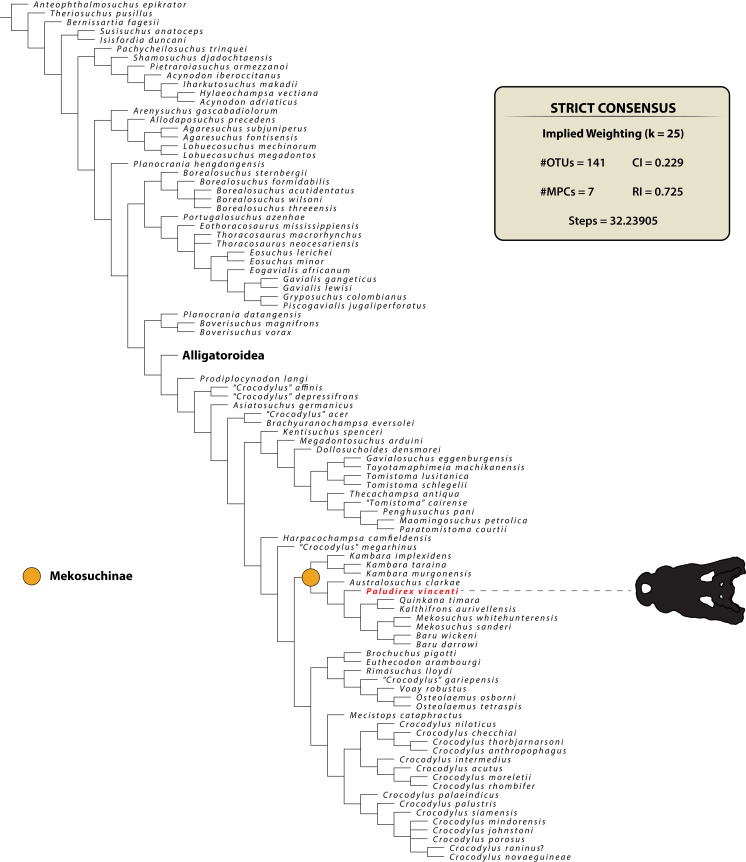
Reduced strict consensus topology from the second phylogenetic analysis, using the Implied Weighting method. See Supplemental Document S1 for more information on the recovered topology and nodal values.

Due to the poorly resolved relationships within Crocodylia, a second analysis was performed. For this analysis we employed the implied weighting (IW) method (Goloboff, 1993) with the *k* (concavity constant) value set to 25.0 (*k* = 25). Analyses performed under IW aim to lower the influence of homoplastic data and yield better resolved topologies with fewer polytomies (Jones & Butler, 2018; Herne et al., 2019; Rio et al., 2020; Sookias, 2020), and a recent study regards IW as a means to provide more accurate results (Goloboff, Torres & Arias, 2018). Aside from the IW method, all other search parameters in the analysis were exactly the same as in the previous (see “Phylogenetic analyses” in the “Materials and Methods” section above). The results from the analysis utilizing the IW method recovered 7 MPCs with a length of 32.23905 steps (CI = 0.229; RI = 0.725). The strict consensus topology (Fig. 35) is highly resolved and largely congruent with most other published cladograms that have predominantly eusuchian taxa (Brochu, 1999; Salisbury et al., 2006; Narváez et al., 2015, 2016; Mateus, Puértolas-Pascual & Callapez, 2019; Rio et al., 2020). The nodal support is again quite weak throughout the cladogram (see Supplemental Document S1). Unlike the previous analysis, we failed to recover a monophyletic Gavialoidea that includes tomistomines; this time, the topology finds Tomistominae within Crocodyloidea whereas Gavialoidea (Gavialidae + ‘thoracosaurs’) is in a basal position in Crocodylia. All of the Cenozoic crocodylians from Australia are recovered within Crocodyloidea, and with the exception of *H. camfieldensis* and the Australasian *Crocodylus*, within a monophyletic Mekosuchinae. However, Mekosuchinae has very weak nodal support (Bremer = 0; bootstrap = <50%). *Kambara* is the basal-most taxon within the clade, followed by *A. clarkae* and *P. vincenti* in more derived positions. *Paludirex vincenti* is found firmly within Mekosuchinae as the sister taxon to the clade formed by representatives of Mekosuchini ([*Q. timara* + *K. aurivellensis*] + [*Mekosuchus* + *Baru*]). The mekosuchine sub-clade Mekosuchini was formally proposed by Salisbury & Willis (1996) as the clade comprised by *Baru*, *Mekosuchus*, *Quinkana* and *Trilophosuchus*. Also, the Fijian taxon *Volia athollandersoni* Molnar, Worthy & Willis, 2002 was subsequently recovered within Mekosuchini in the analyses by Molnar, Worthy & Willis (2002) and Lee & Yates (2018). However, we currently refrain from assigning *K. aurivellensis* as a definitive member of Mekosuchini due to the mutable position of some taxa within the clade, and more analyses are needed to resolve the composition of Mekosuchinae as a whole. Furthermore, since other taxa assigned to Mekosuchini (such as *Trilophosuchus*, *Volia* Molnar, Worthy & Willis, 2002 and the remaining species of *Mekosuchus* and *Quinkana*) were not included in this iteration of the matrix, it would be prudent to comment on that sub-clade when all named mekosuchins are accounted for. In this analysis, *P. vincenti* is again characterized by the same six autapomorphies as in the previous one.

**Phylogenetic implications**

Based on the results from the phylogenetic analyses (Figs. 34 and 35; see also Supplemental Document S1), *P. vincenti* is a crocodyloid and a member of Mekosuchinae. Its position within the clade is more derived than that of *Kambara* and *Australosuchus*, and it clusters closer to mekosuchines such as *Baru* and *Mekosuchus*. Whilst clearly possessing a unique combination of morphological features that justifies its generic and specific distinction, *P. vincenti* does share certain characteristics with other Australian crocodylians (for additional details see the “Description” section above). For example, the small supratemporal fenestra with a roughly D-shaped dorsal outline (character 207, state 5) of *P. vincenti* is most similar, although obviously not identical, to those of *B. wickeni* and *M. sanderi* (Figs. S1.14F, S1.14G and S1.14H).

A pair of shallow pits that differ from the usual cranial ornamentation and located between the orbits and supratemporal fenestrae was considered a diagnostic feature of *Kambara*, and now *P. vincenti* is the second taxon where this feature has been identified in. As described above for *P. vincenti* (see “Parietal” subsection), the pits anterior to the supratemporal fenestrae are found at the frontoparietal sutural junction (character 211, state 1). Unlike *P. vincenti* though, the pits in *Kambara* cross-over to the postorbitals as well (Willis, Molnar & Scanlon, 1993; Salisbury & Willis, 1996; Holt, Salisbury & Willis, 2005; Buchanan, 2009; character 211, state 2). Whether these pits are homologous between *Paludirex* and *Kambara* is unclear due to the small sample of specimens; it should be noted that a *Kambara implexidens* paratype specimen (QMF29663) lacks such pits, to which Salisbury & Willis (1996) suggested that their presence or absence may be influenced by ontogeny, sex and/or diet.

However, *P. vincenti* also shares a handful of peculiar features only with *Harpacochampsa* among known Australian crocodylians—the ventrally extending pterygoid process of the quadrate on the lateral braincase wall (character 168, state 0) as well as a nuchal crest on the supraoccipital that is not laterally delimited by concavities. According to Brochu (1997), the former condition occurs in alligatoroids, gavialoids, and some basal eusuchians, while it is absent in crocodyloids except for *Prodiplocynodon langi* Mook, 1941. A ventrally reflected quadrate-pterygoid suture laterally on the braincase was also observed in the holotype specimen of *Harpacochampsa camfieldensis* (NTM P87106-1; see Figs. 6D and 6E in Megirian, Murray & Willis, 1991).

Another feature of interest can be seen on the left descending process of the parietal in CMC2019-010-5, which is pierced by four conspicuous foramina (character 154, state 1; Fig. 23; see “Parietal” subsection above). According to Brochu (1997), this peculiar feature has been previously recognized in certain caimanines. Amongst Australian crocodylians, the *P. vincenti* holotype specimen is the first reported about this trait.

## Discussion

### Possible synonymy between *Paludirex* and *Pallimnarchus*

Considering the prevalent usage and familiarity of *Pallimnarchus pollens* within the literature on Australian fossil crocodylians, its importance as a name cannot be overstated. As such, the decision to declare it a *nomen dubium* was not made lightly. Because the novel binomen *Paludirex vincenti* is now given to some specimens that were previously treated as *Pallimnarchus pollens*, it is not outside the realm of possibility that *Paludirex vincenti* could become a junior synonym of *Pallimnarchus pollens* provided certain criteria are met. Initially, that would depend on finding the rest of the missing *Pallimnarchus pollens* lectotype, QMF1149, and detecting at least one unambiguous autapomorphy on it. This would make *Pallimnarchus pollens* a valid nomen again. The (potential) synonymy of *Paludirex vincenti* with *Pallimnarchus pollens* would then hinge on the discovery of a specimen that preserves the mandible with characteristics diagnostic of *Pallimnarchus pollens* along with associated cranial material that is diagnostic of *Paludirex vincenti*. If those criteria are met, then *Pallimnarchus pollens* would be a valid name and *Paludirex*, specifically *Paludirex vincenti*, should be treated as its junior synonym.

However, it will also be necessary to compare QMF1149 not only with *P. vincenti*, but with the possible *Crocodylus* sp. hinted at by Yates & Pledge (2016) and Yates (2019), as well as another taxon that is discussed below (see “Large, non-ziphodont crocodylians from the Pliocene of Chinchilla—more than just *Paludirex vincenti*?” subsection below). It seems very likely that there is more than one broad-snouted crocodylian from the Pliocene of south-eastern Queensland, and it is entirely possible that the lectotype of *Pallimnarchus pollens* originated from a different species than the one we are naming *Paludirex vincenti*.

### Large, non-ziphodont crocodylians from the Pliocene of Chinchilla—more than just *Paludirex vincenti*?

**Figure 36:**
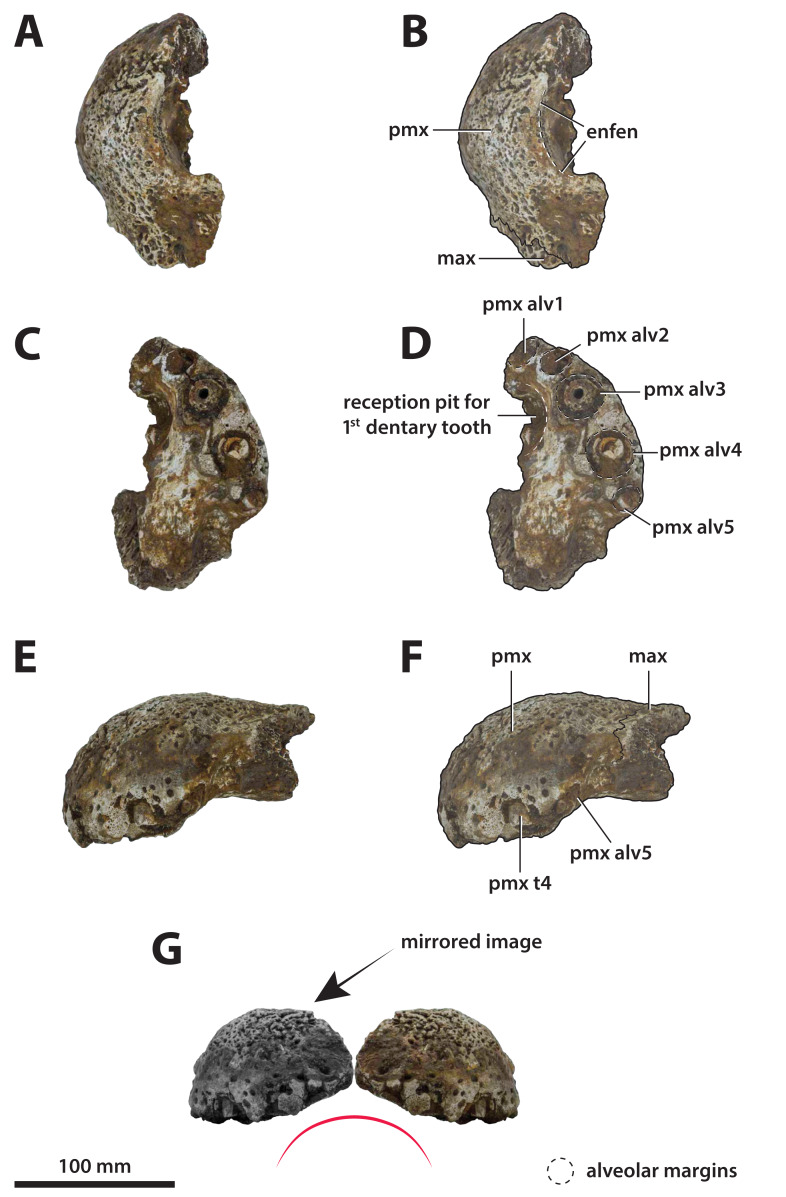
*Paludirex*? sp. nov., QMF1154, left premaxilla and fragment of maxilla. Dorsal view of the specimen (A) non-annotated photograph, and (B) annotated photograph. Ventral view of the specimen (C) non-annotated photograph, and (D) annotated photograph. Left lateral view of the specimen (E) non-annotated photograph, and (F) annotated photograph. (G) Premaxilla in anterior view, with the indicated grayscale photograph mirrored in order to aid comparison. The curved red line in (G) indicates the anterior arching of the premaxilla. Abbreviations: enfen, external narial fenestra; max, maxilla; pmx, premaxilla; pmx alv, premaxillary alveolus; pmx t, premaxillary tooth.

In the past, it was assumed that there are only two crocodylian taxa from the Chinchilla Local Fauna—the non-ziphodont *Pallimnarchus* and the ziphodont *Quinkana* (Louys & Price, 2015). This notion has been challenged in recent years by Chiotakis (2018, 2019), where, based on dental morphometrics, the possibility for a third taxon from that fauna was suggested. Aside from *Pallimnarchus pollens*—herein regarded to be a *nomen dubium*—*Paludirex vincenti* stands as the only named non-ziphodont crocodylian from the Chinchilla Local Fauna. However, a partial rostral piece, QMF1154, strongly suggests the presence of another non-ziphodont crocodylian from the Pliocene of Chinchilla. QMF1154 (Fig. 36) is an almost complete left premaxilla still in articulation with an anterior fragment of its left maxilla. This specimen was briefly discussed by Molnar (1982b), Willis & Molnar (1997a), and Louys & Price (2015). Prior to this study, QMF1154 was figured by Willis & Molnar (1997a) in their Fig. 5B (mislabeled in the figure caption as specimen QMF11626). In his paper, Molnar (1982b, p. 662*)* concluded that QMF1154 “…probably derives from *Pallimnarchus*”, an opinion later agreed on by Willis & Molnar (1997a), although the latter did not clarify as to which of their two *Pallimnarchus* species they intended to refer the specimen (though probably *Pallimnarchus pollens* based on the caption for their Fig. 5B). This specimen was also listed by Lees (1986) and Louys & Price (2015), with both referring it to *Pallimnarchus pollens*. Based on its museum record, QMF1154 comes from the Chinchilla Sand, the same unit as the *P. vincenti* holotype.

The QMF1154 premaxilla resembles *Paludirex*, mostly *P. vincenti*, in a couple of aspects such as in the dorsoventral depth (Figs. 36E and 36F) and the arching of the premaxillary alveolar process (Fig. 36G). Also, the premaxilla of QMF1154 is shallowly sloping anteriorly when observed in lateral aspect, and all its alveoli are circular in outline. The premaxillary depth of QMF1154 is ~85 mm and its width ~88 mm, which results with a premaxillary depth that is ~48% the collective premaxillary width—proportionally, this is virtually identical to *P. vincenti* (CMC2019-010-2). As indicated by its premaxillary and alveolar dimensions (Table 6), QMF1154 belonged to a very similarly sized individual as the *Paludirex gracilis* holotype (QMF17065). In fact, the QMF17065 premaxilla is marginally larger than QMF1154 and yet noticeably less robust (see Tables 4–6 of this paper, as well as Fig. 5 in Willis & Molnar, 1997a). Therefore, a referral for QMF1154 to *P. gracilis* can be eliminated. But, assigning QMF1154 to *P. vincenti* would be misguided as well.

**Table 6:** Premaxillary alveoli dimensions of QMF1154.

Alveolus position	Mesiodistal length (mm)	Labiolingual width (mm)
1st premaxillary	~16	–
2nd premaxillary	~14	~17
3rd premaxillary	~27	~25
4th premaxillary	~27	~27
5th premaxillary	~15	~15

**Note:**

The parameter that could not be reliably measured due to a substantially damaged or missing alveolar rims has omitted data (indicated with a dash).

**Figure 37:**
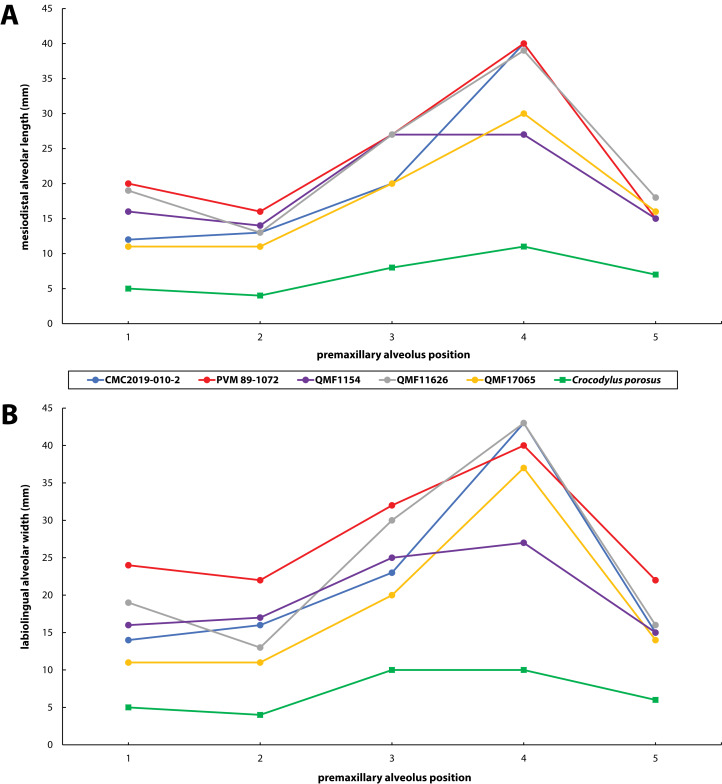
Premaxillary alveoli size comparisons between QMF1154, four *Paludirex* gen. nov. specimens and *Crocodylus porosus* Schneider, 1801. Graphs showing the (A) mesiodistal length, and (B) the labiolingual width of the premaxillary alveoli. Notice how QMF1154 has a less pronounced size disparity than *Paludirex* between its premaxillary alveoli, particularly the 3rd and 4th alveoli.

The differences between QMF1154 and the *Paludirex* premaxillae described in this study become most apparent in palatal view (Figs. 36C and 36D). The 1st premaxillary alveolus of QMF1154 is set anterior to the 2nd and there is only a small interalveolar gap between them. This deviates from the condition present in *Paludirex gracilis* (QMF17065) and *Paludirex vincenti* (CMC2019-010-2 and QMF11626; also, PVM 89-1072) where the first two premaxillary alveoli are almost in line with each other and separated by a more substantial interalveolar gap. Moreover, the 3rd premaxillary alveolus in QMF1154 has the same diameter as the 4th, in contrast to the much larger 4th premaxillary alveoli in *Paludirex* (compare Figs. 3, 5 and 10 with Fig. 36, and Tables 2–4 with Table 6; also, Fig. 37). In dorsal view (Figs. 36A and 36B), the posterior margin of the external narial fenestra of QMF1154 is being close to transverse (already remarked by Molnar, 1982b), and not gently curved posteromedially like it is in *Paludirex* (CMC2019-010-2, QMF11626 and QMF17065).

Given the obvious morphological disparity between QMF1154 and the premaxillae of both *P. gracilis* and *P. vincenti*, it is likely that QMF1154 is at least specifically distinct from the two. However, whether QMF1154 represents a third species of *Paludirex* or if it represents a distinct genus is more difficult to solve. Our generic diagnosis for *Paludirex* is limited to characters of the premaxilla; all non-premaxillary cranial features are known solely from *P. vincenti* and for the time being must remain exclusive to that species, awaiting future discoveries of more complete *P. gracilis* specimens. Being a virtually complete premaxilla, QMF1154 can be analyzed in the context of the generic diagnosis for *Paludirex*. Of the five mutual diagnostic features among *P. gracilis* and *P. vincenti*, QMF1154 shares only three: the dorsally arched premaxillary alveolar processes, the shallowly sloping anterior profile of the premaxilla, and the circular to sub-circular premaxillary alveolar outlines. Granted, the last two characters are widespread across Crocodylia, and on their own inform extremely little of the specimen’s taxonomic affinities. On the other hand, dorsally arched premaxillary alveolar processes have never been reported for any crocodylian before, and are presently unique to *Paludirex*. However, a currently undescribed crocodylian from the Miocene of Australia (NTM P6319; *Baru* sp. nov. sensu Yates, 2017; *Baru* n. sp. ‘Alcoota’ as in Lee & Yates, 2018) also shares a similar arching of the premaxilla (Adam M. Yates, 2019, personal observations). Based on its general morphology, QMF1154 appears more similar to *Paludirex* than to any currently described species of *Baru*. Nonetheless, confident generic identification for QMF1154 is currently not possible. Pending the description of NTM P6319, we provisionally designate QMF1154 as *Paludirex*? sp. nov., but note that it is neither *P. gracilis* or *P. vincenti*.

The probable identification of a second broad-snouted, non-ziphodont crocodylian from the Chinchilla Sand is of great importance to understanding the reptilian in general, and crocodylian in particular, faunal composition during the Pliocene of southeast Queensland. This knowledge also has significant impact on determining the validity of the name *Pallimnarchus pollens*. Should the missing lectotype (QMF1149) of *Pallimnarchus pollens* be ever recovered and demonstrated to possess unambiguous autapomorphies, its comparisons would not be limited only to *Paludirex vincenti*, but also the taxon that QMF1154 belongs to.

### On the mandibular elements previously regarded as *Pallimnarchus*

Throughout the past century, incomplete mandibular material has been attributed to *Pallimnarchus* by various authors (De Vis, 1907; Longman, 1928; Anderson, 1937; Hill, Playford & Woods, 1970; Molnar, 1982b; Willis & Molnar, 1997a). However, in addition to being incomplete, all of those mandibular remains also have an inherent problem in that none are associated with cranial elements. A likely exception are the *Paludirex gracilis* holotype specimen QMF17065 (Figs. 5 and 11E) and QMF17066 (Fig. 6). According to Willis & Molnar (1997a), the QMF17065 premaxilla and the QMF17066 dentary fragment may belong to one individual as both were found in association. Indeed, first-hand examination shows that the preservational states and proportions of QMF17065 and QMF17066 match closely with each other (also noted by Willis & Molnar, 1997a). Unfortunately, QMF17066 is very incomplete and largely uninformative, which makes comparisons between it and other dentaries highly restricted and precarious.

In light of this revision, all mandibular material, except QMF17066, previously referred to *Pallimnarchus* are provisionally reassigned as Crocodylia indet. As discussed above, there is currently no published mandibular element that was found in association with a cranium (with exception of QMF17065 and QMF17066), and therefore it cannot be unequivocally demonstrated that any isolated mandible is referable to *Paludirex*. While proportional similarities may be inferred from some of the mandibles and the cranial material in both being from a broad-snouted crocodylian, we do not view that as sufficient evidence to justify taxonomic associations. Certainly, it is probable that some, but likely not all, of the mandibular elements previously assigned to *Pallimnarchus* are now attributable to *Paludirex*. However, until a specimen that preserves ample parts of both the mandible and cranium comes to light, we suggest that *Paludirex* be temporarily recognized from cranial elements.

### On the original cranial fragments described by De Vis (1886)

**Figure 38:**
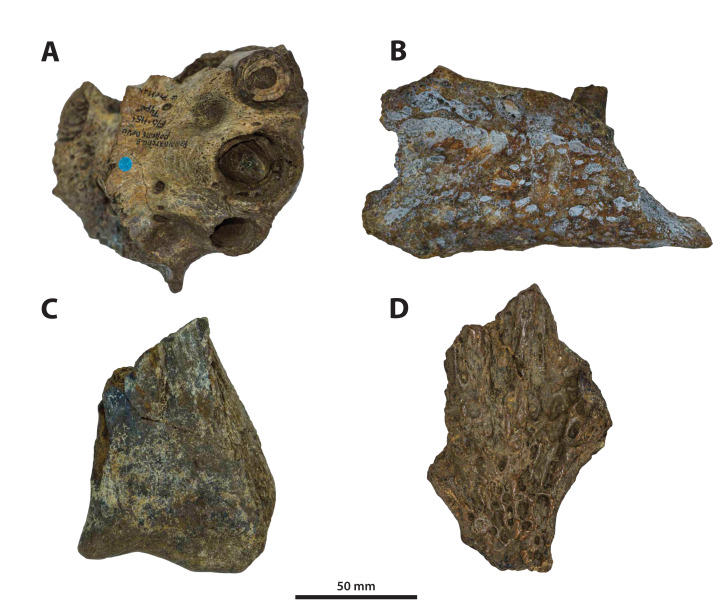
The original cranial pieces that were initially referred to *Pallimnarchus pollens* De Vis, 1886. (A) *Paludirex*? sp. nov., QMF1151, incomplete left premaxilla in ventral view. (B) *Quinkana* sp. indet., QMF1152, incomplete left jugal in lateral view. (C) Crocodylia *incertae sedis*, QMF1160, incomplete right quadrate in dorsal view. (D) Crocodylia *incertae sedis*, QMF3303, fragment of preorbital region in dorsal view. In (D), anterior is towards the top of the figure.

In his original publication on *Pallimnarchus pollens*, De Vis (1886) presented eight craniomandibular pieces and five osteoderms. Of these, only five are cranial elements: QMF1151, a partial left premaxilla (Fig. 38A; see also Pl. 13 in De Vis, 1886; Fig. 3 in Molnar, 1982b); QMF1152, an incomplete left jugal (Fig. 38B; see also Pl. 14, fig. 1 in De Vis, 1886; Figs. 11 and 12 in Molnar, 1981; Figs. 7G and 7F in Megirian, 1994); QMF1160, an isolated right quadrate body (Fig. 38C; see also Pl. 12, fig. 2 in De Vis, 1886; Pl. 2, fig. J in Molnar, 1982b); and, QMF3303, a partial preorbital region of a skull preserving small sections of the frontal, prefrontals and nasals (Fig. 38D; see also Pl. 14, fig. 2 in De Vis, 1886; Fig. 2 in Molnar, 1982b). The fifth and last piece is a fragment of a maxilla (see Pl. 10, fig. 2 in De Vis, 1886), although as remarked by Molnar (1982b), this specimen has not been located. The current taxonomic classification of these four specimens is addressed below.

The first of these pieces, QMF1151 (Fig. 38A), is a well preserved albeit incomplete left premaxilla. Besides De Vis (1886), QMF1151 was also referred to *Pallimnarchus pollens* by Lees (1986), Willis (1995) and Willis & Molnar (1997a), while Molnar (1982b) was hesitant to confidently assign it to the aforementioned. Specimen QMF1151 is a robustly built, dorsoventrally deep premaxilla with an overall morphology that is fairly comparable to that of *P. vincenti* (CMC2019-010-2 and QMF11626), but even more similar to specimen QMF1154 (see above). The 3rd premaxillary alveolus of QMF1151 has incomplete margins, although it appears slightly smaller than the 4th (diameters of ~27 mm and ~30 mm, respectively), while the 5th alveolus is the smallest (~20 mm in diameter). A small interalveolar gap separates the 3rd from the 4th alveolus, whereas the separation between the 4th and 5th alveoli is narrower. The 5th premaxillary alveolus is set on a more dorsal level relative to the 3rd and 4th alveoli. Shallow reception pits for dentary teeth are located immediately lingual to and between the 3rd and 4th and 4th and 5th alveoli, with the pit between the former being the more conspicuous one. Unfortunately, QMF1151 is missing the anterior portion of the premaxilla. All preserved alveoli are circular and as indicated by a replacement tooth within the 4th alveolus of QMF1151, it has a non-fluted enamel surface and crenulated carinae. Lastly, QMF1151 lacks a perinarial crest around its external narial fenestra. Another noticeable difference between QMF1151 and the *Paludirex* specimens described here is the transversely oriented posterior margin of its external narial fenestra, a feature of QMF1151 that was commented on by Molnar (1982b) and Willis & Molnar (1997a). A transverse posterior narial margin is present in QMF1154. Based on this comparative description, QMF1151 is most similar to QMF1154 and they could belong to the same taxon. Like QMF1154, QMF1151 is also treated as *Paludirex*? sp. nov.

Specimen QMF1152, an incomplete left jugal (Fig. 38B), was first described by De Vis (1886) as a “left malar” (De Vis, 1886, p. 187). Later, the same specimen was discussed and described by Molnar (1981) where he suggested that the jugal was most likely from a deep-snouted crocodylian, akin to “*Quinkana* or a similar form” (Molnar, 1982b, p. 660). The most recent mentions of QMF1152 were in Megirian (1994) and Louys & Price (2015) where it was designated as *Quinkana* sp. QMF1152 is derived from the Chinchilla Sand and is Pliocene in age (Molnar, 1981; Lees, 1986; Louys & Price, 2015). This partial jugal is very distinct and does not exactly match the morphology of any other jugal for a presently named Australian crocodylian, including *Paludirex vincenti*. For one, the ascending process of the jugal in QMF1152 is not as deeply inset as that of *P. vincenti* CMC2019-010-4 and the ornamentation on the lateral surface comprises sub-circular and elongated pits, contrasting with the much more rugose ornamentation present on the ‘Geoff Vincent specimen’. The jugal QMF1152 also lacks a concavity on its ventromedial surface that is diagnostic for *P. vincenti*. Additionally, the dorsal margin of the QMF1152 jugal (corresponding with the ventral margin of the orbit) is in the form of a straight, smooth and flat shelf. The most glaring feature of QMF1152 is the ornamented ventral margin of the anterior jugal process that is sharply bent from the lateral surface at a 90˚ angle (character 205, state 1) – this is very similar to a small isolated jugal, CMC2, possibly from Chinchilla Sand as well. Also, the QMF1152 jugal has similar, but not identical, morphology to that of *Australosuchus clarkae* (QMF18102), *Mekosuchus sanderi* (QMF31166), *Trilophosuchus rackhami* (QMF16856) or (as noted by Molnar, 1981) *Alligator mississippiensis* (Daudin, 1802; e.g., QMJ4850). Megirian (1994) provided comparisons between QMF1152 and *Quinkana timara* (NTM P895-19 and NTM P8697-2), demonstrating that these jugals are fairly similar to each other. Indeed, QMF1152 is most similar to the jugal of *Q. timara* although larger and slightly different from both NTM P895-19 and NTM8697-2. Based on this comparison, we agree with the other authors in treating QMF1152 as an indeterminate species of *Quinkana*.

The isolated right quadrate body (QMF1160; Fig. 38C) was first described as a “condylar portion of tympanic” (De Vis, 1886, p. 188). This specimen is neither complete nor particularly well preserved, although it does show differences in the morphology of the quadrate condylar surface when compared with the quadrate body of the ‘Geoff Vincent specimen’ (CMC2019-010-6), as the lateral condyle in CMC2019-010-6 is proportionally larger and the medial hemicondyle is more inflected ventrally (compare Fig. 28A of this paper with Pl. 12, fig. 2 in De Vis, 1886). At the moment, the taxonomic affinity of QMF1160 cannot be determined and so we provisionally designate it as Crocodylia *incertae sedis*.

The partial cranial piece QMF3303 (Fig. 38D; initially described as a “prefrontal region” by De Vis, 1886, p. 187) is another specimen with undecided affinities. The preserved region (anterior frontal process, posterior processes of the nasals and medial segments of the prefrontals; see figure 2 in Molnar, 1982b) of QMF3303 is not only relatively indistinct, but also cannot be directly compared with the ‘Geoff Vincent’s specimen’ (nor with PVM 89-1072 as the dorsal surface of that specimen is not exposed) because the equivalent portion is not ideally preserved in the latter. Thus, QMF3303 is also designated as Crocodylia *incertae sedis*.

From this review of the original cranial fragments brought up in the paper by De Vis (1886), only QMF1152 is sufficiently preserved to allow for a (likely) taxonomic assignment to the genus *Quinkana*. The remaining three specimens have inconclusive affinities, although QMF1151 has a morphology very similar to that of QMF1154 and could warrant a reevaluation in the future.

### The ‘Lansdowne snout’

A specimen that was assigned to *Pallimnarchus*—QMF1752, also known as the ‘Lansdowne snout’ – deserves a detailed inspection after the taxonomic revision given in this study. QMF1752 is best known (and figured; see Figs. 1 and 2 in Longman, 1925; Fig. 5 in Molnar, 1982b; Fig. 6 in Willis & Molnar, 1997a; Fig. 2.29 in Molnar, 2004) as an incomplete skull, preserving almost the entire cranial rostrum up to its orbital region. Described by Longman (1925) as a specimen of *Pallimnarchus pollens*, QMF1752 has had shifting taxonomic referrals for decades. The first reexamination of this specimen was done by Molnar (1982b) who viewed it as a *C. porosus* (also Lees, 1986). Later, QMF1752 was again suggested to be a species of *Pallimnarchus* (Willis, Murray & Megirian, 1990; Molnar, 1991), with its most recent designation being *Pallimnarchus gracilis* by Willis & Molnar (1997a; also, Willis, 1995 and Molnar, 2004). Longman (1925) also reported that the rostrum was accompanied by other fragments, including mandibular and postcranial elements, but according to him they “…are too incomplete to yield useful evidence.” (Longman, 1925, p. 108). The rostrum of QMF1752 is currently on display at the Queensland Museum in Brisbane, exhibited only with the palate clearly in view. Unfortunately, we were not given the opportunity to examine the displayed specimen first-hand. From our restricted observations through the display case, we were able to note that anteriorly the palatines of QMF1752 do not form long anterior processes like in *C. porosus*. In anterior view, we could not see the arching on the premaxillary alveolar processes, but there has been some restorative work performed on the premaxillae of QMF1752 that could potentially have obstructed that feature. Evidently, our observations of QMF1752 through its display case were insufficient to draw meaningful conclusions, other than agree with Willis, Murray & Megirian (1990), Molnar (1991) and Willis & Molnar (1997a) that it is not referable to *C. porosus*. Therefore, we recommend that QMF1752 is studied in detail once again following our taxonomic revision. Until then, we have no other option but treat QMF1752 as Crocodylia *incertae sedis*.

## Conclusions

**Figure 39:**
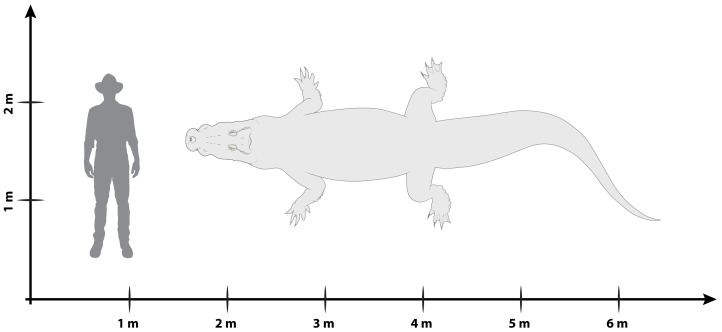
Hypothetical size reconstruction silhouette of *Paludirex vincenti* gen. et sp. nov. The skull shape reconstruction is based on the *Paludirex vincenti* holotype (‘Geoff Vincent’s specimen’ CMC2019-010 + QMF59017). The hypothetical body outline and proportions of *Paludirex vincenti* are based on those of extant crocodylians. The osteoderms are intentionally not depicted since the osteoderm configuration is currently unknown for *Paludirex vincenti*. The human (dark gray silhouette on the left) is 1.80 m tall.

*Pallimnarchus pollens* is herein regarded a *nomen dubium* since its designated lectotype specimen is almost completely missing and, based on its published descriptions and illustrations, deemed to be non-diagnostic. Following this verdict, the majority of fossil specimens formerly assigned to *Pallimnarchus* are temporarily of undetermined generic and specific placement. Only a handful of the examined specimens previously referred to *Pallimnarchus* were sufficiently preserved to allow the establishment of a new crocodylian genus, *Paludirex*. As currently understood, the genus *Paludirex* consists of two large-bodied (Fig. 39) and broad-snouted species – *P. gracilis* and *P. vincenti*. Both *Paludirex* species were presumably semi-aquatic ambush predators likely capable of taking large-sized prey items, as can be inferred from the skull morphology which is largely consistent with that of extant semi-aquatic generalist crocodylians (such as a primarily dorsally opening external narial fenestra, a platyrostral snout, and large and conical teeth). The type species, *P. vincenti*, has a better understood morphology than *P. gracilis* thanks to its more complete holotype specimen. As a consequence, the diagnosis of the genus and differentiation between the two species must temporarily rely on the only shared elements between *P. gracilis* and *P. vincenti*, which are the premaxillae. The proportional differences between the premaxillae of *P. gracilis* and *P. vincenti* are appropriate to justify the specific distinction between them. *Paludirex vincenti* derives from Pliocene (and perhaps Pleistocene) deposits in southeast Queensland, whilst the *P. gracilis* material is from the Pleistocene of northwest Queensland. However, discovery of additional specimens referable to *Paludirex* may change the known spatiotemporal distribution of these two species. Species of *Paludirex* were one of the top predators from the Plio-Pleistocene of Australia, and their extinction is likely a consequence of environmental deterioration prompted by climatic changes that occurred in the late Pleistocene.

## Institutional Abbreviations

AM: Australian Museum, Sydney, New South Wales, Australia (F, fossil)
CMC: Chinchilla Museum Collection, Chinchilla, Queensland, Australia
NTM: Museum and Art Gallery of the Northern Territory, Darwin and Alice Springs, Northern Territory, Australia (P, palaeontology; R, reptile collection)
PVM: Pioneer Valley Museum, Mirani, Queensland, Australia
QM: Queensland Museum, Brisbane, Queensland, Australia (F, fossil)
SAM: South Australian Museum, Adelaide, South Australia, Australia (P, palaeontology)
UQSSAL: University of Queensland Steven Salisbury collection, Brisbane, Queensland, Australia.

## Additional Information and Declarations

### Data Availability

The following information was supplied regarding data availability: Supplementary materials are available at the Dryad Digital Repository: Ristevski, Jorgo et al. (2020), Data from: Australia’s prehistoric ‘swamp king’: revision of the Plio-Pleistocene crocodylian genus *Pallimnarchus* De Vis, 1886, Dryad, Dataset, DOI 10.5061/dryad.8kprr4xkq. The raw CT data used in this study are available at MorphoSource: http://www.morphosource.org/Detail/SpecimenDetail/Show/specimen_id/44104. http://www.morphosource.org/Detail/SpecimenDetail/Show/specimen_id/44130. DOI 10.17602/M2/M158872 CT image series representing QM:F:59017 the holotype’s neurocranium of *Paludirex vincenti*. DOI 10.17602/M2/M158812 CT image series representing CMC:2019-010 holotype remains of *Paludriex vincenti*. The specimens described in this study are accessioned and publicly available at the Chinchilla Museum (CMC2019-010), Pioneer Valley Museum (PVM 89-1072), and the Queensland Museum (QMF11626, QMF17065, QMF17066 and QMF59017).

### New Species Registration

The following information was supplied regarding the registration of a newly described species: Publication LSID: urn:lsid:zoobank.org:pub:402E681F-7C0A-4746-A80B-ADB4870789EC *Paludirex* gen. nov. LSID: urn:lsid:zoobank.org:act:62FE01E1-BC2C-4EC0-8022-0DE6DE9A1A88 *Paludirex vincenti* gen. et sp. nov. LSID: urn:lsid:zoobank.org:act:BF91D49B-642D-404A-BA31-13D53240D056 *Paludirex gracilis* gen. et comb. nov. LSID: urn:lsid:zoobank.org:act:457C24F2-948E-4477-AD42-5504A516FA85.
